# H_2_O_2_–Responsive Anticancer Prodrug: Synthesis, Precision Deuteration in Search of In Vivo Metabolites, and Activation Pathway

**DOI:** 10.1021/acs.jmedchem.5c01975

**Published:** 2025-12-02

**Authors:** Eron Saxon, Dana Stambekova, Thilini Nimasha Fernando Ponnamperumage, Joseph R. Clark, Xiaohua Peng

**Affiliations:** † Department of Chemistry and Biochemistry and the Milwaukee Institute for Drug Discovery, 14751 University of Wisconsin-Milwaukee, 2000 E. Kenwood Boulevard, Milwaukee, Wisconsin 53211, United States; ‡ Department of Chemistry, 5505 Marquette University, 1414 W Clybourn St, Milwaukee 53233, Wisconsin, United States; § Department of Chemistry, University of Tennessee, Knoxville, Tennessee 37996, United States

## Abstract

Boron-based reactive oxygen species (ROS)-activated prodrugs offer a promising strategy for enhancing cancer selectivity, yet their *in vivo* activation remains poorly defined. We report a novel H_2_O_2_-responsive phenylboronic nitrogen mustard prodrug (**10a**) and its precisely deuterated analogue (**10b**), designed to elucidate the activation pathway of ROS-responsive agents. These isotopologues differ only in ethyl substituents—hydrogen (**10a**) versus deuterium (**10b**)—enabling isotope-resolved tracking of metabolic transformations *in vivo*. Co-administration of **10a** and **10b** in triple-negative breast cancer xenograft mice identified two metabolites, providing the first definitive *in vivo* evidence of oxidative deboronation as the primary activation mechanism. Prodrug **10a** exhibited H_2_O_2_-inducible DNA-alkylating activity, selectively inhibited the proliferation of high ROS-expressing MDA-MB-468 cancer cells over nonmalignant MCF-10A cells, markedly suppressed tumor growth without observable toxicity. This study highlights precision deuteration as a mechanistic probe and establishes a platform for rational design and optimization of boron-based anticancer prodrugs.

## Graphical abstract

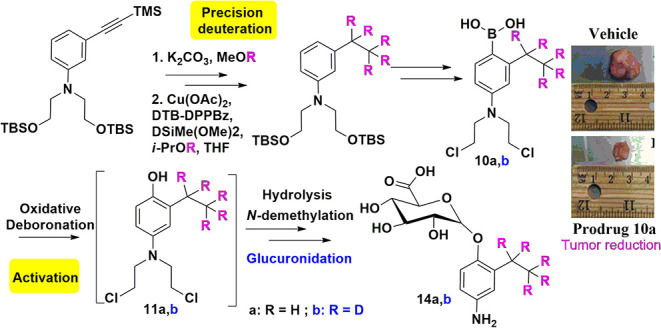

## Introduction

Elevated levels of reactive oxygen species (ROS), particularly hydrogen peroxide (H_2_O_2_), are a biochemical hallmark of many malignancies, driven by dysregulated metabolism and aberrant oncogenic signaling.^1–3^ This intrinsic oxidative stress has been strategically leveraged to develop ROS-activated prodrugs that remain inert under physiological conditions but undergo selective activation in tumor microenvironments, releasing cytotoxic payloads directly at the site of disease.^4–6^ Such systems hold promise for enhancing therapeutic index, minimizing off-target toxicity, and offering new treatment strategies for difficult-to-treat cancers such as triple-negative breast cancer (TNBC), which lack hormone receptors and HER2 amplification.^7,8^ Over the past decade, efforts to exploit ROS as a trigger have led to the development of various chemical motifs, such as boronic acids/esters, sulfur-based functionalities, and selenium-containing scaffolds.^4,9,10^ Among the various ROS-responsive functional groups, arylboronic acids and esters are particularly attractive owing to their high chemoselectivity for H_2_O_2_, undergoing oxidative deboronation to unmask active pharmacophores.^4–6,11–17^ Boron-based ROS-activated prodrugs have demonstrated favorable preclinical profiles; however, the precise *in vivo* activation mechanism—H_2_O_2_-mediated oxidative deboronation—has remained largely speculative. Most studies inferred mechanism only from *in vitro* data, lacking direct structural evidence of metabolites or confirmation of metabolic activation pathways in living systems. This gap in mechanistic understanding limits rational prodrug optimization and complicates clinical translation.

Our group has developed a series of phenylboronic acid/ester-derived nitrogen mustard prodrugs (e.g., structure **A**) that undergo H_2_O_2_-mediated oxidative deboronation to release hydroxyphenyl nitrogen mustard analogues (structures **D** and **E**)—potent DNA-alkylating agents—selectively within ROS-enriched tumor microenvironments (Scheme 1a).^8,15,18–21^ The mechanism of this transformation is well established. In brief, nucleophilic hydroperoxide anion (HOO^–^), generated from H_2_O_2_, attacks the boron center of **A** to form a tetrahedral intermediate **B**. A subsequent 1,2-shift between boron and oxygen yields intermediate **C**, which rapidly hydrolyzes to the active alkylator **D**. In this series of prodrugs, the DNA-alkylating functional group is masked by an arylboronic acid, which withdraws electron density from the aromatic ring and delocalizes the lone pair on the mustard nitrogen, reducing its reactivity. Oxidative conversion of the arylboronic acid to a phenolic hydroxyl group (**D**) restores electron density to the aromatic ring, reactivating the nitrogen mustard and enabling formation of the highly electrophilic aziridinium ion (**E**), which efficiently alkylates DNA (Scheme 1). One representative compound, **FAN-NM-CH**_**3**_, exhibits potent cytotoxicity in cancer cell lines and significant tumor suppression in TNBC xenografts without observable systemic toxicity (Scheme 1b).^8,21^ We further showed that pharmacological ROS enhancement with vitamin C synergistically amplifies the tumor-selective activity of these prodrugs while sparing normal tissues.^21^ Despite these encouraging therapeutic data, the *in vivo* biotransformation profiles of this class remain unverified. Identifying metabolic intermediates is essential for clarifying pharmacokinetic behavior, refining molecular design, and reducing the risk of off-target toxicity.^22,23^

**Scheme 1:**
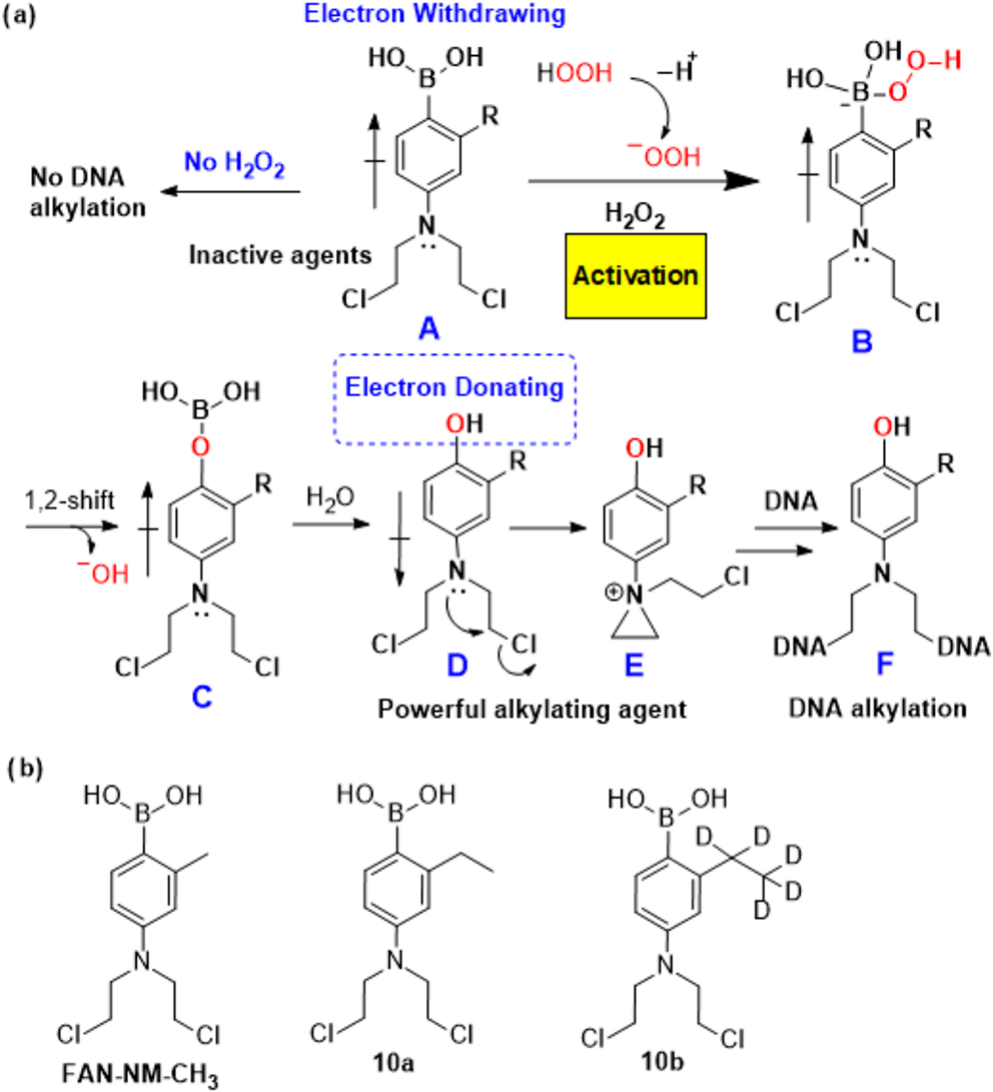
Structures of H_2_O_2_-Responsive Phenylboronic Nitrogen Mustard Prodrugs and Their Activation *via* Oxidative Deboronation Triggered by H_2_O_2_

Stable isotope labeling with deuterium offers a robust and nonradioactive approach to investigate drug metabolism and biochemical transformations in biological systems.^24^ Deuterated analogues retain the physicochemical and biological properties of their hydrogen counterparts, yet are readily distinguished by mass spectrometry, allowing for precise tracing of metabolic fate.^25^ This strategy has been instrumental in mapping metabolic pathways, identifying active metabolites, and improving the metabolic stability of small-molecule drugs. Herein, we report the design, synthesis, and biological evaluation of a H_2_O_2_-activated phenylboronic nitrogen mustard prodrug, **10a**, and its precision-deuterated analogue, **10b** (Scheme 1). Both are ethyl analogues of FAN-NM-CH_3_. While FAN-NM-CH_3_ has been previously validated as a promising prodrug, its deuterated form cannot be efficiently synthesized. The ethyl analogue **10a** allows precision deuteration with five deuterium atoms, enhancing isotope resolution for *in vivo* metabolite tracking, while maintaining structural similarity to methyl to preserve comparable biological behavior. Compounds **10a** and **10b** differ only by isotopic substitution at the ethyl group (^1^H vs ^2^H), enabling isotope-resolved tracking of metabolic transformations *in vivo*. Co-administration of **10a** and **10b** in MDA-MB-468 TNBC xenograft models led to the identification of two distinct metabolites *via* mass spectrometric analysis, providing the first definitive *in vivo* evidence of oxidative deboronation as the primary activation mechanism. This work establishes a mechanistic foundation for the rational development of boron-based anticancer prodrugs and underscores the power of stable isotope labeling in uncovering the biochemical underpinnings of ROS-responsive therapeutics.

## Results and Discussion

### Design and Synthesis

Mixtures of nondeuterated and deuterated drug molecules generate characteristic mass shifts that can be exploited to identify metabolic products with high specificity—without the hazards associated with radiolabeling. This strategy, when paired with high-resolution and sensitive analytical techniques such as LC/MS, LC-TOF-MS, and LC/MS/MS, has proven effective for elucidating *in vivo* metabolic transformations.^26,27^ To enable precise metabolic tracking, we designed and synthesized **10a**, an ethyl analogue of the lead compound **FAN-NM-CH**_**3**_,^8^ along with its isotopologue **10b**, incorporating five deuterium atoms. Structurally, the only modification from **FAN-NM-CH**_**3**_ to **10a/b** involves the isosteric substitution of a methyl group with an ethyl group. Co-administration of a 1:1 mixture of **10a** and **10b** is anticipated to yield a corresponding 1:1 mixture of unlabeled and M+5 labeled metabolites, enabling direct mass spectrometric discrimination.

The synthesis of **10a** started with aniline iodide **1**, which underwent bis-*N*-alkylation to generate **2** (Scheme 2a). Protection of the hydroxyl groups in **2** with TBSCl afforded **3**, which was then subjected to a Sonogashira-type palladium-catalyzed coupling with TMS-acetylene, yielding the alkyne intermediate **4**. Desilylation of **4** using K_2_CO_3_ in methanol provided terminal alkyne **5a**. Transfer hydrogenation of **5a** using isopropanol and a silane donor in the presence of a copper catalyst furnished the ethyl analogue **6a**.^28^ Subsequent removal of the silyl groups using *tert*-butyl ammonium fluoride (TBAF) produced diol **7a**, which was then converted to the corresponding bromide **8a**. Chlorination of the hydroxyl functionalities yielded **9a**, which was subsequently lithiated and borylated to afford the target prodrug **10a**.

**Scheme 2:**
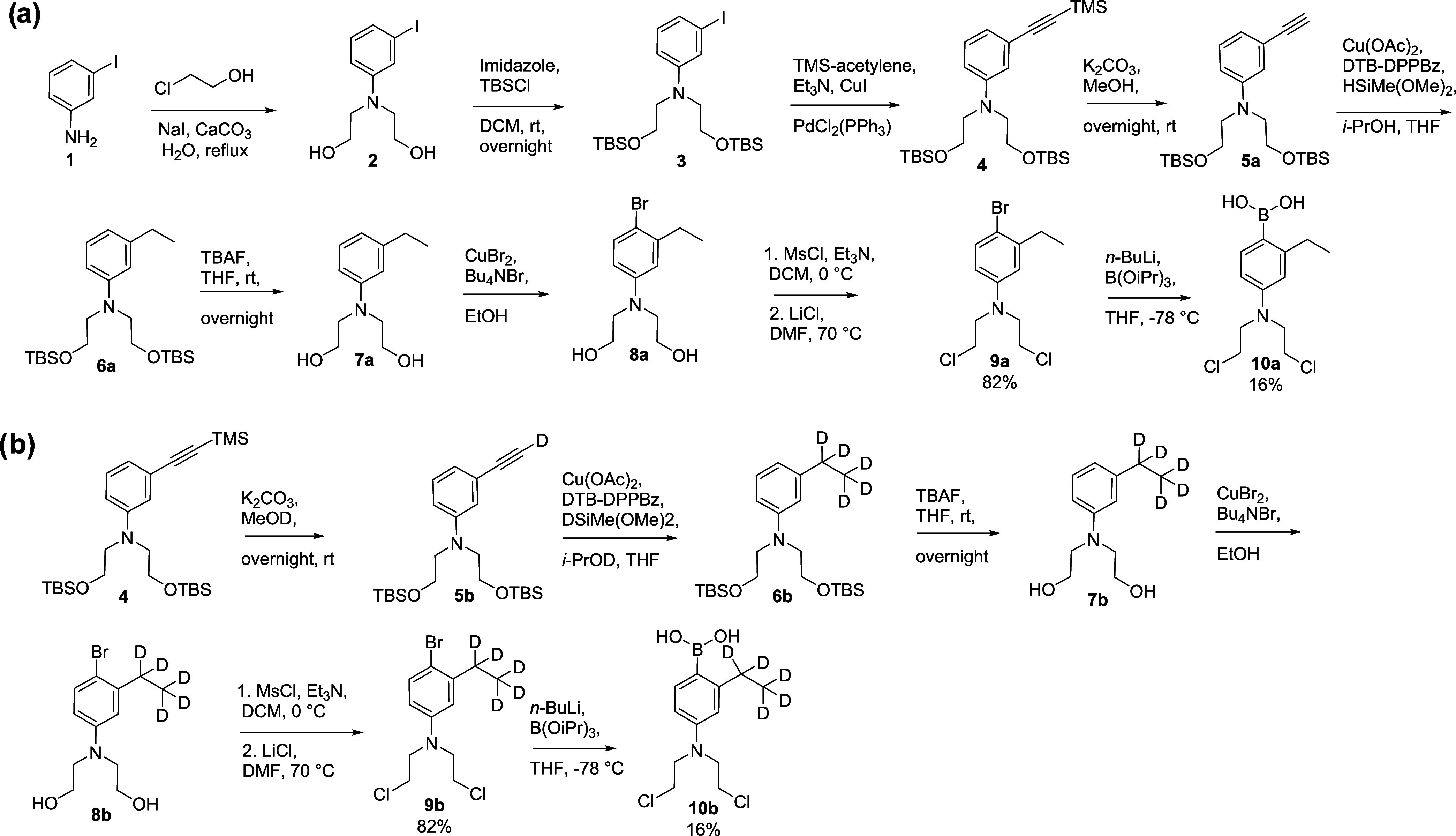
Synthesis of Non-Deuterated Analogue **10a** (a) and Its Deuterated Counterpart **10b** (b) *via* Cu-Catalyzed Transfer Hydrogenation and Deuteration, Respectively

The deuterated analogue **10b** was prepared *via* a similar synthetic route starting from **1** (Scheme 2b). Precision deuteration was achieved *via* Cu-catalyzed transfer hydrodeuteration. Specifically, desilylation of intermediate **4** using deuterated methanol yielded **5b**, which was then subjected to transfer deuteration using deuterated silane and deuterated isopropanol, producing the ethyl-d_5_ compound **6b**.^28,29^ The resulting deuterated intermediate **6b** underwent the same reaction sequence as **6a**, ultimately furnishing **10b**. This approach allowed for regio- and chemoselective introduction of deuterium atoms, facilitating downstream metabolic tracking with high fidelity. The structures of **10a** and **10b** were confirmed by ^1^H NMR, ^13^C NMR, and high-resolution mass spectrometry (HRMS).

### ROS-Dependent DNA Cross-Linking

The chemical reactivity and H_2_O_2_-dependent DNA selectivity of **10a** were evaluated using a synthetic 49-mer DNA duplex (**11**) through polyacrylamide gel electrophoresis (PAGE) to assess interstrand cross-link (ICL) formation (Figures 1 and S1). In the presence of H_2_O_2_, **10a** underwent efficient oxidative deboronation, restoring nitrogen mustard reactivity and enabling robust DNA cross-linking. Significant ICL formation was observed only when **10a** was treated with H_2_O_2_, while markedly lower cross-link yields were detected in its absence (*n* = 2, Figures 1, S2 and S3). This finding is consistent with previous observations for the parent compound **FAN-NM-CH**_**3**_. The ICL formation and selectivity of **10a** were found to be concentration-dependent. A consistent ∼4:1 ratio of ICL yields (with versus without H_2_O_2_) was observed across a concentration range of 0.25 to 3 mM, with a slight decrease in selectivity at higher concentrations (Figure 1a). As shown in Figure 1b, the log­(dose)–response analysis revealed a significantly lower EC_50_ in the H_2_O_2_-treated group (3.28 mM) compared to the untreated group (11.94 mM). ICL formation also displayed clear time dependence, reaching maximal yields after approximately 15 h incubation at rt (*n* = 2; Figures S5–S7).

**Figure 1:**
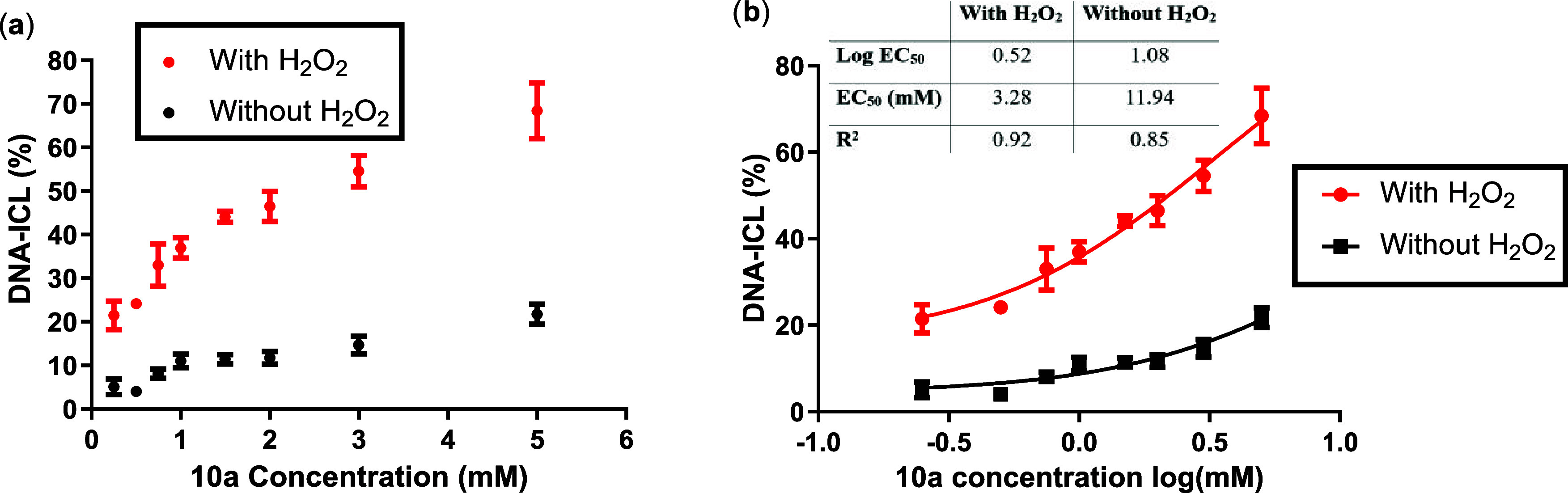
DNA-ICL induced by **10a** as (a) the concentration dependence of ICL formation upon H_2_O_2_-activation and (b) logarithmic function [the data are the average of two independent experiments [trial 1 in Figure S2 and trial 2 in Figure S3].

### *In Vitro* Cytotoxicity and Selectivity

Following the demonstration of H_2_O_2_-inducible DNA ICL formation by **10a**, we assessed its anticancer efficacy and selectivity using a cell viability assay (CellTiter-Glo) in TNBC cell line MDA-MB-468 and normal human mammary epithelial MCF-10A cells. Compound **10a** exhibited significant growth inhibition in MDA-MB-468 cells, with an IC_50_ of 5.1 ± 0.1 μM (*n* = 3; Figure 2), though it was slightly less cytotoxic than the parent FAN-NM-CH_3_ (IC_50_ of 3.4 ± 0.3 μM, Figure S7).^8^ In contrast, a 3-fold higher IC_50_ of 15.2 ± 0.3 μM was observed in the normal MCF-10A cells, suggesting preferential cytotoxicity toward cancer cells. These results confirm the selectivity of **10a** for cancer cells. Comparable results were obtained for **10b** (Figure 2b). Notably, we have previously reported that MDA-MB-468 cells exhibit elevated H_2_O_2_ levels compared to normal MCF-10A cells, which facilitates the selective activation of the boron-masked prodrug in cancer cells.^30^

**Figure 2:**
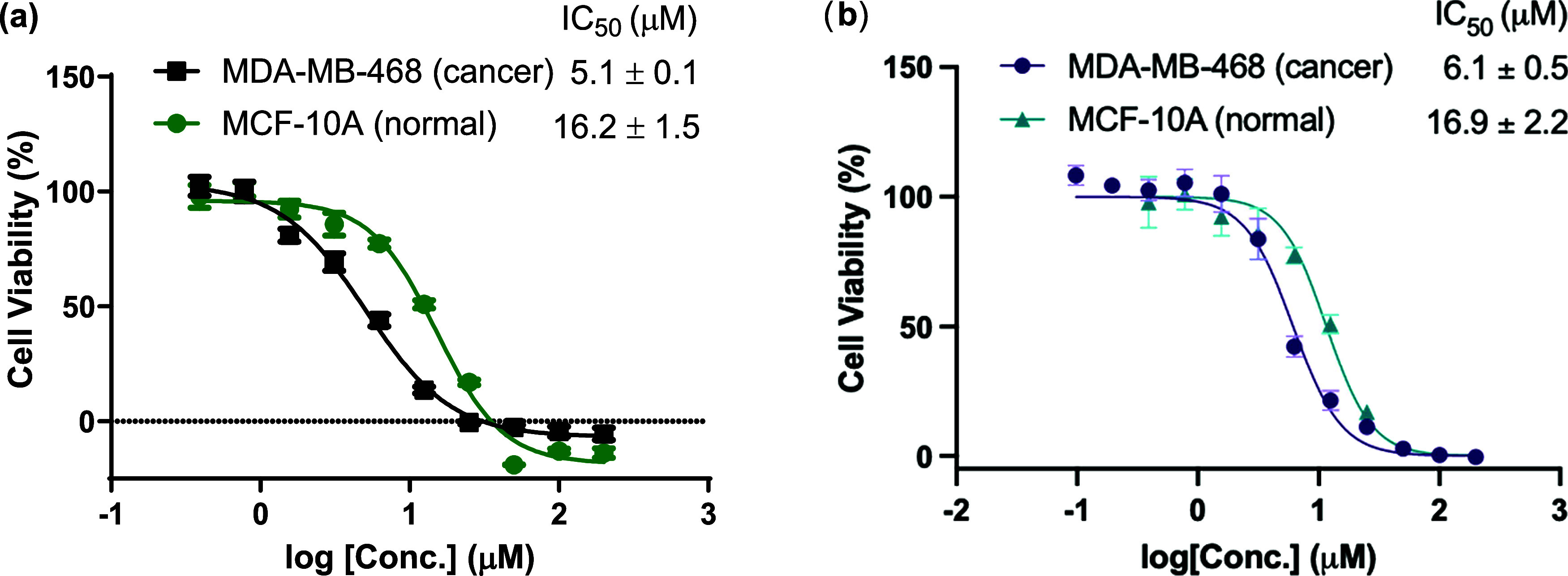
Dose-dependent apoptosis induced by **10a** (a) and **10b** (b) in MDA-MB-468 cancer cells and MCF-10A normal cells. Cells were treated with **10a** or **10b** at concentrations ranging from 0 to 200 μM for 48 h at 37 °C (*n* = 3).

To evaluate the suitability of **10a** and **10b** for *in vivo* metabolite identification, we performed detailed physicochemical studies with **10a**, the only compound available in sufficient quantity. Key properties assessed included aqueous solubility and membrane permeability—both critical determinants of drug absorption and bioavailability. Membrane permeability was measured using the parallel artificial membrane permeability assay (PAMPA) at physiological pH 7.4. Compound **10a** showed good aqueous solubility (74 μM) and moderate permeability (log *P*_e_ = −5.67), comparable to the reference compound naproxen under the same conditions (Table 1). Given that tumor tissues often exhibit a mildly acidic microenvironment due to enhanced glycolytic activity, we also evaluated the permeability of **10a** at pH 6.4 and observed a modest increase (log *P*_e_ = −5.22), suggesting enhanced passive diffusion under acidic conditions. While quantitative measurements for **10b** were not possible due to limited material, several observations support comparable behavior: **10a** and **10b** are isotopologues, for which solubility and permeability are largely determined by electronic structure and intermolecular forces;^31,32^ **10b** displayed qualitatively similar solubility during cytotoxicity assays; and the two compounds showed nearly identical cytotoxicity across multiple cell lines, indicating similar cellular uptake. Taken together, these findings indicate that both **10a** and **10b** possess favorable physicochemical properties suitable for further biological evaluation, including metabolic profiling and *in vivo* efficacy studies.

**Table 1:** Physical and Biophysical Properties of **10a**

	**10a**
solubility (μM) (pH 7.4)^a^	74
permeability (log *P*_e_ (cm/s)) (pH 7.4)^b^	–5.67
permeability (pH 6.4)^b^	–5.22

a The solubility was measured in 80% PBS (pH 7.4) and 20% MeCN (*n* = 3).

b The Permeability was determined using parallel-artificial-membrane permeation assay (PAMPA) at pH 7.4 and pH 6.4 (*n* = 3). Standards verapamil −3.80, naproxen −5.04 and ranitidine −7.16.

### *In Vivo* Efficacy and Selectivity

Having established the *in vitro* cytotoxicity and selectivity of **10a** and **10b**, we next evaluated their *in vivo* anticancer efficacy, selectivity, and potential activation mechanism in a xenograft mouse model (Figure 3). Athymic nude mice were implanted subcutaneously with MDA-MB-468 human TNBC cells. Once tumors became palpable, mice were treated intraperitoneally for 8 weeks with **10a** at 5 mg/kg dose, a dosing regimen paralleling that used for the parent compound **FAN-NM-CH**_**3**_.^8^ In the vehicle-treated control group, tumors exhibited unchecked progression, with weekly increases in tumor volume leading to a mean growth of 2037 ± 422% and a final average tumor size of 2491 ± 1125 mm^3^ by week 8 (Figure 3a,e). In contrast, **10a**-treated mice displayed significantly suppressed tumor growth, culminating in a mean final tumor volume of 264 ± 197 mm^3^—approximately 11% of the control group. The calculated tumor growth inhibition rate (IR), IR(%) = [1 – (mean treated tumor volume/mean control tumor volume)] × 100, was 89% for **10a**, only slightly lower than **FAN-NM-CH**_**3**_ (∼91%), which is consistent with the *in vitro* cytotoxicity study. Tumor weights mirrored this inhibition (201 ± 134 mg versus 924 ± 190 mg in control) (Figure 3b). Importantly, **10a** was well tolerated: no treatment-related mortality or adverse effects occurred, and body weights increased steadily throughout the study (Figure 3c), demonstrating that the tumor-selective activity of **10a** does not compromise overall health. Histological examination of major organs (liver, kidneys, spleen, lungs, heart) revealed no structural abnormalities or pathological changes (Figure 3f,g), further supporting the compound’s safety profile.

**Figure 3:**
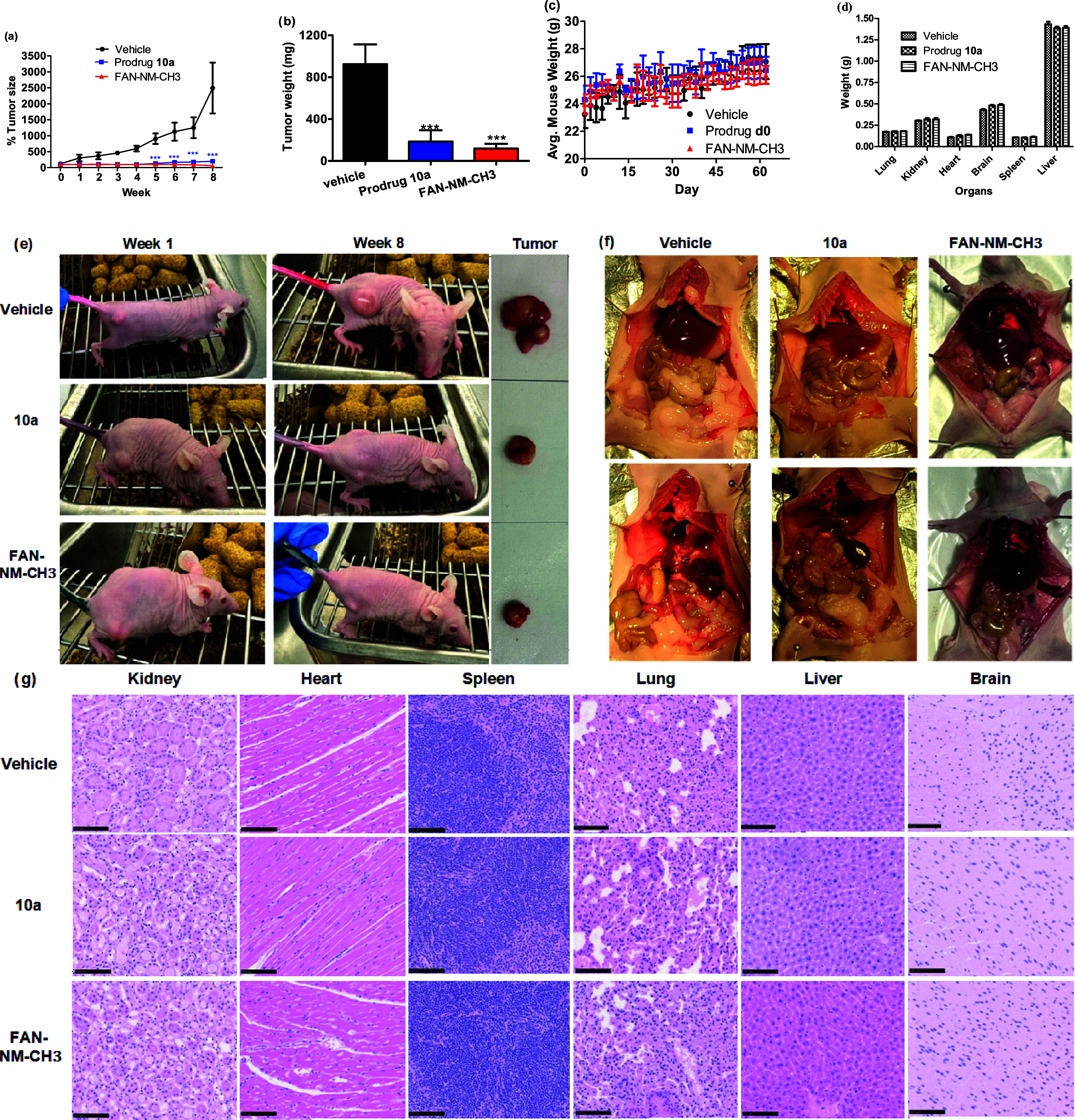
*In vivo* antitumor efficacy and safety. (a) Time-dependent tumor growth monitored by caliper measurement; (b) Tumor weights at the end of the treatment period; (c) Time-dependent changes in body weight measured every other day from week 1 to week 8; (d) Average weights of major organs from xenograft-bearing athymic nude mice; (e) Representative images of mice (week 1 and week 8) and excised tumors after 8 weeks of treatment; (f) Gross appearance of internal organs from mice treated with vehicle or prodrug **10a** or **FAN-NM-CH**_**3**_; (g) H&E staining of organ tissues from vehicle-treated, **10a**-treated, **FAN-NM-CH**_**3**_-treated mice. Xenograft-bearing athymic nude mice were administered intraperitoneally (IP) with either vehicle, prodrug **10a**, or the parent **FAN-NM-CH**_**3**_ at a dose of 5 mg/kg, 5 days per week for 8 weeks. Data are presented as mean ± SD (*n* = 3). Statistical significance was determined using two-way ANOVA (ns = not significant, **P < 0.01, ***P < 0.001 vs control group).

These results are significant for several reasons. First, the potent tumor-growth inhibition with no observable systemic or organ-specific toxicity demonstrates that **10a** selectively suppresses tumor growth while sparing normal tissues. Second, its *in vivo* efficacy is consistent with the DNA-alkylating activity observed in biochemical and cellular assays and is comparable to the parent compound **FAN-NM-CH**_**3**_, suggesting that this prodrug strategy may be broadly applicable to other boronic acid–based DNA-alkylating agents. Third, the pronounced antitumor activity combined with a favorable safety profile highlights the translational potential of **10a** and similar prodrugs. Taken together, these data provide a strong foundation for future studies aimed at optimizing dosing regimens, exploring combination therapies, and elucidating the precise mechanism of activation, which will be addressed in the subsequent metabolite analyses.

### *In Vivo* Evaluation of Metabolites

To elucidate the *in vivo* activation mechanism of the H_2_O_2_-responsive prodrugs **10a** and **10b**, we investigated their metabolic fate in a xenograft mouse model. MDA-MB-468 tumor-bearing athymic nude mice received an intraperitoneal injection of a 1:1 mixture of **10a** (nondeuterated, *d*_0_) and **10b** (deuterated, *d*_5_) at equimolar doses (15 mg/kg each; total dose: 30 mg/kg). Urine samples collected over a 0–24 h period revealed the presence of phase II metabolites **14a** and **14b**, as identified by HRMS (Figure 4 and Table S1). These metabolites arise from glucuronidation of phenolic intermediates and exhibit a diagnostic M/M+5 isotopic pattern, confirming their derivation from **10a** and **10b**, respectively. These end-products are consistent with a pathway initiated by H_2_O_2_-triggered oxidative deboronation of the boronic acid moiety in **10a**/**10b** to yield phenol intermediates **11a**/**11b**, which can undergo either hydrolysis to alcohols **12a**/**12b** followed by *N*-demethylation, or direct *N*-demethylation to **13a**/**13b** (Scheme 3). The resulting **13a**/**13b** serve as substrates for UDP-glucuronosyltransferase (UGT)-catalyzed conjugation with uridine diphosphate glucuronic acid, ultimately forming glucuronide conjugates **14a** and **14b** (Figure 4 and Scheme 3). Although intermediates **11a**,**b**-**13a**,**b** were not detected *in vivo*, likely due to their rapid turnover and concentrations below the sensitivity of our LC–MS method, the proposed sequence is strongly supported by complementary cell-culture metabolism studies in which two key intermediates (**11a**,**b** and **12a**,**b**) were observed (Figures S8 and S9). Detection of both isotopically labeled and unlabeled metabolites therefore provides robust evidence for H_2_O_2_-mediated oxidative deboronation *in vivo*, with the *in vitro* findings offering additional mechanistic confirmation.

**Figure 4:**
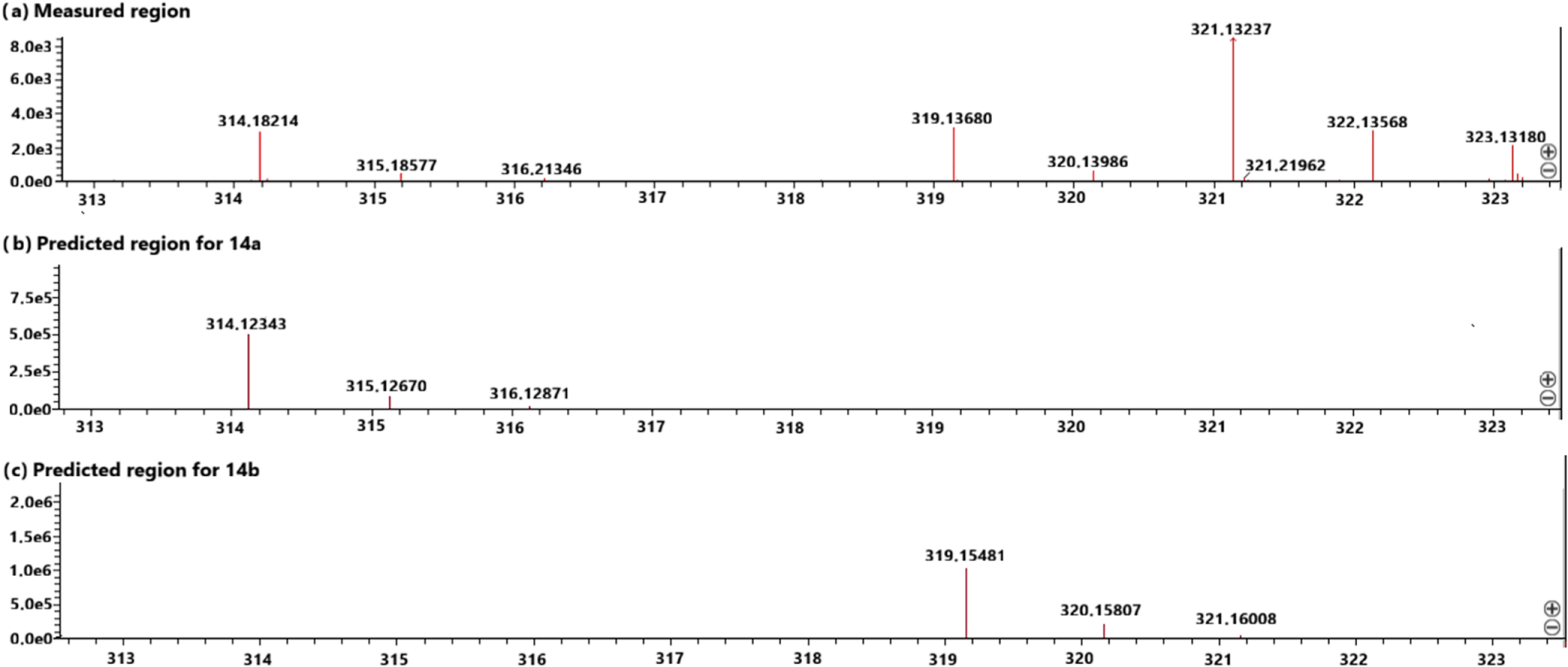
High-resolution mass spectra of metabolites detected in urine from MDA-MB-468 xenograft-bearing athymic nude mice following IP administration of an equimolar mixture of prodrugs **10a** and **10b** (total dose: 30 mg/kg). Urine samples were collected over 0–24 h post-injection and analyzed by HRMS Q-TOF-MS (ESI). Metabolite **14a**: [M + H]^+^, *m*/*z* calcd. 314.12343 for C_14_H_19_NO_7_, found 314.18214; metabolite **14b**: [M + H]^+^, *m*/*z* calcd. 319.15481 for C_14_H_14_D_5_NO_7_, found 319.13680.

**Scheme 3:**
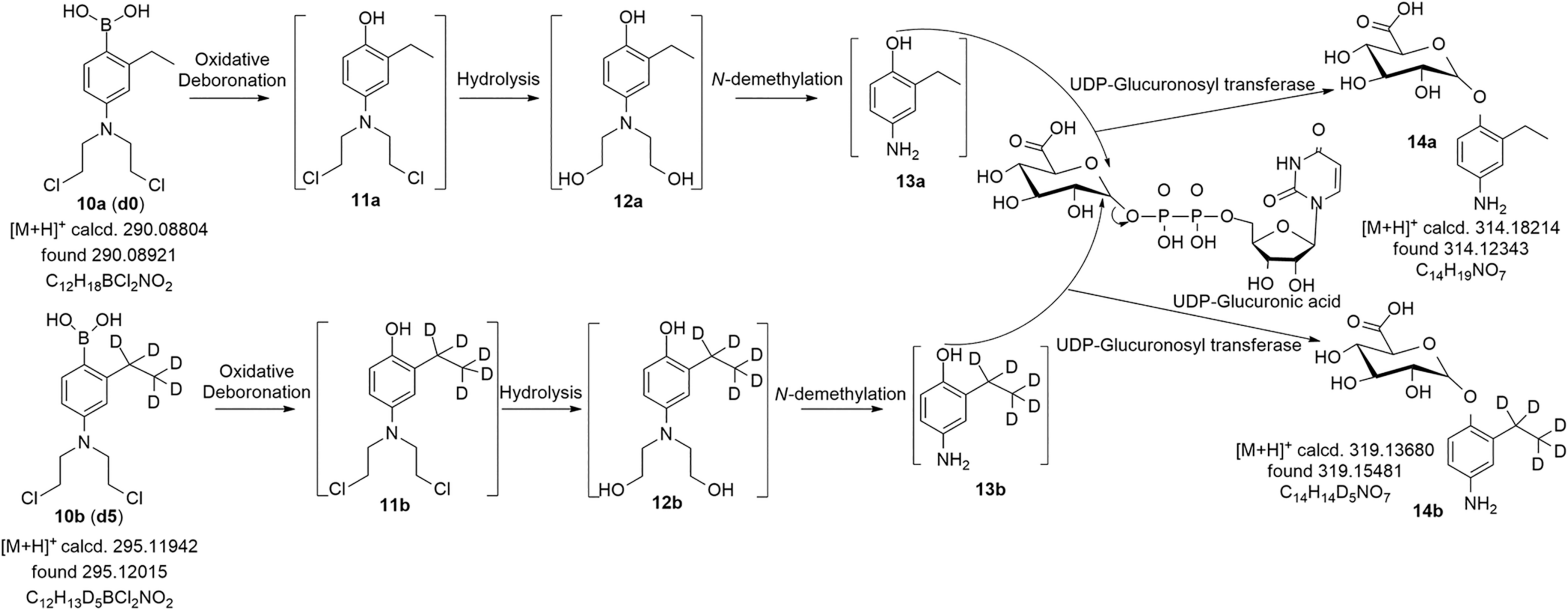
Proposed Metabolic Pathway Leading to Phase II Glucuronide Conjugates **14a** and **14b**, Detected in the Urine of MDA-MB-468 Xenograft-Bearing Athymic Nude Mice Following Intraperitoneal (IP) Administration of a 1:1 Molar Ratio of Prodrugs **10a** and **10b** ^a^

a Initial oxidative deboronation generates intermediates **13a** and **13b**, which undergo subsequent glucuronidation to yield the observed non-deuterated ethyl **14a** and deuterated ethyl **14b** metabolites.

Although the activation mechanism of boron-based prodrugs through H_2_O_2_-mediated oxidative deboronation followed by hydrolysis to release hydroxyl derivatives have been widely observed through *in vitro* experiments,^33–38^ the data was presumed to reflect the *in vivo* selectivity and efficacy of ROS-activated drugs, such as the anticancer effectiveness of prodrug **FAN-NM-CH**_**3**_.^39^ The utility of novel **10a** and **10b** prodrugs, delineates the speculative nature of the prodrugs activation with the discovery of glucuronide metabolites **14a** and **14b**, of which are only possible with hydroxyl (e.g., phenols) substrates through glucuronidation. As a result, these *in vivo* metabolite findings provide significant evidentiary support for *in vitro* observations. To our knowledge, this is the first demonstration of oxidative deboronation as an *in vivo* activation mechanism for boron-containing prodrugs. These findings establish a mechanistic link between ROS-triggered chemical reactivity and biological metabolism. Notably, the formation of nontoxic final metabolites **14a**/**14b** supports the prodrug’s favorable safety profile, minimizing off-target effects and highlighting its promise for selective anticancer therapy.

## Conclusions

In this study, we developed a pair of structurally analogous nondeuterated and deuterated boronic acid–based nitrogen mustard prodrugs, **10a** and **10b**, to investigate H_2_O_2_-triggered activation mechanisms *in vivo*. The ethyl-substituted analogues were synthesized through a modular and deuterium-compatible route enabling precise mass-tagging for metabolite tracing. Compound **10a** exhibited favorable aqueous solubility, pH-sensitive membrane permeability, and robust DNA interstrand cross-linking activity under oxidative conditions, supporting its potential as a redox-responsive anticancer agent. *In vivo*, **10a** significantly inhibited tumor growth in MDA-MB-468 xenograft-bearing athymic nude mice without inducing systemic toxicity or histological damage to major organs. Co-administration of **10a** and **10b** in equimolar amounts enabled high-resolution identification of phase II metabolites (**14a**/**14b**) *via* HPLC-HRMS, providing the first *in vivo* evidence of H_2_O_2_-mediated oxidative deboronation of boron-containing prodrugs. The detected glucuronide conjugates confirm metabolic processing through phenolic intermediates (**13a**/**13b**), establishing a mechanistic link between oxidative tumor microenvironments and prodrug activation. These findings validate the design principles of H_2_O_2_-responsive boron-based prodrugs and open avenues for precision-controlled anticancer therapy *via* redox-selective activation.

## Experimental Section

The following chemicals were purchased from commercial vendors and were used as received: Cu­(OAc)_2_ (99.999% from Alfa Aesar); 1,2-Bis­[bis­[3,5-di­(*t*-butyl)­phenyl]­phosphino]­benzene (DTB-DPPBz) (Waco Pure Cemical Industries) (this ligand was synthesized based on the previously reported procedure),^40^ dimethoxy­(methyl)­silane (TCI); 2-propanol-OD (Millipore Sigma); ethanol-OD (Millipore Sigma); 2-propanol-*d*_8_ (Acros Organic); ethanol (Oakwood Chemical). Dichloromethane (DCM) was distilled from CaH_2_ prior to use. Anhydrous tetrahydrofuran (THF) was purified by an MBRAUN solvent purification system (MB-SPS) or distilled from sodium-wire prior to use. Prior to use, triethylamine (Et_3_N) was distilled over CaH_2_ and stored over 3 Å molecular sieves. Chloroform-*d* (CDCl_3_) was stored over 3 Å molecular sieves. Thin-layer chromatography (TLC) was conducted with Silicycle silica gel 60 Å F254 precoated plates (0.25 mm) and visualized with UV, Iodine and KMnO_4_ stains. Flash chromatography was performed using Silica Flash P60, 40–60 mm (230–400 mesh), purchased from Silicycle. For reactions that required heating (optimization, transfer hydrogenation and deuteration reactions), a PolyBlock for 2-dram vials was used on top of a Heidolph heating/stir plate.

^1^H NMR spectra were recorded on a Varian 300, 400, or 600 MHz or Bruker 500 MHz. spectrometer and are reported in ppm using solvent as an internal standard (CDCl_3_ at 7.26 ppm). Data reported as s = singlet, d = doublet, t = triplet, q = quartet, p = pentet, sxt = sextet, hep = heptet, sep = septet, oct = octet, m = multiplet, br = broad; coupling constant(s) in Hz; integration. ^13^C NMR spectra were recorded on a Varian 76 or 101 MHz spectrometer and are reported in ppm using solvent as an internal standard (CDCl_3_ at 77.16 ppm). ^2^H NMR spectra were recorded on a Varian 61 MHz spectrometer. Labeled solvent impurities were calculated out when reporting isolated yields. Compounds **10a** and **10b** are >95% pure by HPLC analysis.

Low-resolution mass was obtained by Shimadzu LCMS (ESI) 2020. High-resolution mass was obtained by Shimadzu HRMS 9030 Q-TOF-MS (ESI). Solvents (MeCN, H_2_O, MeOH, Formic acid) for mass spectrometry analysis were LCMS grade (Sigma-Aldrich). Mass spectrometry analysis was performed using ACQUITY CSH C18 column (2.1 mm × 50 mm, 1.7 μm particle size). *In vitro* cell viability assays were performed on Tecan Freedom EVO liquid handling system equipped with a 100 nL pin tool (V&P Scientific) and Infinite M1000 (Tecan) plate reader. Physicochemical properties were determined using Corning Costar 96 well UV plate and Infinite M1000 (Tecan) plate reader.

### 2,2′-((3-Iodophenyl)­azanediyl)­bis­(ethan-1-ol) (**2**)

To a flame-dried round-bottom Schlenk flask equipped with Teflon stir bar was added 3-iodoaniline (2.19 g, 10 mmol, 1.0 equiv), 2-chloroethanol (1.6 g, 20 mmol, 2.0 equiv), KI (0.16 g, 0.1 mmol, 0.1 equiv), CaCO_3_ (2.0 g, 20 mmol, 2.0 equiv) and 25 mL of water. After adding all reagents, the reaction mixture was refluxed for 24 h, cooled down to room temperature, quenched with NH_4_Cl (10 mL) and product was extracted with ethyl acetate (4 × 30 mL), organic layers were washed with saturated NaCl (15 mL), and dried over Na_2_SO_4_, solvent was removed under vacuum. The product was isolated to afford **2** as a light-purple solid (0.91 g, 2.96 mmol, 30%) using flash column chromatography (10:1 hexane/ethyl acetate to pure ethyl acetate). ^1^H NMR (600 MHz, CDCl_3_) δ 7.05 (d, *J* = 7.7 Hz, 1H), 7.00 (s, 1H), 6.92 (t, *J* = 8.1 Hz, 1H), 6.64 (dd, *J* = 8.5, 2.6 Hz, 1H), 3.84 (t, *J* = 4.9 Hz, 4H), 3.55 (t, *J* = 4.9 Hz, 4H), 3.38 (br s, 2H). ^13^C NMR (151 MHz, CDCl_3_) δ 149.13, 130.78, 126.03, 121.47, 112.00, 95.78, 60.71, 55.27.

### *N*,*N*-Bis­(2-((*tert*-butyldimethylsilyl)­oxy)­ethyl)-3-iodoaniline (**3**)

To a 100 mL flame-dried round-bottom flask equipped with a Teflon stir bar was added N,N-bis­(2-hydroxoethyl)-3-iodoaniline (0.93 g, 3.03 mmol, 1.0 equiv), CH_2_Cl_2_ (3.03 mL, 1.0 M based on *N*,*N*-bis­(2-hydroxoethyl)-3-iodoaniline substrate), imidazole (0.45 g, 6.7 mmol, 2.2 equiv), *tert*Butyldimethylsilyl chloride (1.0 g, 6.7 mmol, 2.2 equiv). The reaction mixture under N_2_, at room temperature overnight. After 16 h of stirring at room temperature, the reaction was quenched with DI water (10 mL) and extracted with diethyl ether (3 × 25 mL). The organic layers were washed with saturated NaCl (15 mL) and then dried over anhydrous Na_2_SO_4_. The mixture was filtered, and the solvent was removed by rotary evaporation. The crude product was purified by flash column chromatography to give the desired *N*,*N*-bis­(2-*tert*Butyldimethylsilylethoxy)-3-iodoaniline to afford **3** as yellow oil (1.59 g, 2.96 mmol, 100%). ^1^H NMR (600 MHz, CDCl_3_) δ 7.02 (t, *J* = 2.6 Hz, 1H), 6.97–6.94 (m, 1H), 6.89–6.85 (m, 1H), 6.65–6.62 (m, 1H), 3.73 (t, *J* = 6.4 Hz, 4H), 3.46 (t, *J* = 6.4 Hz, 4H), 0.89 (s, 18H), 0.04 (s, 12H). ^13^C NMR (151 MHz, CDCl_3_) δ 149.34, 130.63, 124.68, 120.50, 110.90, 95.84, 60.29, 53.44, 26.09, 18.42, −5.17.

### *N*,*N*-Bis­(2-((*tert*-butyldimethylsilyl)­oxy)­ethyl)-3-((trimethylsilyl)­ethynyl)­aniline (**4**)

To a flame-dried round-bottom flask under N_2_ was added triethylamine (9 mL), which was degassed for 10 min. The aryl halide (0.94 g, 1.76 mmol, 1 equiv), Pd­(PPh_3_)_2_Cl_2_ (24.7 mg, 0.0352 mmol, 0.02 equiv) and CuI (13.4 mg, 0.0732 mmol, 0.04 equiv) were then sequentially added at room temperature. The mixture was stirred for 10 min followed by the addition of TMS-acetylene (2.11 mmol, 1.2 equiv). After 16 h of stirring at room temperature, the reaction was quenched with water (10 mL) and extracted with diethyl ether (3 × 25 mL). The organic layers were washed with water (4 × 10 mL) and then dried over anhydrous Na_2_SO_4_. The mixture was filtered, and the solvent was removed by rotary evaporation. The crude product was purified by flash column chromatography to give the desired aryl substituted propargyl alcohol to afford **4** as yellow oil (1.36 g, 2.69 mmol, 91%). ^1^H NMR (600 MHz, CDCl_3_) δ 7.09 (t, *J* = 7.9 Hz, 1H), 6.79 (s, 1H), 6.75 (d, *J* = 7.1 Hz, 1H), 6.65 (dd, *J* = 8.5, 2.7 Hz, 1H), 3.74 (t, *J* = 6.4 Hz, 4H), 3.48 (t, *J* = 6.4 Hz, 4H), 0.89 (s, 18H), 0.24 (s, 9H), 0.04 (s, 12H). ^13^C NMR (151 MHz, CDCl_3_) δ 147.80, 129.19, 123.76, 119.61, 115.02, 112.18, 106.34, 92.71, 60.43, 53.51, 26.10, 18.43, 0.19, −5.17.

### *N*,*N*-Bis­(2-((*tert*-butyldimethylsilyl)­oxy)­ethyl)-3-ethynylaniline (**5**)

To a 100 mL flame-dried round-bottom flask equipped with a Teflon stir bar was added aniline (0.142 g, 0.28 mmol, 1.0 equiv), potassium carbonate (0.169 g, 1.23 mmol, 10.0 equiv) and anhydrous methanol (0.6 mL, 0.5 M). The reaction was stirred at room temperature overnight. The reaction was quenched with DI water and extracted with diethyl ether (3 × 25 mL). The combined organic layers were washed with saturated NaCl (15 mL), dried over Na_2_SO_4_, and concentrated under vacuum. The crude product was isolated by flash column chromatography using 2% ethyl acetate in hexane to afford **5** as a yellow oil (0.91 g, 2.1 mmol, 78%). ^1^H NMR (300 MHz, CDCl_3_) δ 7.16–7.07 (m, 1H), 6.82 (s, 1H), 6.80–6.66 (m, 2H), 3.74 (t, *J* = 6.1 Hz, 4H), 3.49 (t, *J* = 6.1 Hz, 4H), 3.00 (s, 1H), 0.89 (s, 18H), 0.04 (s, 12H). ^13^C NMR (151 MHz, CDCl_3_) δ 147.86, 129.33, 122.75, 119.66, 115.08, 112.43, 84.83, 76.03, 60.40, 53.54, 26.08, 18.42, −5.19.

### *N*,*N*-Bis­(2-((*tert*-butyldimethylsilyl)­oxy)­ethyl)-3-ethylaniline (**6a**)

In a N_2_ filled glovebox, DTB-DPPBz (8.86 mg, 0.099 mmol, 0.033 equiv), Cu­(OAc)_2_ (45 μL of a 0.2 M solution in THF, 0.009 mmol, 0.03 equiv), and THF (1455 μL) were added to an oven-dried 2- dram vial followed by dropwise addition of dimethoxy­(methyl)­silane (185 μL, 1.5 mmol, 5 equiv). A color change from green/blue to orange was observed while stirring for 15 min. In a separate oven-dried 1- dram vial was added the 3-acetyleneaniline substrate (130.43 mg, 0.3 mmol, 1 equiv), THF (1.5 mL), and 2-propanol (115 μL, 5 eq. based on substrate). The solution in the 1-dram vial was added dropwise over 20 s to the 2-dram vial. The total volume of THF was calculated based on having a final reaction concentration of 1 M based on the 3-acetyleneaniline substrate. The 2-dram vial was capped with a red pressure relief cap, taken out of the glovebox, and stirred for 24 h at the 40 °C temperature. The crude product was isolated by flash column chromatography using 2% ethyl acetate in hexane to afford **6a** as yellow oil (0.114 g, 0.26 mmol, 87%). ^1^H NMR (400 MHz, CDCl_3_) δ 7.11 (t, *J* = 8.0 Hz, 1H), 6.55–6.48 (m, 3H), 3.75 (t, *J* = 6.9 Hz, 4H), 3.49 (t, *J* = 6.8 Hz, 4H), 2.58 (q, *J* = 7.6 Hz, 2H), 1.25–1.21 (m, 3H), 0.90 (s, 18H), 0.05 (s, 12H).^13^C NMR (101 MHz, CDCl_3_) δ 148.09, 145.52, 129.32, 115.53, 111.22, 109.06, 60.39, 53.65, 29.56, 26.08, 18.46, 15.88, −5.17.

### 2,2′-((3-Ethylphenyl)­azanediyl)­bis­(ethan-1-ol) (**7a**)

To a 100 mL flame-dried round-bottom flask equipped with a Teflon stir bar was added N,N-bis­(2-*tert*Butyldimethylsilylethoxy)-3-ethylaniline (0.147 g, 0.33 mmol, 1.0 equiv) and THF (3.36 mL, 0.1M), followed by dropwise addition of TBAF (0.7 mL, 0.67 mmol, 2.0 equiv). The reaction mixture was stirred at room temperature overnight. The reaction was quenched with DI water and extracted with ethyl acetate (4 × 25 mL). The combined organic layers were washed with saturated NaCl (15 mL), dried over Na_2_SO_4_, and concentrated under vacuum. The crude product was isolated by flash column chromatography (10:1 hexane/ethyl acetate to pure ethyl acetate) to afford **7a** as yellow oil (0.057 g, 0.27 mmol, 82%). ^1^H NMR (300 MHz, CDCl_3_) δ 7.21–7.09 (m, 1H), 6.61 (d, *J* = 7.3 Hz, 1H), 6.58–6.47 (m, 2H), 3.92–3.78 (m, 4H), 3.63–3.51 (m, 4H), 3.18 (s, 2H), 2.60 (q, *J* = 7.8 Hz, 2H), 1.26–1.19 (m, 3H). ^13^C NMR (75 MHz, CDCl_3_) δ 148.15, 145.67, 129.40, 116.93, 112.56, 110.36, 61.09, 55.51, 29.52, 15.88.

### 2,2′-((4-Bromo-3-ethylphenyl)­azanediyl)­bis­(ethan-1-ol) (**8a**)

To a 100 mL flame-dried round-bottom flask equipped with a Teflon stir bar was added Bu_4_NBr (0.081 g, 0.25 mmol, 1.0 equiv), N,N-bis­(2-hydroxoethyl)-3-ethylaniline (0.053 g, 0.25 mmol, 1.0 equiv) in EtOH (1.0 mL, 0.25 M based on substrate), CuBr_2_ (0.11 g, 0.5 mmol, 2.0 equiv). The reaction mixture was stirred at room temperature overnight, quenched with Me_4_NOH (0.6 mL), DI water (0.6 mL) and extracted with ethyl acetate (4 × 15 mL). The combined organic layers were washed with saturated NaCl (15 mL), dried over Na_2_SO_4_, solvent was removed under vacuum. The crude product was isolated by flash column chromatography (10:1 hexane/ethyl acetate to pure ethyl acetate) to afford **8a** as yellow oil (0.06 g, 0.21 mmol, 83%). ^1^H NMR (300 MHz, CDCl_3_) δ 7.30 (d, *J* = 8.9 Hz, 1H), 6.53 (s, 1H), 6.38 (d, *J* = 8.4 Hz, 1H), 3.91–3.66 (m, 5H), 3.66–3.26 (m, 5H), 2.67 (q, *J* = 7.4 Hz, 2H), 1.19 (t, *J* = 7.2 Hz, 3H). ^13^C NMR (75 MHz, CDCl_3_) δ 147.42, 143.89, 133.10, 113.77, 112.05, 111.03, 60.69, 55.47, 30.01, 14.69.

### 4-Bromo-*N*,*N*-bis­(2-chloroethyl)-3-ethylaniline (**9a**)

A 25 mL round-bottom flask was charged with **8a** (0.2 g, 0.694 mmol) and Et_3_N (3.06 mL, 21.91 mmol) in 10 mL DCM. Then, methanesulfonyl chloride (0.21 mL, 2.08 mmol) was added by-dropwise at 0 °C. The reaction was stirred at rt for 4h. After completion of the reaction as judged by TLC, the reaction mixture was extracted with DCM and washed with brine three times. The organic layers were combined, dried over Na_2_SO_4_, filtered and concentrated. The resulting residue was dissolved in 10 mL DMF and LiCl (0.12g, 2.08 mmol) was added. After being stirred at 70 °C for 10h, the mixture was extracted with DCM and washed with brine water, dried over Na_2_SO_4_, filtered and concentrated. The crude product was purified by column chromatography (50:1, hexane/EtOAc) to afford **9a** as a yellow oil (0.185g, 82%). ^1^H NMR (500 MHz, CDCl_3_) δ 7.38 (d, *J* = 8.9 Hz, 1H), 6.57 (d, *J* = 3.0 Hz, 1H), 6.43 (dd, *J* = 3.0, 8.9 Hz, 1H), 3.74 (q, *J* = 4.8 Hz, 4H), 3.65 (q, *J* = 4.9 Hz, 4H), 2.73 (q, *J* = 7.2 Hz, 2H), 1.25 (t, *J* = 7.4 Hz, 3H). ^13^C NMR (125 MHz, CDCl_3_) δ 145.65, 144.34, 133.47, 113.17, 111.80, 111.42, 53.54, 40.35, 29.92, 14.49. HRMS-ESI­(+) (*m*/*z*): [M + H]^+^ calcd. for C_12_H_17_BrCl_2_N^+^ 323.99214; found 323.99227.

### (4-(Bis­(2-chloroethyl)­amino)-2-ethylphenyl)­boronic acid (**10a**)

A 25 mL oven-dried flask was charged with **9a** (0.185 g, 0.71 mmol) and dissolved in dry THF (5.0 mL). The solution was cooled to −78 °C under argon. *n*-BuLi (1.2 mL, 2.5 M in hexane) was added slowly at −78 °C within 10 min. After 30 min, B­(OiPr)_3_ (0.47 g, 2.48 mmol) was added to the solution. The reaction mixture was allowed to warm to rt, stirred for another 4 h, quenched with saturated NH_4_Cl solution at 0 °C, then extracted with DCM (75 mL × 3), washed with water, dried over Na_2_SO_4_, and concentrated under vacuum. The residue was purified by column chromatography (10:1 → 1:1 hexane/ethyl acetate) to afford product **10a** as a white solid powder (43 mg, 21%). ^1^H NMR (**10a**, 500 MHz, CDCl_3_) δ 8.16 (d, *J* = 8.6 Hz, 1H), 6.65 (dd, *J* = 2.8, 8.4 Hz, 1H), 6.60 (d, *J* = 2.4 Hz, 1H), 3.85 (t, *J* = 6.8 Hz, 4H), 3.72 (t, *J* = 7.0 Hz, 4H), 3.21 (q, *J* = 7.4 Hz, 2H), 1.39 (t, *J* = 7.4 Hz, 3H). ^13^C NMR (**10a**, 125 MHz, CDCl_3_) δ 155.46, 149.07, 139.90, 112.13, 108.60, 53.21, 40.39, 29.60, 17.77. HRMS-ESI­(+) (*m*/*z*): [M + H]^+^ calcd. for C_12_H_19_NO_2_BCl_2_^+^ 290.08804; found 290.08897.

### *N,N*-Bis­(2-*tert*Butyldimethylsilylethoxy)-3-acetyleneaniline­(*d*1) (**5b**)^41^

Following a previously reported procedure for the terminal deuteration of alkynes, to a flame-dried 100 mL round-bottom flask equipped with a Teflon stir bar was purged with N_2_ and to this was added, aryl alkyne (0.8 g, 1.8 mmol, 1.0 equiv), anhydrous K_2_CO_3_ (0.4 g, 2.8 mmol, 1.5 equiv), and anhydrous CH_3_CN (6.1 mL, 0.3 M) sequentially. After stirring for 30 min, D_2_O (1.7 mL, 91.7 mmol, 50 equiv) was added to the round-bottom flask and the reaction was stirred at room temperature for 48 h. Reaction progress was followed by 1H NMR. Upon completion, the mixture was extracted with diethyl ether (3 × 25 mL). The combined organic extracts were dried over anhydrous Na_2_SO_4_, filtered, and concentrated under vacuum. The crude product was isolated by flash column chromatography using 2% ethyl acetate in hexane using 2% ethyl acetate in hexane to afford **5b** as yellow oil (0.71 g, 1.63 mmol, 91%). ^1^H NMR (400 MHz, CDCl_3_) δ 7.15–7.09 (m, 1H), 6.84–6.80 (m, 1H), 6.80–6.75 (m, 1H), 6.69 (dd, *J* = 8.6, 2.7 Hz, 1H), 3.74 (t, *J* = 6.4 Hz, 4H), 3.49 (t, *J* = 6.3 Hz, 4H), 2.99 (s, 0.09H), 0.89 (s, 18H), 0.03 (s, 12H). ^13^C NMR (400 MHz, CDCl_3_) δ 147.88, 129.28, 122.77, 119.69, 115.13, 112.47, 84.37, 75.96, 60.42, 53.56, 26.06, 18.42, −5.21.

### *N*,*N*-Bis­(2-((*tert*-butyldimethylsilyl)­oxy)­ethyl)-3-(ethyl-*d*5)­aniline­(*d*5) (**6b**)

In a N_2_ filled glovebox, DTB-DPPBz (8.86 mg, 0.099 mmol, 0.033 equiv), Cu­(OAc)_2_ (45 μL of a 0.2 M solution in THF, 0.009 mmol, 0.03 equiv), and THF (1455 μL) were added to an oven-dried 2- dram vial followed by dropwise addition of dimethoxy­(methyl)­silane-D (185 μL, 1.5 mmol, 5 equiv). A color change from green/blue to orange was observed while stirring for 15 min. In a separate oven-dried 1- dram vial was added the 3-acetyleneaniline substrate­(*d*1) (130.43 mg, 0.3 mmol, 1 equiv), THF (1.5 mL), and 2-propanol-*d*_8_ (115 μL, 5 eq. based on substrate). The solution in the 1-dram vial was added dropwise over 20 s to the 2-dram vial. The total volume of THF was calculated based on having a final reaction concentration of 1 M based on the 3-acetyleneaniline substrate. The 2-dram vial was capped with a red pressure relief cap, taken out of the glovebox, and stirred for 24 h at the 40°C temperature. The crude product was isolated by flash column chromatography using 2% ethyl acetate in hexane to afford **6b** as yellow oil (0.12 g, 0.27 mmol, 90%). ^1^H NMR (400 MHz, CDCl_3_) δ 7.15–7.08 (m, 1H), 6.52 (s, 2H), 6.51–6.48 (m, 1H), 3.75 (t, *J* = 6.8 Hz, 4H), 3.49 (t, *J* = 6.8 Hz, 4H), 2.54 (s, 0.13H), 1.22–1.19 (m, 0.20H), 0.90 (s, 18H), 0.05 (s, 12H). ^13^C NMR (400 MHz, CDCl_3_) δ 148.11, 145.45, 129.32, 115.55, 111.24, 109.07, 60.40, 53.66, 30.48, 26.09, 18.47, 15.11, −5.17. ^2^H NMR (400 MHz, CDCl_3_) δ 2.54 (s, 1.87D), 1.18 (s, 2.82D).

### *N*,*N*-Bis­(2-hydroxoethanol)-3-ethylaniline­(*d*5*)* (**7b**)

To a 100 mL flame-dried round-bottom flask equipped with a Teflon stir bar was added *N*,*N*-bis­(2-*tert*Butyldimethylsilylethoxy)-3-ethylaniline-*d5* (0.147 g, 0.33 mmol, 1.0 equiv) and THF (3.36 mL, 0.1M), followed by dropwise addition of TBAF (0.7 mL, 0.67 mmol, 2.0 equiv). The reaction mixture was stirred at room temperature overnight. The reaction was quenched with DI water and extracted with ethyl acetate (4 × 25 mL). The combined organic layers were washed with saturated NaCl (15 mL), dried over Na_2_SO_4_, and concentrated under vacuum. The crude product was isolated by flash column chromatography using 100% ethyl acetate to afford **7b** as yellow oil (0.07 g, 0.33 mmol, 99%). ^1^H NMR (300 MHz, CDCl_3_) δ 7.21–7.09 (m, 1H), 6.60 (d, *J* = 7.2 Hz, 1H), 6.57–6.44 (m, 2H), 4.47–3.59 (m, 6H), 3.59–3.50 (m, 4H), 2.56 (s, 0.13H), peak around 1.21 is overlapping with grease impurity. ^13^C NMR (300 MHz, CDCl_3_) δ 148.01, 145.54, 129.35, 116.71, 112.30, 110.13, 60.98, 55.59, 30.43, 14.26. ^2^H NMR (400 MHz, CDCl_3_) δ 2.57 (s, 1.87D), 1.21 (s, 2.82D).

### 2,2′-((4-Bromo-3-ethylphenyl)­azanediyl)­bis­(ethan-1-ol)­(*d*5) (**8b**)

To a 100 mL flame-dried round-bottom flask equipped with a Teflon stir bar was added Bu_4_NBr (0.086 g, 0.27 mmol, 1.0 equiv), *N*,*N*-bis­(2-hydroxoethyl)-3-ethylaniline-*d*5 (**8b**, 0.057 g, 0.27 mmol, 1.0 equiv) in EtOH (1.1 mL, 0.25 M based on substrate). CuBr_2_ (0.12 g, 0.5 mmol, 2.0 equiv). The reaction mixture was stirred at room temperature overnight, quenched with Me_4_NOH (0.6 mL), DI water (0.6 mL) and extracted with ethyl acetate (4 × 15 mL). The combined organic layers were washed with saturated NaCl (15 mL), dried over Na_2_SO_4_ solvent was removed under vacuum. The crude product was isolated by flash column chromatography using 70% ethyl acetate in hexane to afford **8b** as yellow oil (0.064 g, 0.22 mmol, 81%). ^1^H NMR (300 MHz, CDCl_3_) δ 7.30 (d, *J* = 8.7 Hz, 1H), 6.52 (s, 1H), 6.37 (d, *J* = 8.3 Hz, 1H), 4.16 (s, 2H), 3.82–3.67 (m, 4H), 3.55–3.45 (m, 4H), 2.64 (s, 0.13H), signal around 1.21 overlaps with grease impurity peak. ^13^C NMR (300 MHz, CDCl_3_) δ 147.39, 143.82, 133.08, 113.70, 111.99, 110.94, 60.66, 55.45, 29.43, 14.22. ^2^H NMR (400 MHz, CDCl_3_) δ 2.57 (s, 1.87D), 1.21 (s, 2.82D).

### 4-Bromo-*N*,*N*-bis­(2-chloroethyl)-3-(ethyl-*d*_5_)­aniline­(*d*5) (**9b**)

A 25 mL round-bottom flask was charged with **8b** (0.202 g, 0.694 mmol) and Et_3_N (3.06 mL, 21.91 mmol) in 10 mL DCM. Then, methanesulfonyl chloride (0.21 mL, 2.08 mmol) was added by-dropwise at 0 °C. The reaction was stirred at rt for 4h. After completion of the reaction as judged by TLC, the reaction mixture was extracted with DCM and washed with brine three times. The organic layers were combined, dried over Na_2_SO_4_, filtered and concentrated. The resulting residue was dissolved in 10 mL DMF and LiCl (0.12g, 2.08 mmol) was added. After being stirred at 70 °C for 10 h, the mixture was extracted with DCM and washed with brine water, dried over Na_2_SO_4_, filtered and concentrated. The crude product was purified by column chromatography (50:1, hexane/EtOAc) to afford **9b** as a yellow oil (0.187 g, 82%). ^1^H NMR (300 MHz, CDCl_3_) δ 7.30 (d, *J* = 8.7 Hz, 1H), 6.52 (s, 1H), 6.37 (d, *J* = 8.3 Hz, 1H), 4.16 (s, 2H), 3.82–3.67 (m, 4H), 3.55–3.45 (m, 4H), 2.64 (s, 0.13H), signal around 1.21 overlaps with grease impurity peak. ^13^C NMR (75 MHz, CDCl_3_) δ 147.39, 143.82, 133.08, 113.70, 111.99, 110.94, 60.66, 55.45, 29.43, 14.22. ^2^H NMR (61 MHz, CDCl_3_) δ 2.57 (s, 1.87D), 1.21 (s, 2.82D). HRMS-ESI­(+) (*m*/*z*): [M + H]^+^ calcd. for C_12_H_12_D_5_BrCl_2_N^+^ 329.03126; found 329.00384.

### (4-(Bis­(2-chloroethyl)­amino)-2-(ethyl-*d*_5_)­phenyl)­boronic acid­(*d*5) (**10b**)

A 25 mL oven-dried flask was charged with **9b** (0.189 g, 0.71 mmol) and dissolved in dry tetrahydrofuran (THF) (5.0 mL). The solution was cooled to −78 °C under argon. *n*-BuLi (1.2 mL, 2.5 M in hexane) was added slowly at −78 °C within 10 min. After 30 min, B­(OiPr)_3_ (0.47 g, 2.48 mmol) was added to the solution. The reaction mixture was allowed to warm to rt, stirred for another 4 h, quenched with saturated NH_4_Cl solution at 0 °C, then extracted with DCM (75 mL × 3), washed with water, dried over Na_2_SO_4_, and concentrated under vacuum. The residue was purified by column chromatography (10:1 → 1:1 hexane/ethyl acetate) to afford product **10b** as a white solid powder (44.0 mg, 21%). ^1^H NMR (**10b**, 500 MHz, CDCl_3_) δ 8.16 (d, *J* = 8.9 Hz, 1H), 6.64 (dd, *J* = 2.3, 8.4 Hz, 1H), 6.59 (d, *J* = 3.0 Hz, 1H), 3.84 (t, *J* = 6.8 Hz, 4H), 3.73 (t, *J* = 7.4 Hz, 4H). ^13^C NMR (**10b**, 125 MHz, CDCl_3_) δ 155.40, 149.07, 139.88, 112.11, 108.60, 53.21, 40.39. HRMS-ESI­(+) (*m*/*z*): [M + H]^+^ calcd. for C_12_H_14_D_5_NO_2_BCl_2_^+^ 295.11942; found 295.11965.

### General Method for Cell Viability (Toxicity) Assay: Dose Response (MDA-MB-468)

Cell cultures were grown in L-15 media supplemented with 10% FBS at 37 °C in a humidified atmosphere. The cells were inoculated into 96-well microtiter plates in 40 μL plating densities ranging from 3500 to 10,000 cells/well. After cell inoculation, the microtiter plates were incubated at 37 °C for 1 h prior to the addition of the Prodrug. Prodrug molecules were dissolved in DMSO at 20 mM and serially diluted 10–12 times by 50%. 400 nanoliters of the serially diluted solutions were added to the cell plate (1:100 dilution) using Tecan Freedom EVO liquid handling system equipped with a 100 nL pin tool (V&P Scientific) resulting in final concentrations of 0.39 to 400 μM or 0 μM as the control. Following addition, the plates were incubated for 48 h at 37 °C. Then, aliquots of 30 μL CellTiter-Glo (Promega) were added to the wells, and the resulting plate was incubated for 15 min. The plate was then measured for luminescence by Infnite M1000 (Tecan) plate reader.

### *In Vivo* Efficacy and Selectivity Study with MDA-MB-468 Xenograft Models

Eight-week-old immunodeficient female nude mice were anesthetized with isoflurane and subcutaneously inoculated in the flank with 7.5 × 10^6^ MDA-MB-468 cells suspended in 100 μL of a 1:1 mixture of Matrigel and Dulbecco’s Modified Eagle Medium (DMEM). All cell lines were sourced from the American Type Culture Collection (ATCC) and confirmed negative for bloodborne pathogens. Mice were monitored daily for tumor development and weighed weekly prior to treatment. Once palpable xenograft tumors were established (typically within 1 week), animals were randomized into treatment or control groups (*n* = 3 per group). Mice received daily intraperitoneal (IP) injections of either compound **10a** formulated in PBS/PEG400/DMSO (19:19:2) or vehicle alone, administered at a dose of 5.0 mg/kg in a maximum volume of 100 μL per injection, for seven consecutive weeks. Tumor volumes were measured weekly using electronic calipers, and body weights were recorded concurrently (Figure S7a). At study termination, all tumors, hearts, lungs, livers, kidneys, brains, and spleen were harvested, weighed, and stored in −80 °C for further analysis (Figure S7b,c). For histological morphometry and apoptosis analysis, major organs, including liver and kidney, were fixed with 10% formalin, later embedded in paraffin and cut into 5-μm-thick sections and stained with hematoxylin and eosin (Figure S7d). Stained slides were Imaged using Hamamatsu WSI imager and analyzed using NDP.view2 software.

All animal procedures were approved by the University of Wisconsin–Milwaukee Animal Care and Use Committee (IACUC, protocol 22–23-#21, titled “Investigation of ROS-activated anticancer prodrugs in mice”) and conducted in accordance with the guidelines of the Animal Resource Center (ARC) and the University Safety & Assurances offices, including Animal Care, Environmental Protection and Laboratory Safety divisions.”

### *In Vitro* Cellular Metabolite Identification

MDA-MB-468 cells (≈ 2 × 10^6^) were seeded in a 6-well plate and allowed to adhere overnight. The next day, the culture media (1 mL) was replaced, and cells were treated with equimolar concentrations of the **d0** and **d5** compounds (total 100 μM, 10 μL), or with DMSO vehicle or left untreated as controls. After incubation for the indicated time points (0–48 h), cells were scraped and transferred to 2 mL microcentrifuge tubes. Methanol (1 mL) was added, and samples were sonicated for 10 min, centrifuged at 10,000 rpm for 5 min, and the supernatant was filtered filtered through Spin-X centrifuge tubes. The filtrates were analyzed by Shimadzu HRMS 9030 Q-TOF.

### *In Vivo* Determination of Metabolites from Xenograft Models

Eight-week-old female athymic nude mice (immunodeficient) were anesthetized with isoflurane and subcutaneously inoculated in the flank with 7.5 × 10^6^ MDA-MB-468 cells in 100 μL of a 1:1 mixture of Matrigel and Dulbecco’s Modified Eagle Medium (DMEM). Cells were sourced from the American Type Culture Collection (ATCC) and certified free of bloodborne pathogens. Mice were monitored daily for tumor development and weighed weekly. Upon establishment of palpable xenografts (typically one-week postinoculation), animals were randomized into treatment (*n* = 3) and control (*n* = 3) groups. For metabolite profiling, mice were individually housed in Labsand-lined cages (Labsand purchased from BrainTree Scientific, inc.). The treatment group received an IP injection of a 1:1 mixture of compounds **10a** (*d*_0_) and **10b** (*d*_5_) (100 μL) at a combined dose of 40 mg/kg, formulated in DMSO/PBS (1×):PEG400 at a ratio of 2:19:19. The control group received the corresponding vehicle (PBS/PEG400/DMSO, 19:19:2). Food and water were provided ad libitum. Urine and Feces were collected separately at two intervals: 0–24 h and 24–48 h. All collected samples were subsequently stored in −80 °C.

#### Procedures for Metabolite Analysis

Fecal samples (0.4 g) were thawed on ice from −80 °C. The feces were mixed and homogenized with a pestle in 10 mL centrifuge tube with LCMS grade MeOH (2 mL), then centrifuged for 5 min at 10,000*g*. The supernatant was transferred to a separate 2 mL centrifuge tube and dried. The resulting feces were subjected to SPE C-18 (Supelco Discovery DSC-18Lt SPE Tube) by initially using 5 mL 18 LCMS grade water and subsequently eluted with LCMS grade MeOH. The organic eluant was collected and dried. The resulting feces mixture was filtered by size exclusion (Amicon Ultra Centrifugal Filter, 3 kDa MWCO). Then the solution was transferred to a sample vial with 300 μL insert and analyzed by Shimadzu HRMS 9030 Q-TOF. Urine samples were thawed on ice from −80 °C, vortexed for 5 s and blended in aliquots of 20 μL MeCN. The samples were vortexed for 15 s and centrifuged for 10 min at 10,000*g*. The supernatant was transferred to a clean microcentrifuge tube and diluted 10× with 80:20 H_2_O/MeCN. The resulting solution was filtered by size exclusion (Amicon Ultra Centrifugal Filter, 3 kDa MWCO) at 11k × g.

## Supplementary Material





## Data Availability

Experimental data are available from the corresponding author upon reasonable request.

## Notes

Phosphorimage autoradiogram of the denaturing-PAGE image of the DNA cross-link assay; dose-dependent apoptosis induced by FAN-NM-CH_3_; *in v*i*vo* determination of metabolites from Xenograft models; HRMS of the metabolites detected in MDA-MB-468 cell culture; HRMS spectra of mixture of **10a** and **10b**; NMR spectra of synthesized molecules; HRMS spectra of synthesized molecules **10a** and **10b**; purity of final products **10a** and **10b** (PDF)
Molecular formula strings (CSV)

## Abbreviations Used

ATCC: the American Type Culture Collection
CDCl_3_: chloroform-*d*
DCM: dichloromethane
DIFLD: drug-induced fatty liver disease
DMEM: Dulbecco’s Modified Eagle Medium
DMF: *N*,*N*-dimethylformamide
DMSO: dimethyl sulfoxide
DTB-DPPBz: 1,2-bis­[bis­[3,5-di­(*t*-butyl)­phenyl]­phosphino]­benzene
H&E: hematoxylin and eosin
HRMS: high-resolution mass spectrometry
ICL: interstrand cross-link
I.P.: intraperitoneal
LC/MS: liquid chromatography–mass spectrometry
LC/MS/MS: liquid chromatography-tandem mass spectrometry
LC-TOF-MS: liquid chromatography-time-of-flight mass spectrometry
NMR: nuclear magnetic resonance
PAGE: polyacrylamide gel electrophoresis
PAMPA: parallel artificial membrane permeability assay
Q-TOF-MS: quadrupole time-of-flight mass spectrometry
ROS: reactive oxygen species
TBAF: tetrabutylammonium fluoride
TBSCl: *tert*-butyldimethylsilyl chloride
THF: tetrahydrofuran
TLC: thin-layer chromatography
TMS: trimethylsilane
TNBC: triple-negative breast cancer
UDP: uridine diphosphate
UGT: UDP-glucuronosyltransferase
UV: ultraviolet
