# Supplementary material for: H2O2–Responsive Anticancer Prodrug: Synthesis, Precision Deuteration in Search of In Vivo Metabolites, and Activation Pathway
Source: J Med Chem. 2025 Dec 2;68(23):25026–37. doi: 10.1021/acs.jmedchem.5c01975 (PMC12703749; doi:10.1021/acs.jmedchem.5c01975)
Supplement: Supplementary file 1 [file jm5c01975_si_001.pdf]

Supporting Information

H_2O_2-Responsive Anticancer Prodrugs: Synthesis, Precision Deuteration in Search of *in vivo* Metabolites, and Activation Pathway

Eron Saxon,[a,d] Dana Stambekova,[b,d] Thilini Nimasha Fernando Ponnamperumage,[a] Joseph R. Clark,[c*] Xiaohua Peng[a*]

[a] *Department of Chemistry and Biochemistry and the Milwaukee Institute for Drug Discovery, University of Wisconsin-Milwaukee, 3210 N. Cramer St., Milwaukee, 2000 E. Kenwood Boulevard, Milwaukee, WI 53211*
[b] *Department of Chemistry, Marquette University, 1414 W Clybourn St, Milwaukee, WI*
[c] *Department of Chemistry University of Tennessee, Tennessee 37996, U.S.A*

[d] E.S. and D.S. contributed equally to this work.

pengx@uwm.edu

jclar198@utk.edu

Table of Contents

1. DNA-ICL Studies 3
   1.1 General Methods for DNA Cross-linking 3
   1.2 Concentration-dependent DNA-ICL formation 4
   1.3 Time-dependent DNA-ICLformation 6
2. Dose-dependent apoptosis induced by FAN-NM-CH_3 8
3. In Vivo Determination of metabolites from Xenograft models 9
4. HRMS of the metabolites detected in MDA-MB-468 cell culture 10
5. HRMS spectra of a mixture of 10a and 10b 12
6. NMR spectra of synthesized molecules 13
7. HRMS spectra of synthesized molecules 10a and 10b 46
8. Purity of final Products: 10a and 10b 48
9. References 49

1. DNA-ICL Studies

1.1 General Methods for DNA Cross-linking

Concentration-dependent DNA cross-linking with duplex DNA. ICL formation and cross-linking yields were analyzed using denaturing polyacrylamide gel electrophoresis (PAGE) and imaged by phosphorimager analysis. The DNA cross-linking abilities of the theranostic molecules were investigated by reactions with a ^{32}P-labeled 49-mer ODN (**11**, Figure S1) then subjected to 20% denaturing PAGE analysis. The ^{32}P-labelled ODN (1.0 μM) was annealed with 1.5 equiv. of the complementary strand by heating to 90 °C for 5 min in PBS (10 mM, pH 8) and 100 mM NaCl, followed by slow-cooling to rt overnight. The ^{32}P-labeled duplex DNA (2 μL, 1.0 μM) was mixed with 1.0 M NaCl (2 μL), 100 mM potassium phosphate (2 μL, pH 8), H_2O_2 (2 equivalence in 2 μL) and 2.5 mM to 50 mM **10a** (2 μL, resulted in a concentration range of 250 μM to 5 mM) and H_2O to give a final volume of 20 μL. The reaction mixture was incubated at room temperature for 16 h and quenched by an equal volume of 90% formamide loading buffer, then subjected to 20% denaturing polyacrylamide gel analysis.

Interstrand cross-link formation and kinetics study with duplex DNA. Aliquots (final concentration: 0.1 μM 32P-labeled oligonucleotide duplex 11, 100 mM NaCl, 10 mM potassium phosphate buffer (pH 8), and 0.5 mM of 10a with H_2O_2 (1mM, 2 equivalence) were incubated at rt for various time periods and quenched by 90% formamide loading buffer and stored at -20 °C until the result mixture for 20% denaturing PAGE analysis.

Note on Smearing Bands in Denaturing PAGE. Slower-migrating bands in the denaturing PAGE autoradiograms (Figures S2–S7) are especially obvious when DNA ICL yields are high. As shown in Figure S4, these bands consistently appear when ICL yields exceed ~8.9% but are absent or much less apparent when yields fall below ~7.8%, even in the presence of H_2O_2. This pattern indicates that the bands are not artifacts but instead reflect heterogeneous ICL products. Because ICL formation is non-selective, multiple alkylation sites can be involved, producing products with varying alkylation degrees. The resulting alkylated DNA species carry additional positive charges, which slow their electrophoretic migration and give rise to the observed lower-mobility bands. Similar bands were also observed in previous studies.[1]

```
                6            14              22       27
5'-dGCCTAGTTCTTTTAATTACTTGCAATGCAAGTAATTAAAGCTTGATCTG-3'  (11a)
3'-dCGGATCAAGAAAATTAATGAACGTTACGTTCATTAATTTCGAACTAGAC-5'  (11b)
                                  11
```

Figure S1. The synthetic 49-mer DNA duplex **11**.

1.2 Concentration-dependent DNA-ICL formation

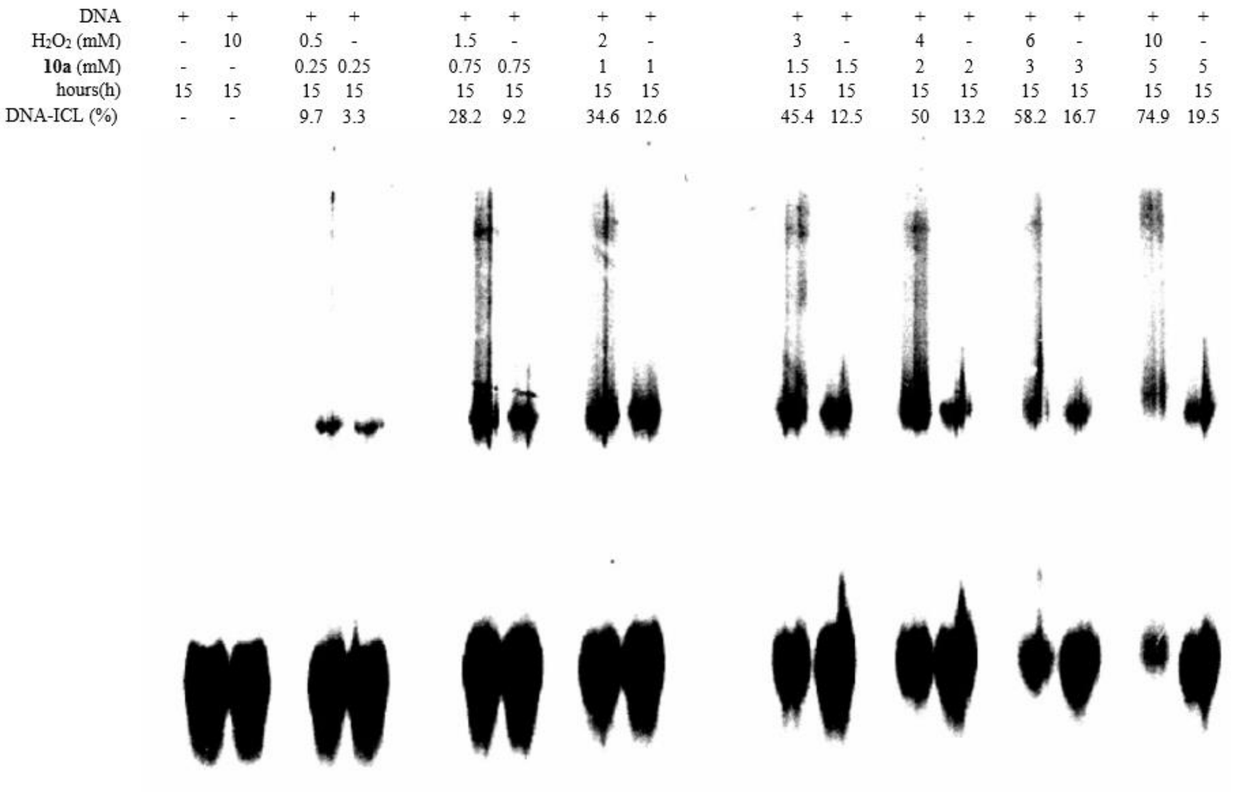

Figure S2. Trial 1 concentration dependent study of DNA cross-link formation induced by 10a upon H_2O_2-activation. Phosphorimage autoradiogram of 20% denaturing PAGE analysis of ICL formation with duplex DNA 11 and different concentration of compounds in the presence of H_2O_2 (H_2O_2: 10a = 2:1). The reaction mixture was incubated at rt for 15 h in organic (DMSO) to aqueous solvent at 1:9 ratio.

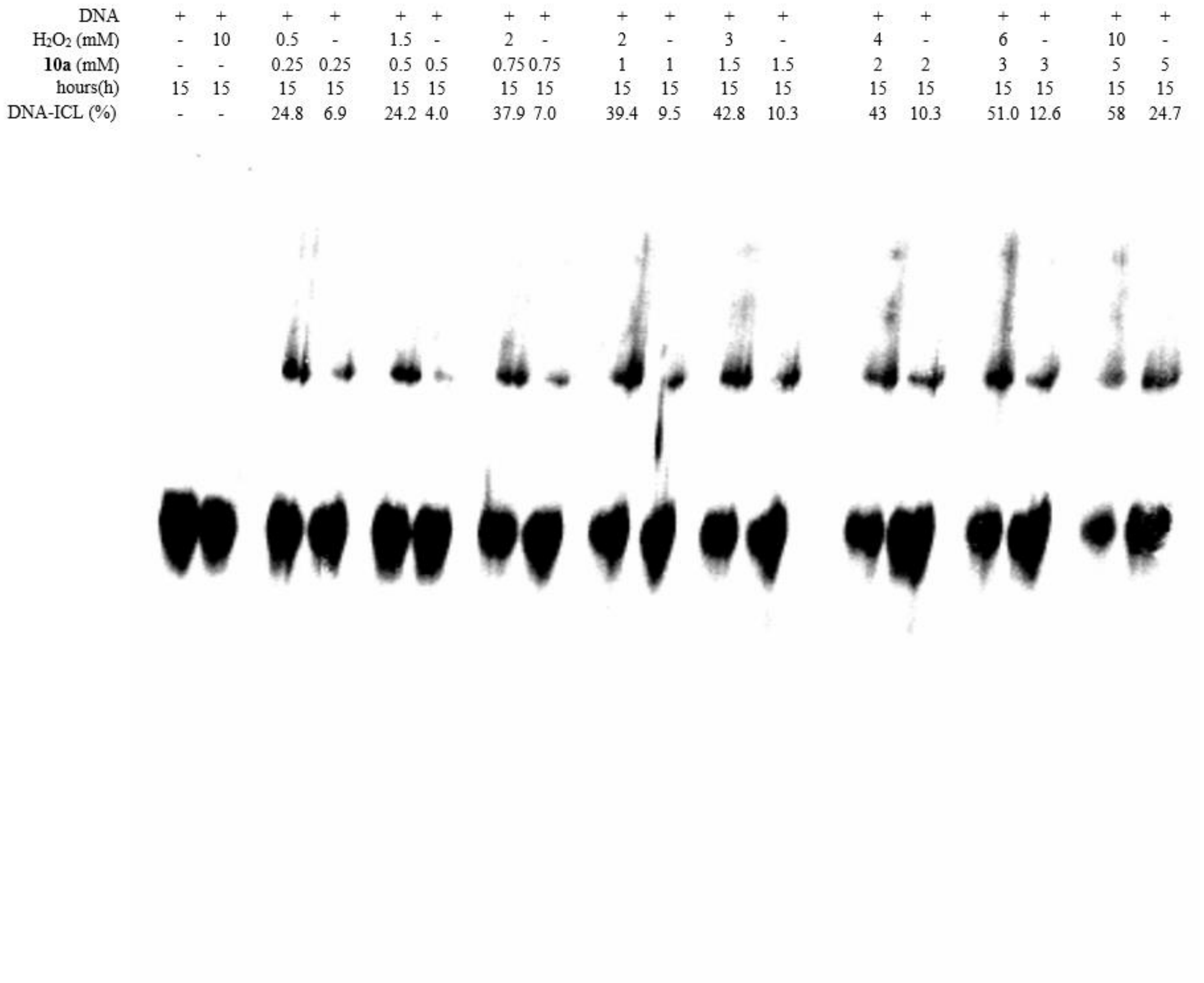

Figure S3. Trial 2 concentration dependent study of DNA cross-link formation induced by 10a upon H_2O_2-activation. Phosphorimage autoradiogram of 20% denaturing PAGE analysis of ICL formation with duplex DNA 11 and different concentration of compounds in the presence of H_2O_2 (H_2O_2: 10a = 2:1). The reaction mixture was incubated at rt for 15 h in organic (DMSO) to aqueous solvent at 1:9 ratio.

1.3 Time-dependent DNA-ICL formation

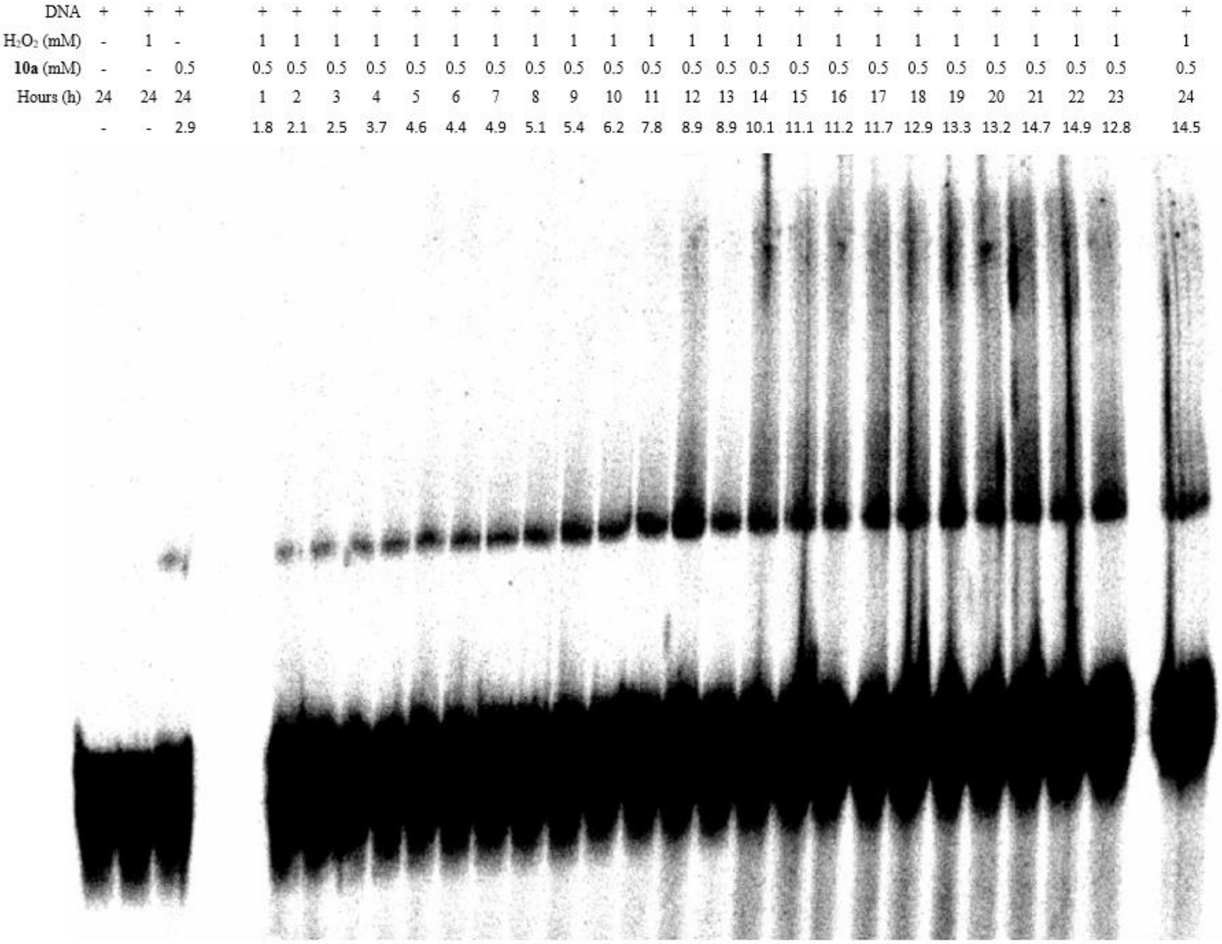

Figure S4. Time dependent study of DNA cross-link formation induced by 10a upon H_2O_2-activation at rt (Trial 1). Phosphorimage autoradiogram of 20% denaturing PAGE analysis of ICL formation. The reaction mixture of duplex DNA 11 (0.05µM), 10a (0.5 mM), and H_2O_2 (1.0 mM) was incubated at rt from 1 h to 24 h in organic (DMSO) to aqueous solvent at 1:9 ratio.

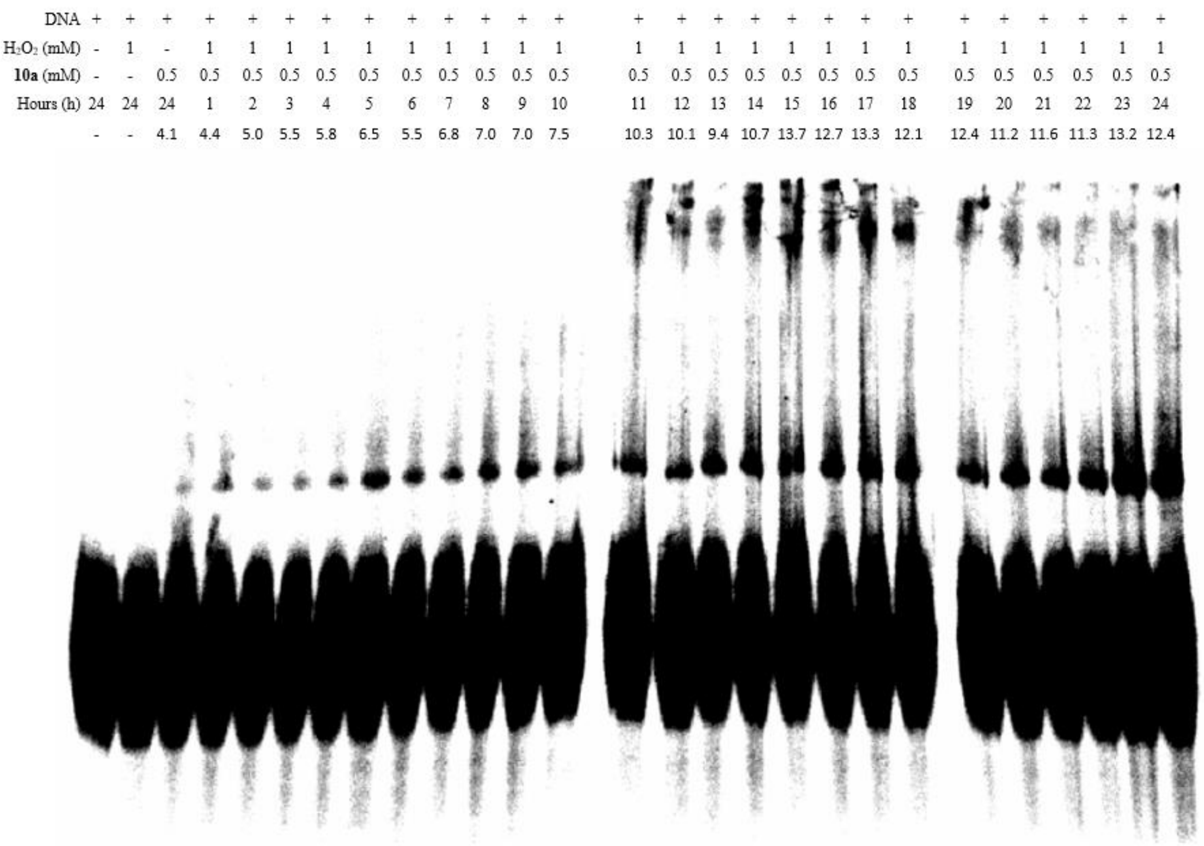

Figure S5. Time dependent study of DNA cross-link formation induced by 10a upon H_2O_2-activation at rt (Trial 2). Phosphorimage autoradiogram of 20% denaturing PAGE analysis of ICL formation. The reaction mixture of duplex DNA 11 (0.05μM), 10a (0.5 mM), and H_2O_2 (1.0 mM) was incubated at rt from 1 h to 24 h in organic (DMSO) to aqueous solvent at 1:9 ratio.

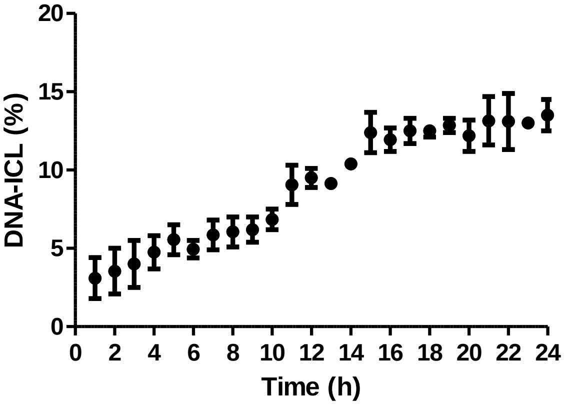

Figure S6. The time-dependence of ICL formation for 10a (0.5 mM) upon H_2O_2-activation (1 mM) at rt [the data are the average of two independent experiments (trial 1 in Figure S4 and trial 2 in Figure S5)].

2. Dose-dependent apoptosis induced by FAN-NM-CH_3

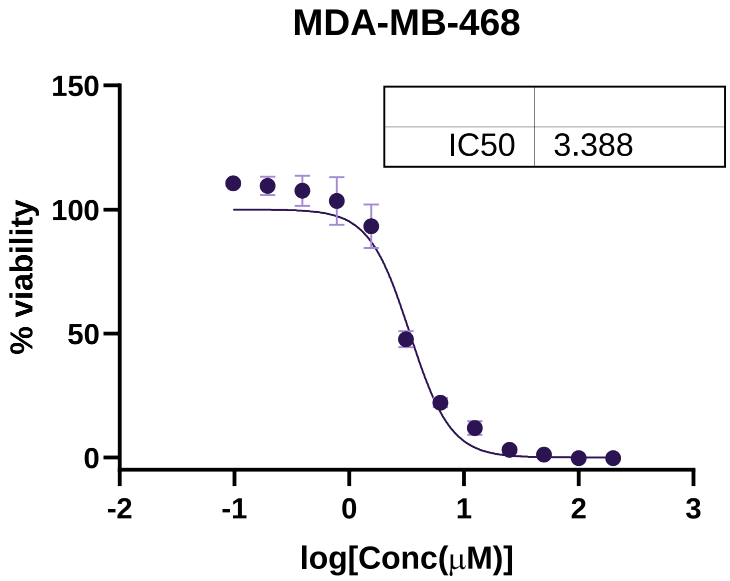

Figure S7. Dose-dependent apoptosis induced by **FAN-NM-CH_3** in MDA-MB-468 cancer cells. Cells were treated with **FAN-NM-CH_3** at concentrations ranging from 0 to 200 μM for 48 hours at 37 °C (n = 3).

3. In vivo determination of metabolites from Xenograft models

Table S1. *In vivo* metabolites of 10a and 10b and HPLC retention time (R_t).[a]

	0 – 24 h (R_t/min)		24 – 48 h (R_t/min)	
Phase II metabolite	urine	feces	urine	feces
glucoside	<LOD	<LOD	<LOD	<LOD
14a,b glucuronide	0.5 – 0.6	<LOD	<LOD	<LOD

[a] The method was developed with an ACQUITY CSH C18 column (2.1 mm × 50 mm, 1.7 µm particle size) using gradient: 0 – 8.0 min 10%-40% MeCN in A, 8.0-9.0 min 40% MeCN in A, 9.0-12.0 min 60% MeCN in A, 12.0-16.0 min 80% MeCN in A, 16.0-17.0 min 80% MeCN in A, 17.0-19.0 min 100% MeCN in A, 19.0-20.0 min 100% MeCN in A, 20.0-21.0 min 10% MeCN in A, 12.0-13.0 min 100%-5% MeCN in A, 13.0-15.0 min 5% MeCN in A at a flow rate of 0.5 mL/min (solution A: 5 mM ammonium acetate in water, solution B: 0.1% FA in MeCN).

4. HRMS of the metabolites detected in MDA-MB-468 cell culture.

[illegible]

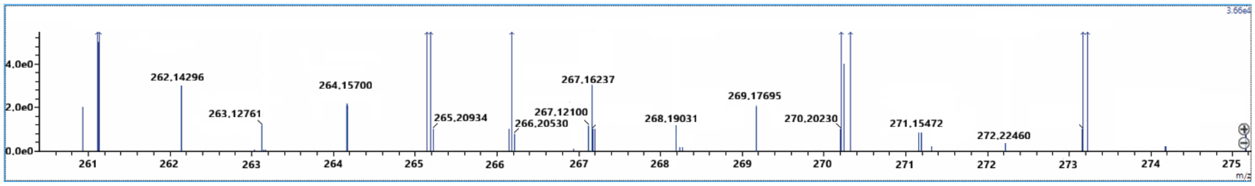

[illegible]

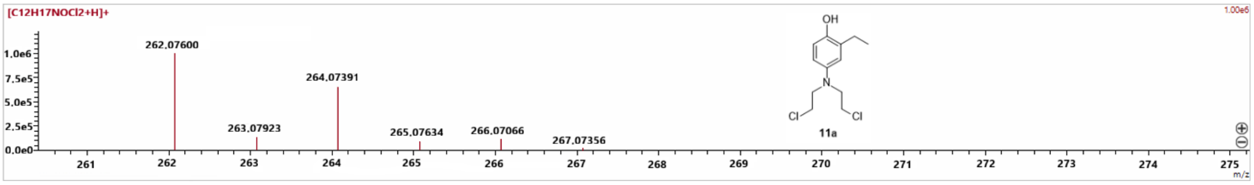

[illegible]

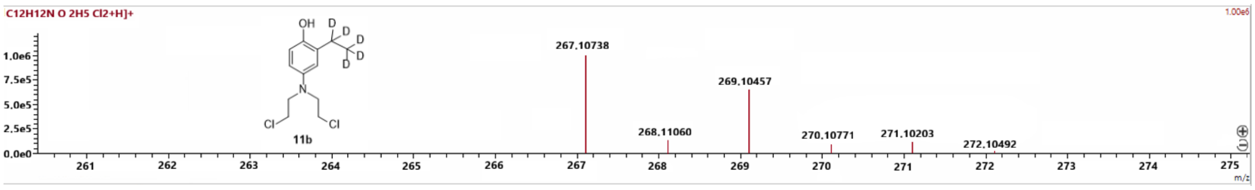

Figure S8. High-resolution mass spectra of metabolites detected in MDA-MB-468 cell culture following incubation with an equimolar mixture of prodrugs **10a** and **10b** (100 µM). The samples were collected over 6 – 24 h incubation and analyzed by HRMS Q-TOF-MS (ESI).

Q-TOF-MS (ESI) of **11a**, HRMS-ESI (+) (*m/z*): $[M+H]^+$ calcd. for $C_{12}H_{18}Cl_2NO^+$ 262.07600; found 262.14296.

Q-TOF-MS (ESI) of **11b**, HRMS-ESI (+) (*m/z*): $[M+H]^+$ calcd. for $C_{12}H_{13}D_5Cl_2NO^+$ 267.10738; found 267.12100.

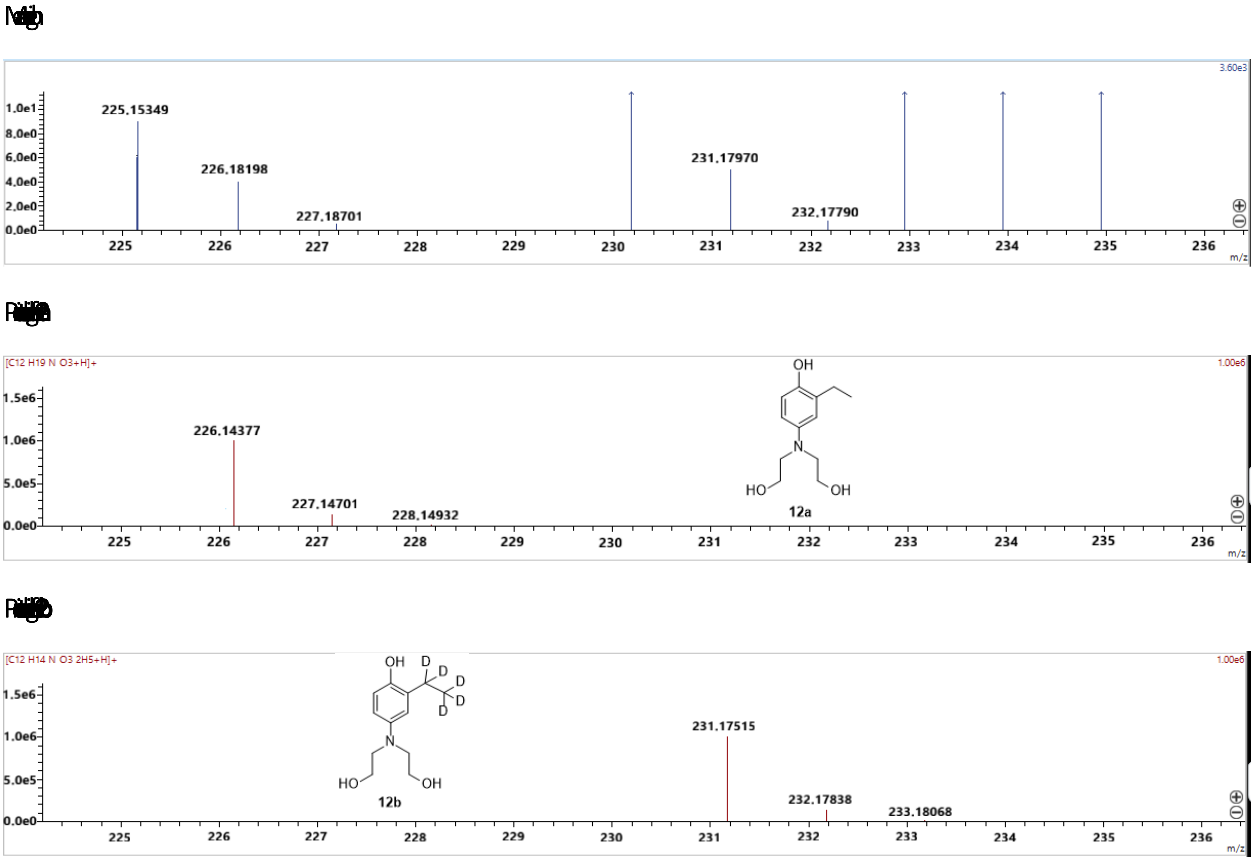

Figure S9. High-resolution mass spectra of metabolites detected in MDA-MB-468 cell culture following incubation with an equimolar mixture of prodrugs **10a** and **10b** (100 µM). The samples were collected over 6 – 24 h incubation and analyzed by HRMS Q-TOF-MS (ESI).

Q-TOF-MS (ESI) of **12a,** HRMS-ESI (+) (*m/z*): $[M+H]^+$ calcd. for $C_{12}H_{20}NO_3^+$ 226.14377; found 226.18198.

Q-TOF-MS (ESI) of **12b,** HRMS-ESI (+) (*m/z*): $[M+H]^+$ calcd. for $C_{12}H_{15}D_5NO_3^+$ 231.17515; found 231.17970.

5. **HRMS Spectra of a mixture of 10a and 10b**

Measured region

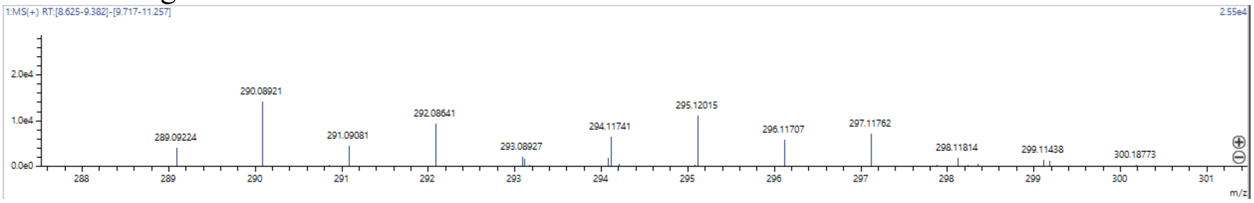

Predicted region

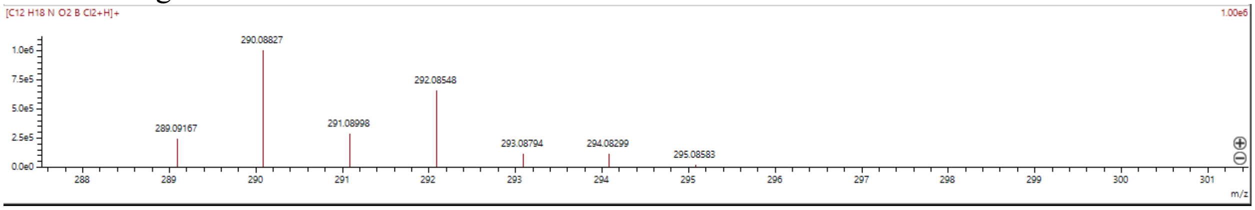

Predicted region

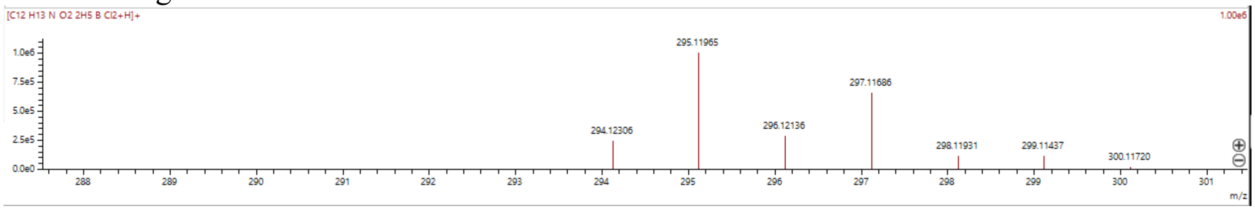

Figure S10. Q-TOF-MS (ESI) analysis of a 1:1 ratio mixture of 10a and 10b.

compound **10a**

HRMS-ESI (+) (*m/z*): $[M+H]^+$ calcd. for $C_{12}H_{19}BCl_2NO_2^+$ 290.08804; found: 290.08921.

compound **10b**

HRMS-ESI (+) (*m/z*): $[M+H]^+$ calcd. for $C_{12}H_{14}D_5BCl_2NO_2^+$ 295.11942; found: 295.12015.

6. NMR Spectra of synthesized molecules

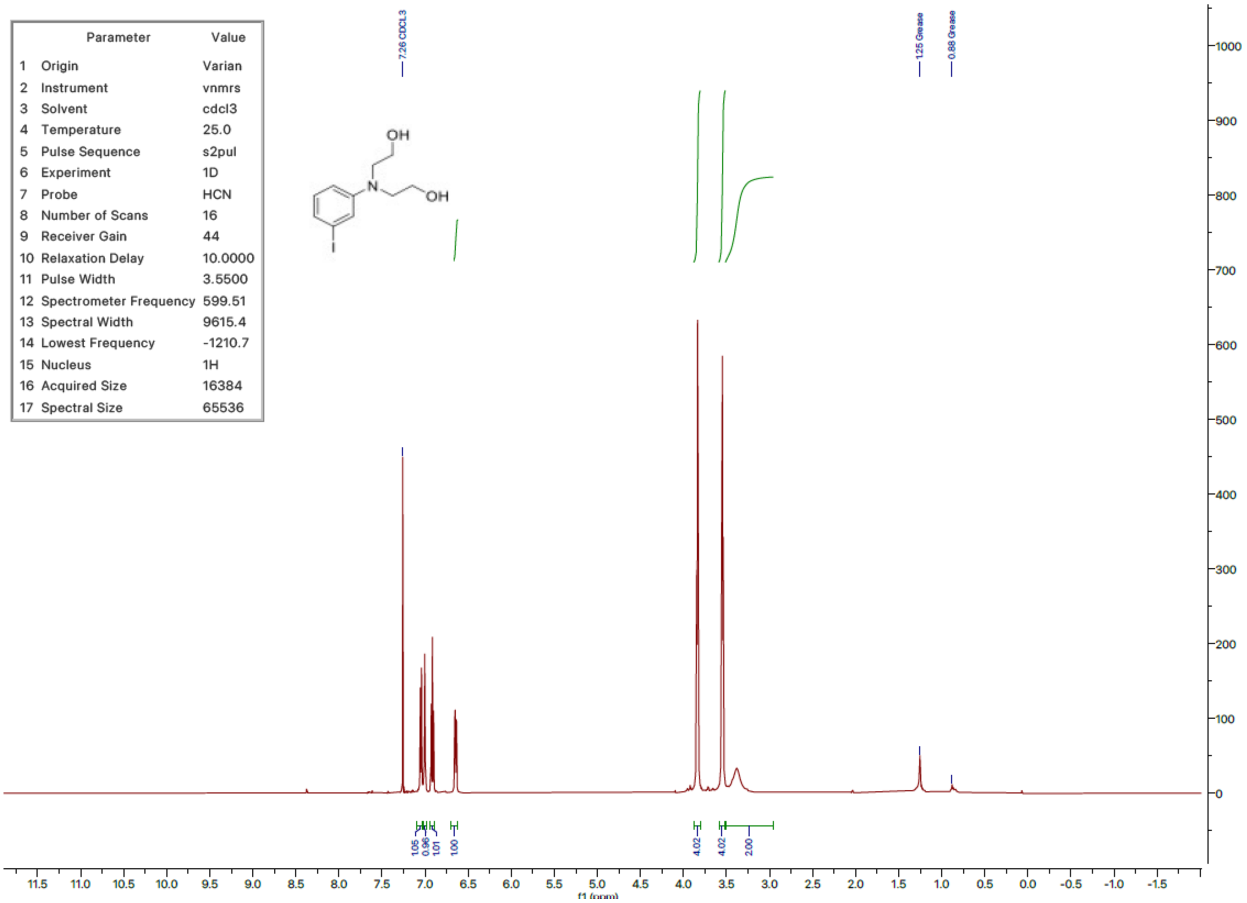

Figure S11a. ^{1}H NMR of 2 in $CDCl_3$.

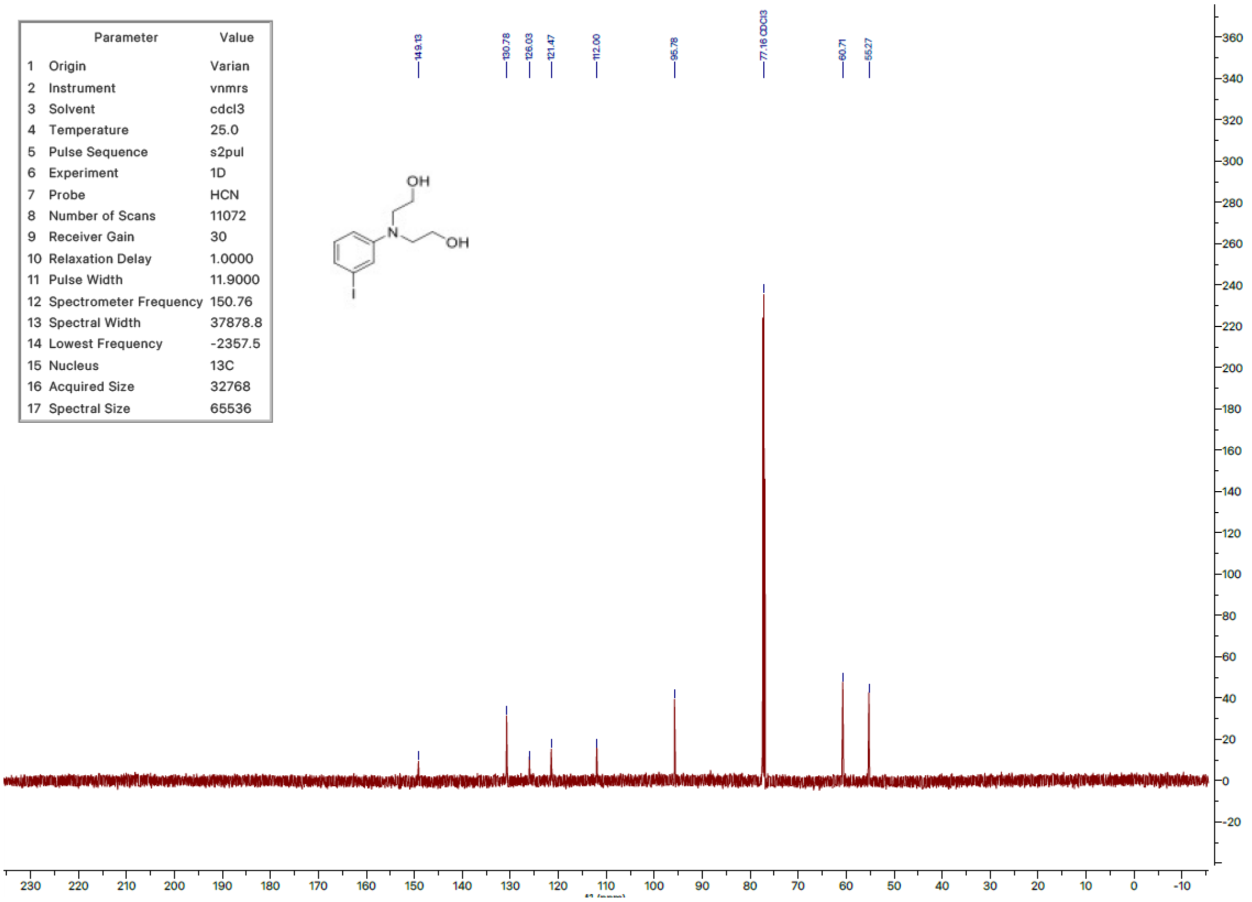

Figure S4. ^{13}C NMR of 2 in $CDCl_3$.

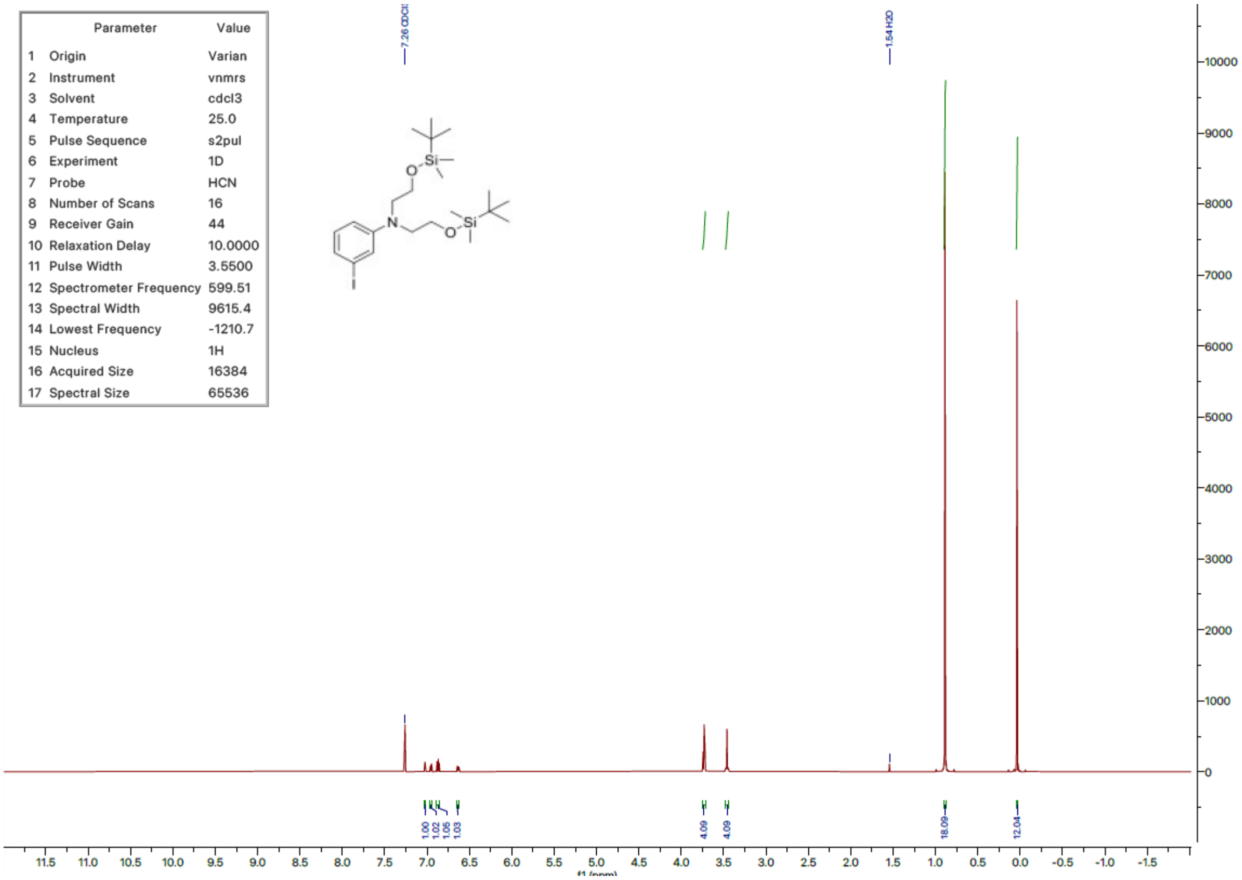

Figure S5. ^{1}H NMR of 3 in $CDCl_3$.

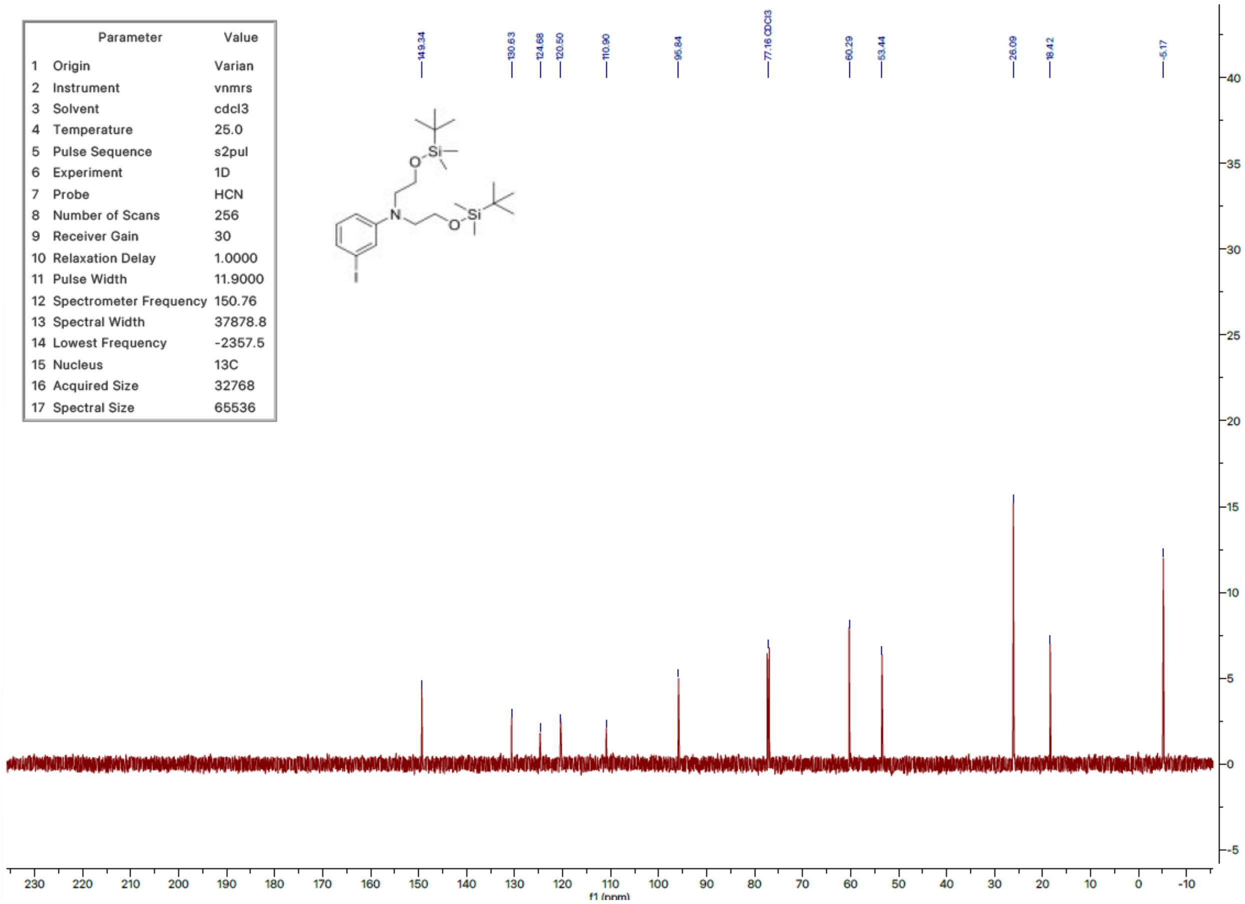

Figure S6b. ^{13}C NMR of 3 in $CDCl_3$.

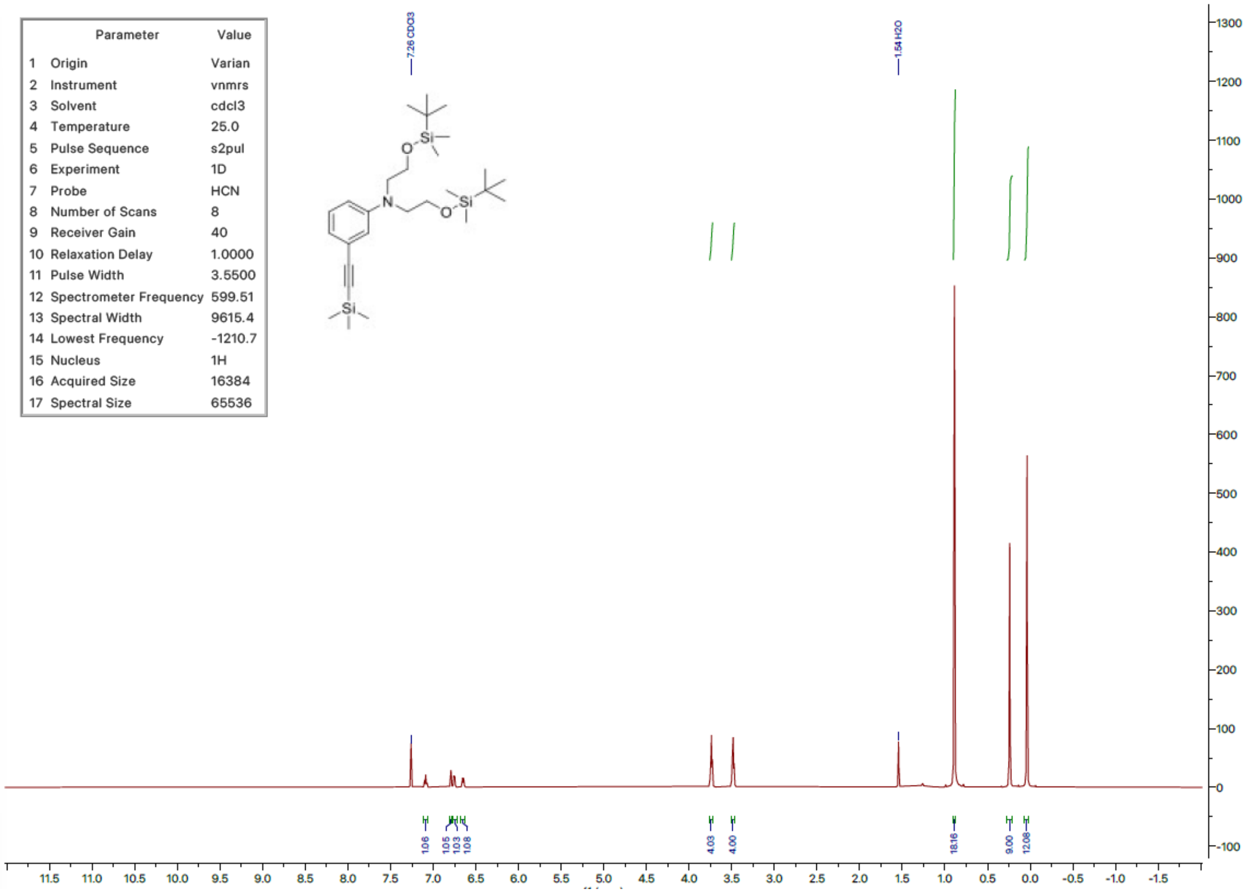

Figure S7. ^{1}H NMR of 4 in $CDCl_3$.

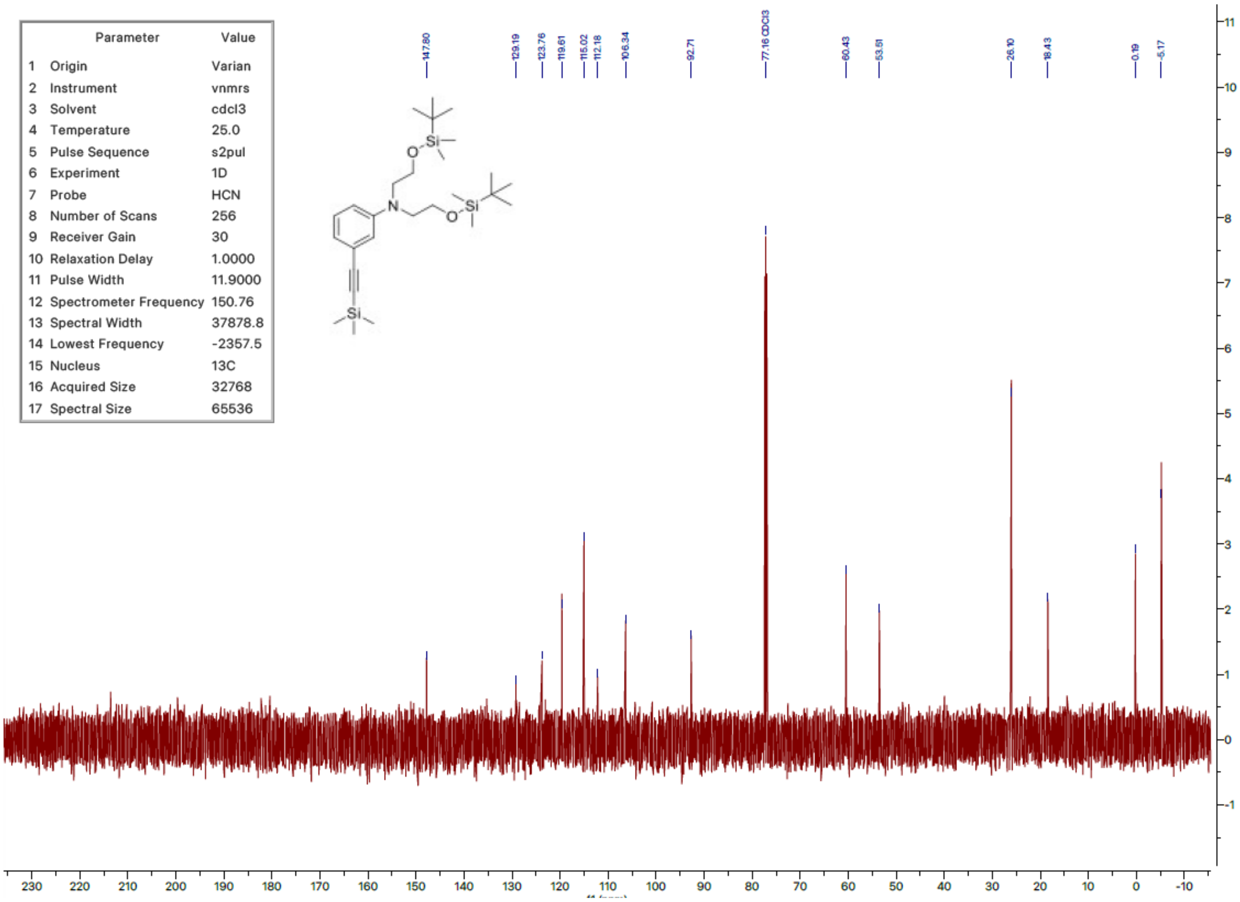

Figure S14. ^{13}C NMR of 4 in $CDCl_3$.

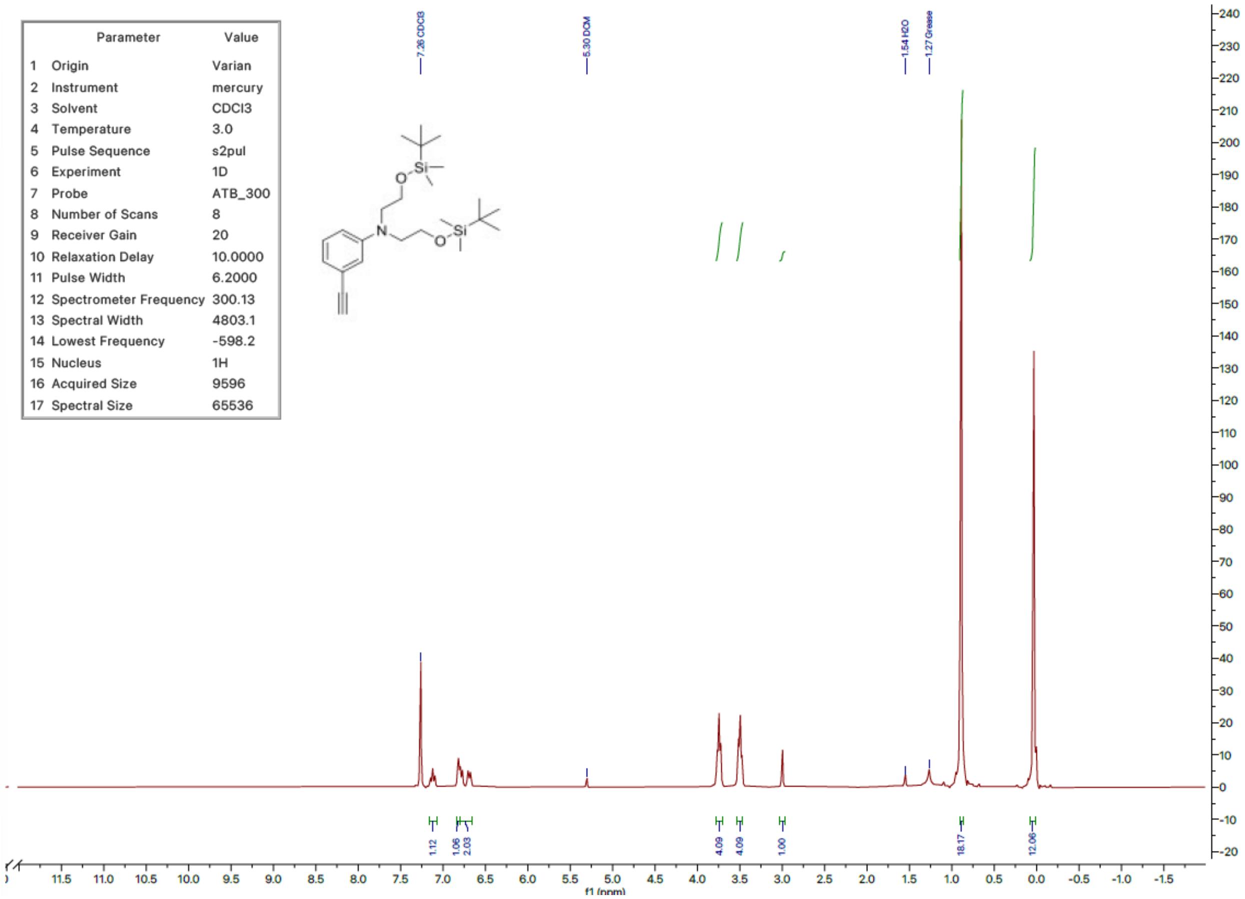

Figure S15. ^{1}H NMR of 5a in $CDCl_3$.

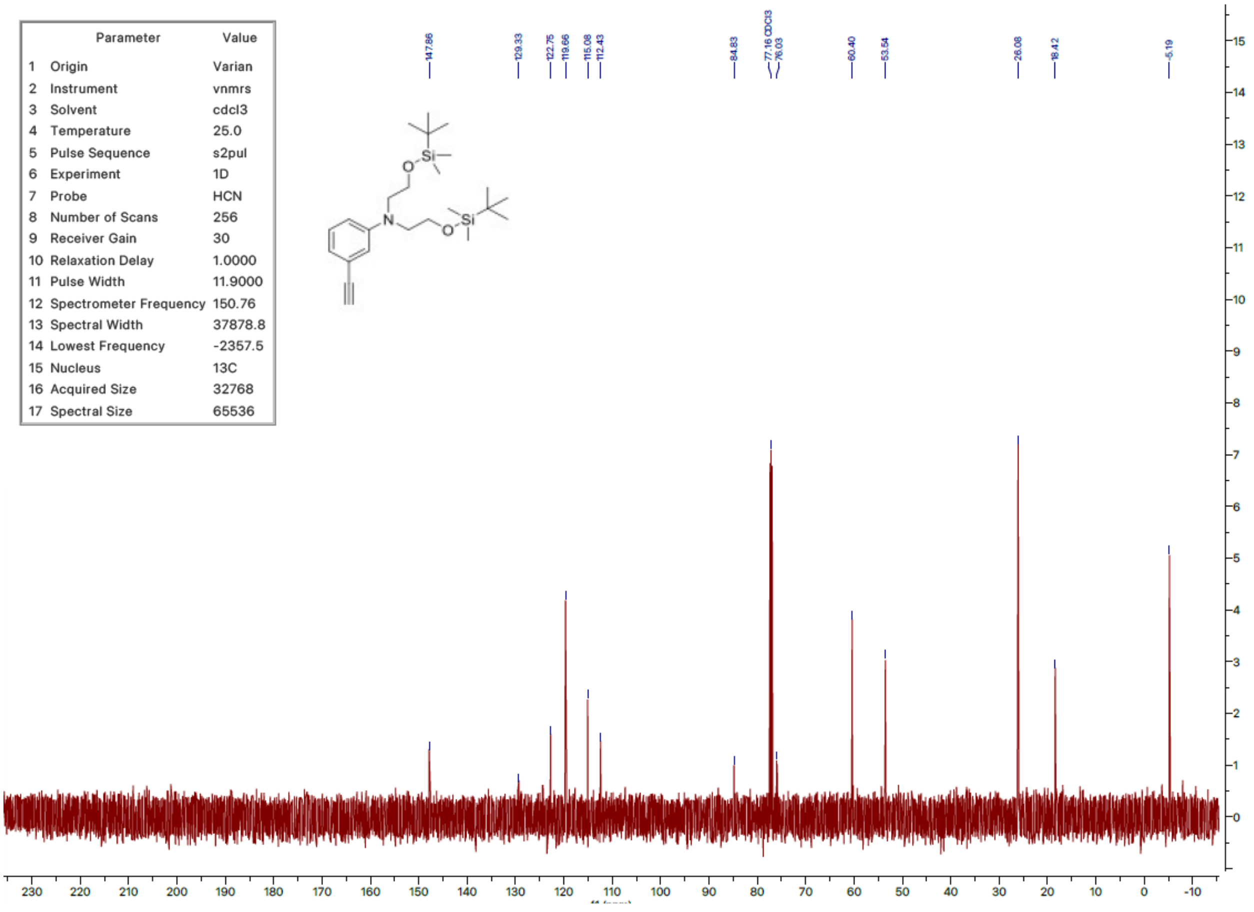

Figure S16. ^{13}C NMR of 5a in $CDCl_3$

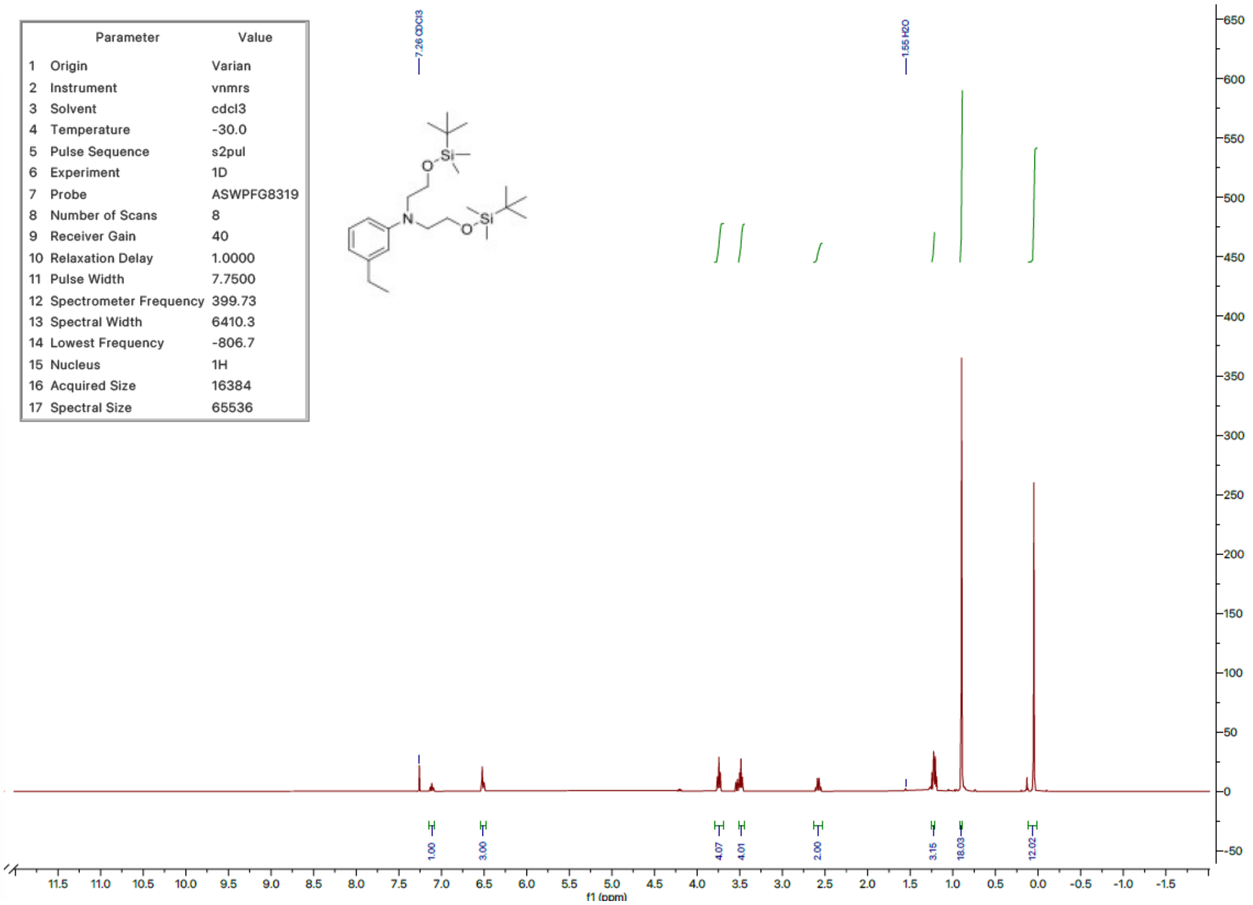

Figure S17. ^{1}H NMR of 6a in $CDCl_3$

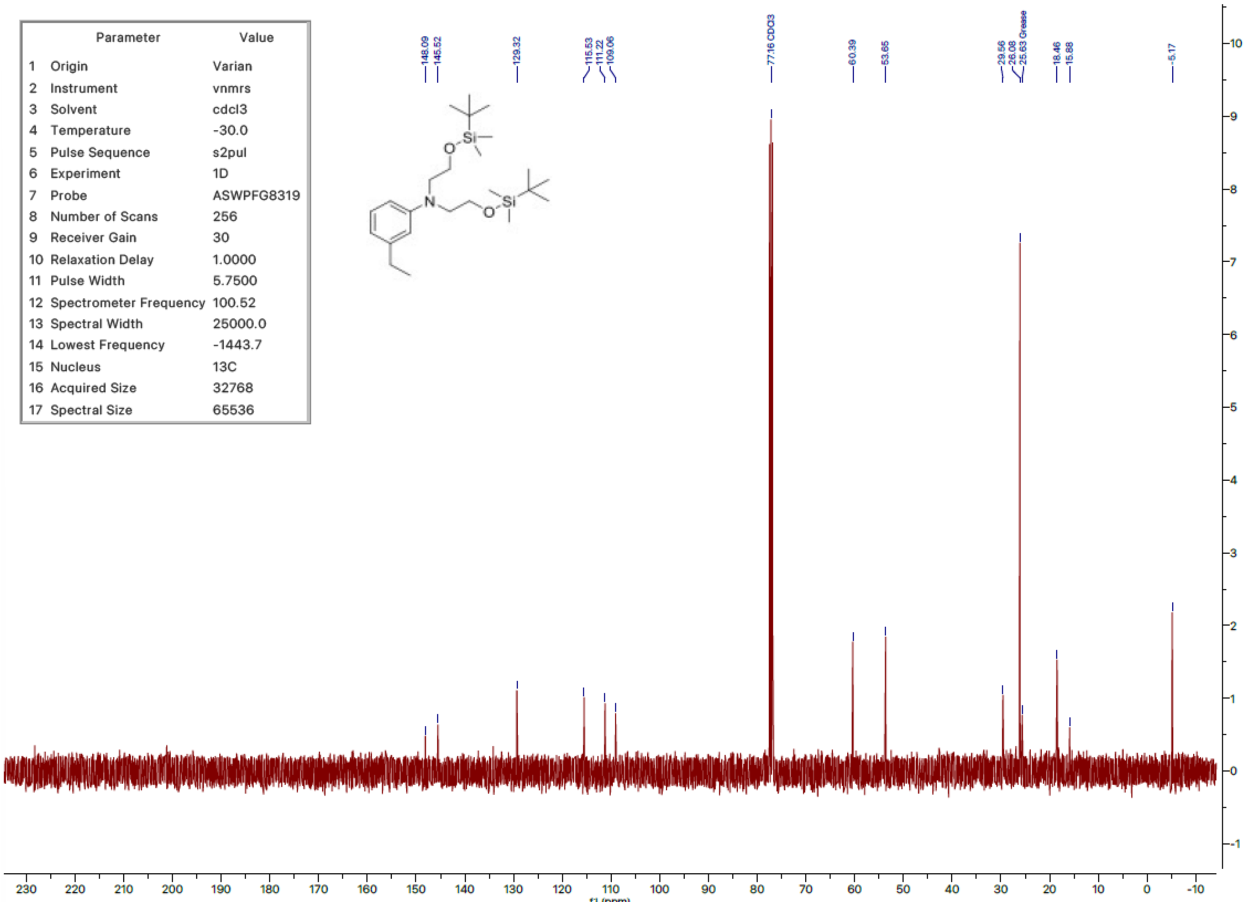

Figure S18. ^{13}C NMR of 6a in $CDCl_3$

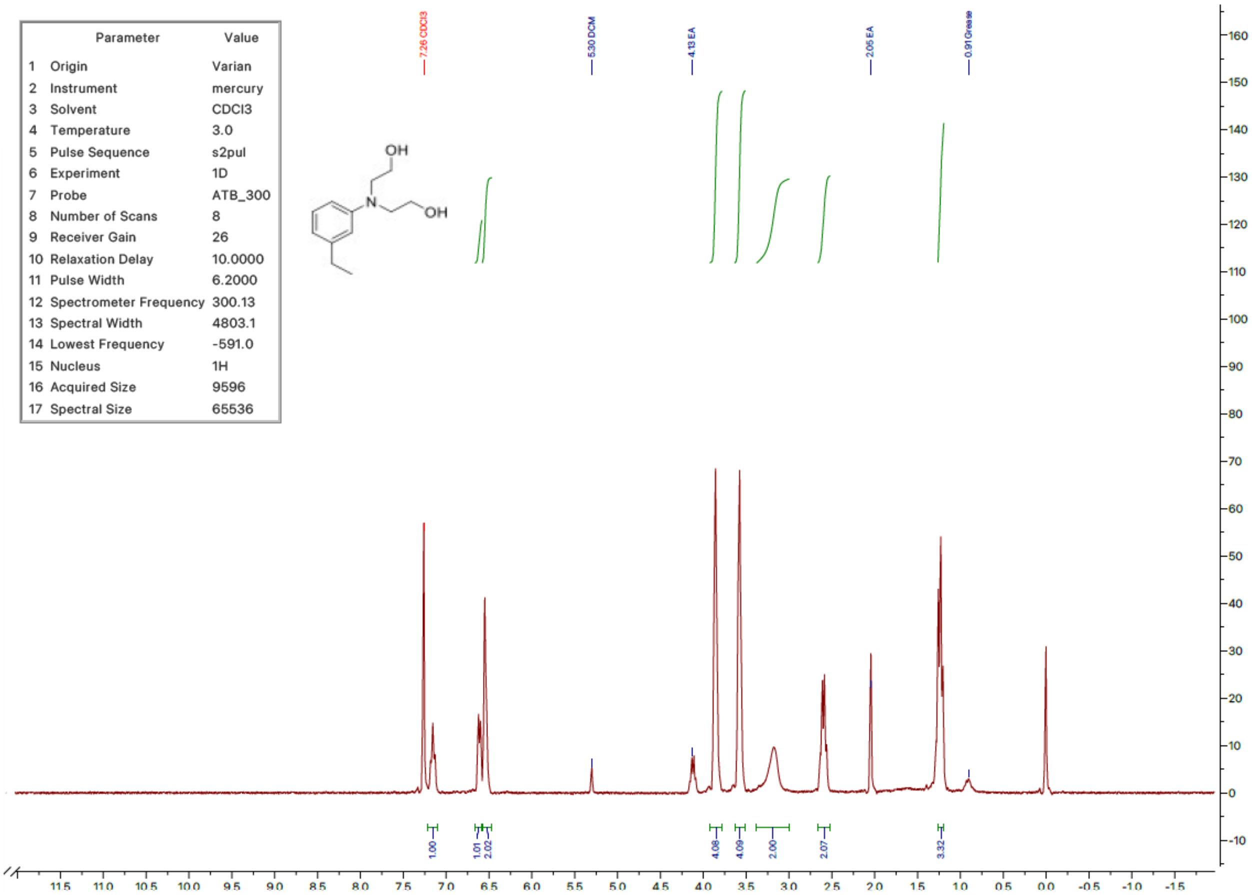

Figure S19. ^{1}H NMR of 7a in $CDCl_3$

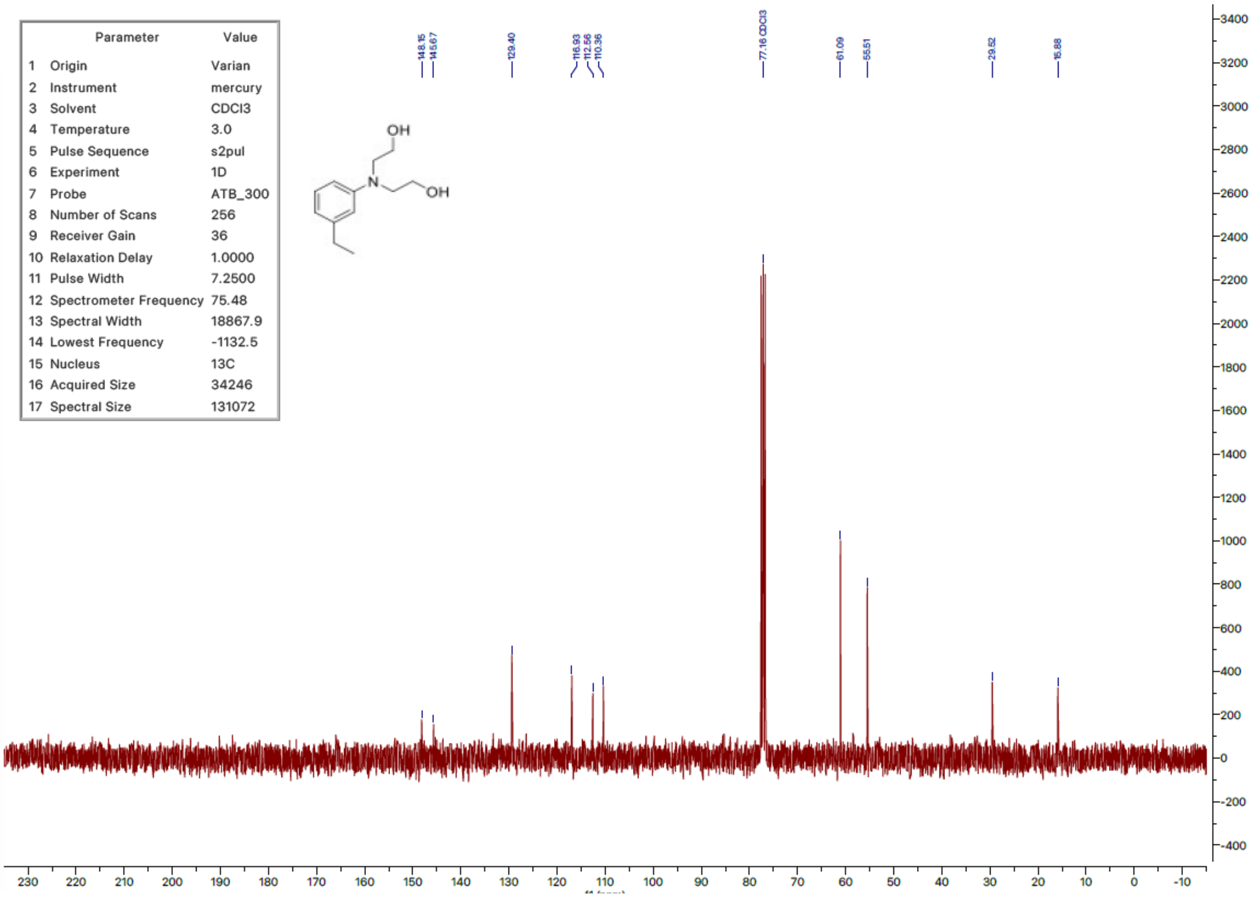

Figure S8. ^{13}C NMR of 7a in $CDCl_3$

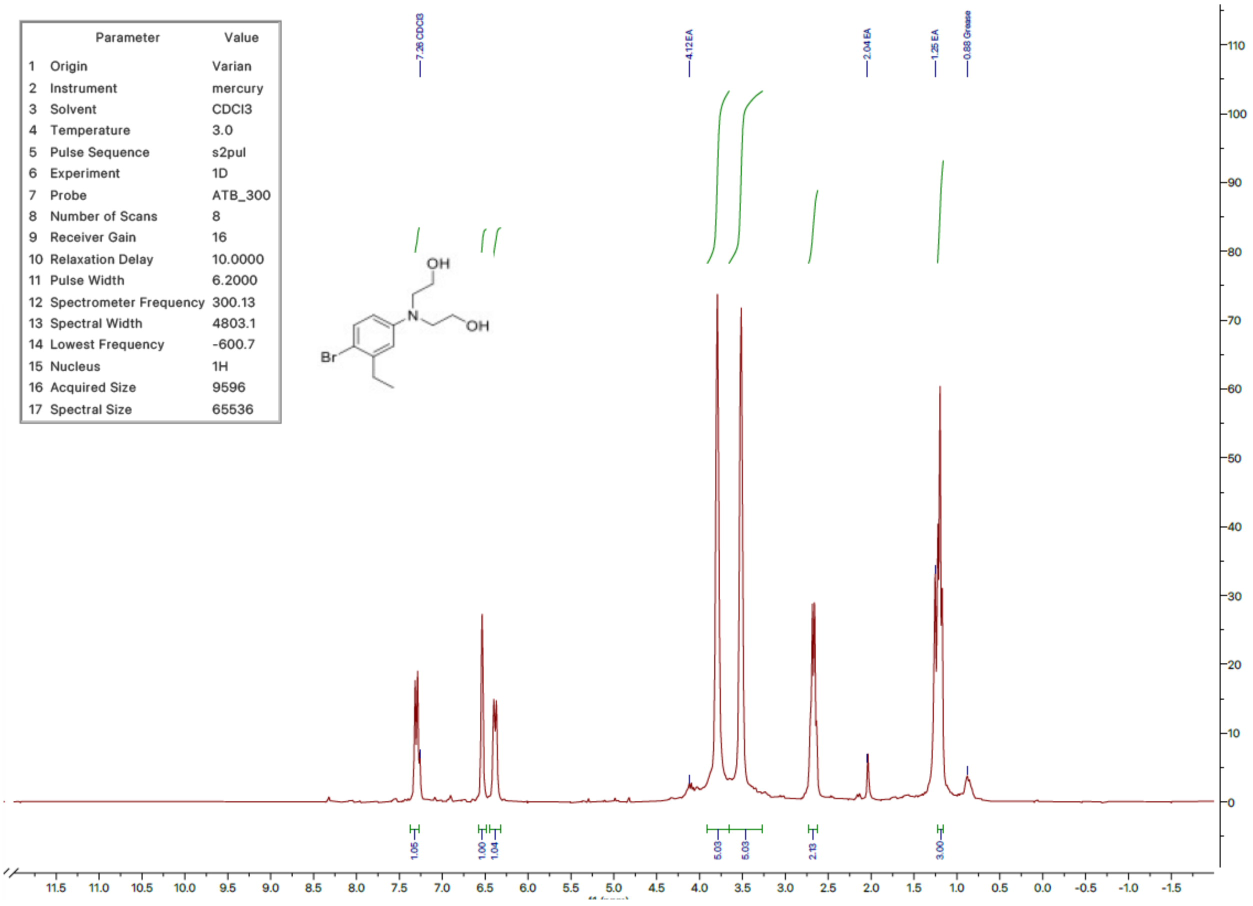

Figure S9. ^{1}H NMR of 8a in $CDCl_3$

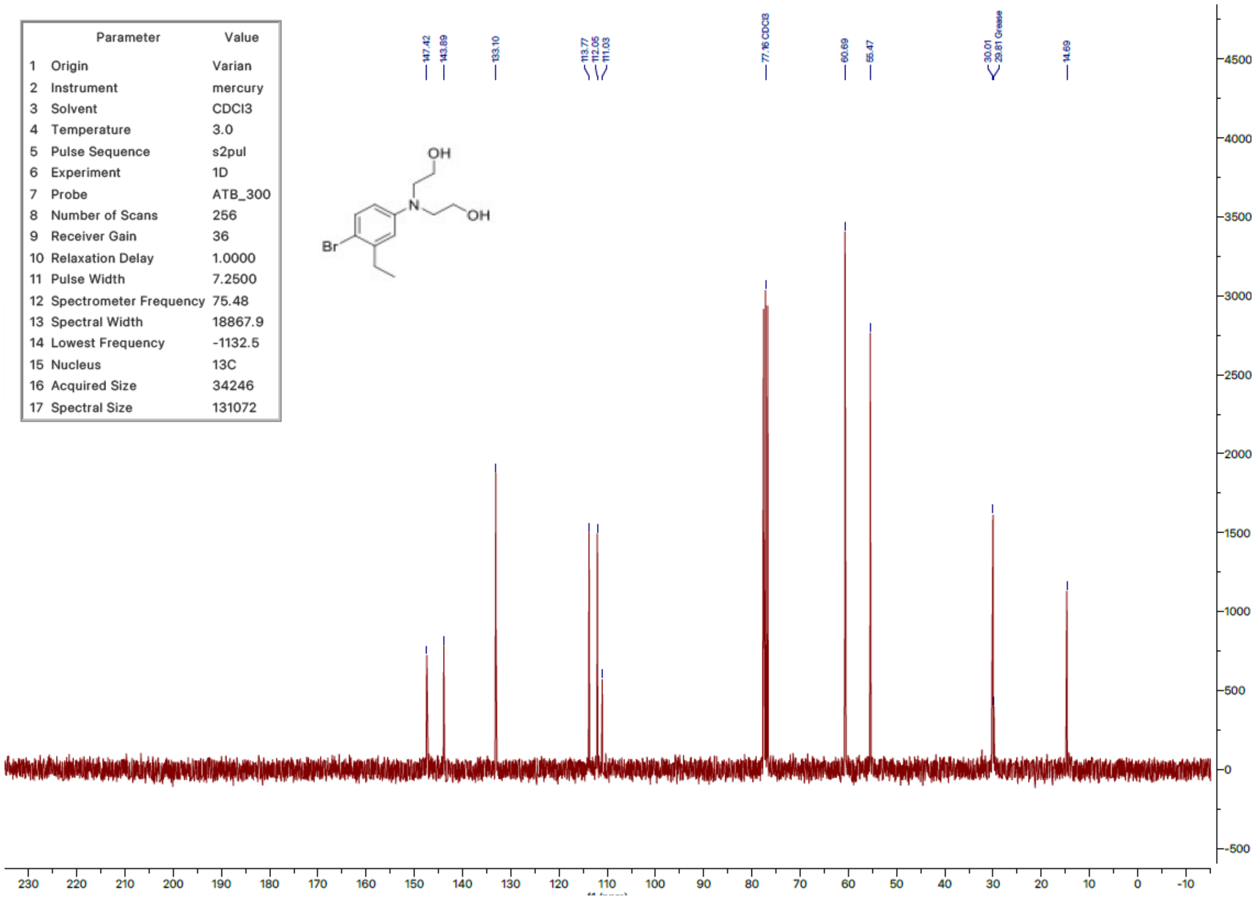

Figure S10. ^{13}C NMR of 8a in $CDCl_3$

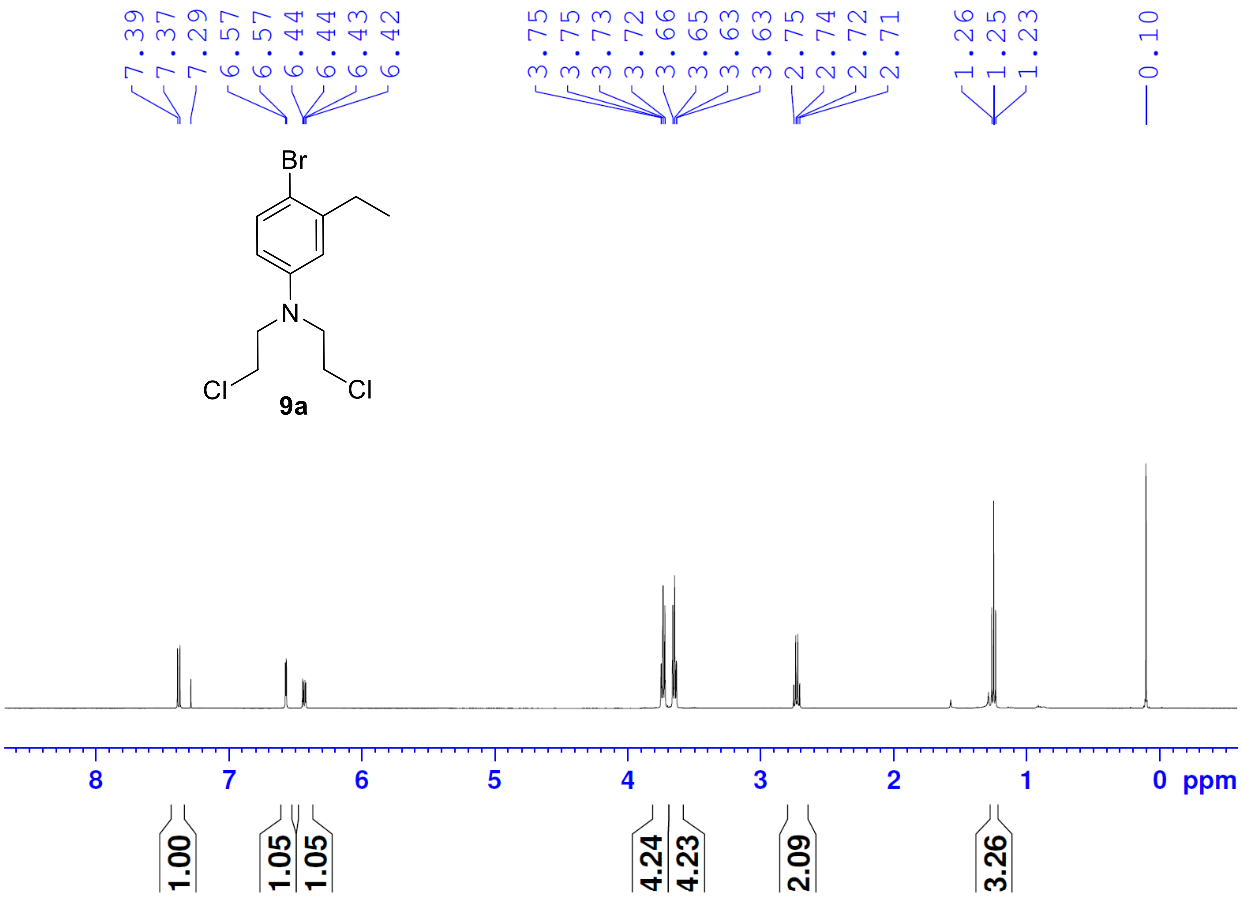

Figure S11. ^{1}H NMR in $CDCl_3$

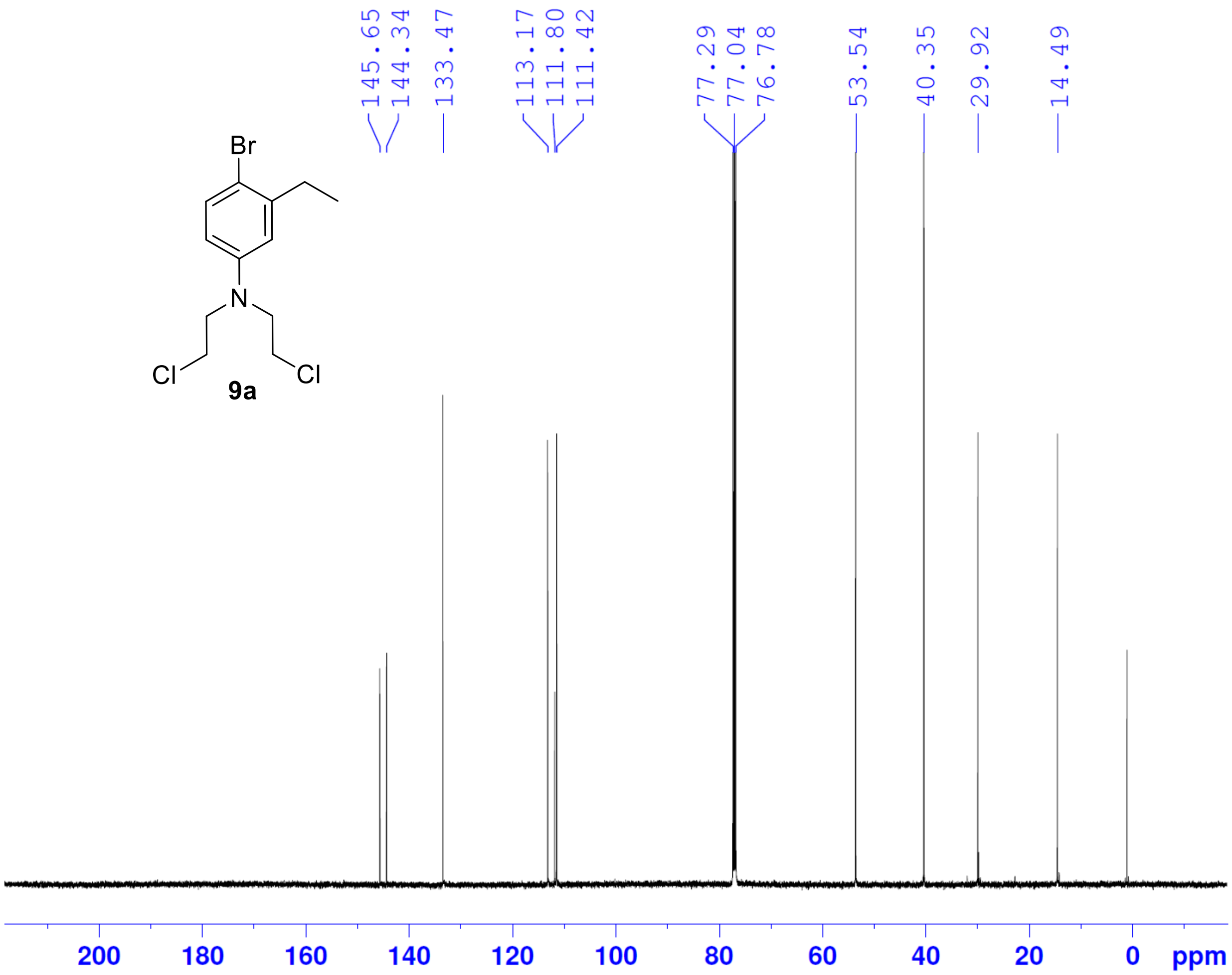

Figure S24. ^{13}C NMR in $CDCl_3$.

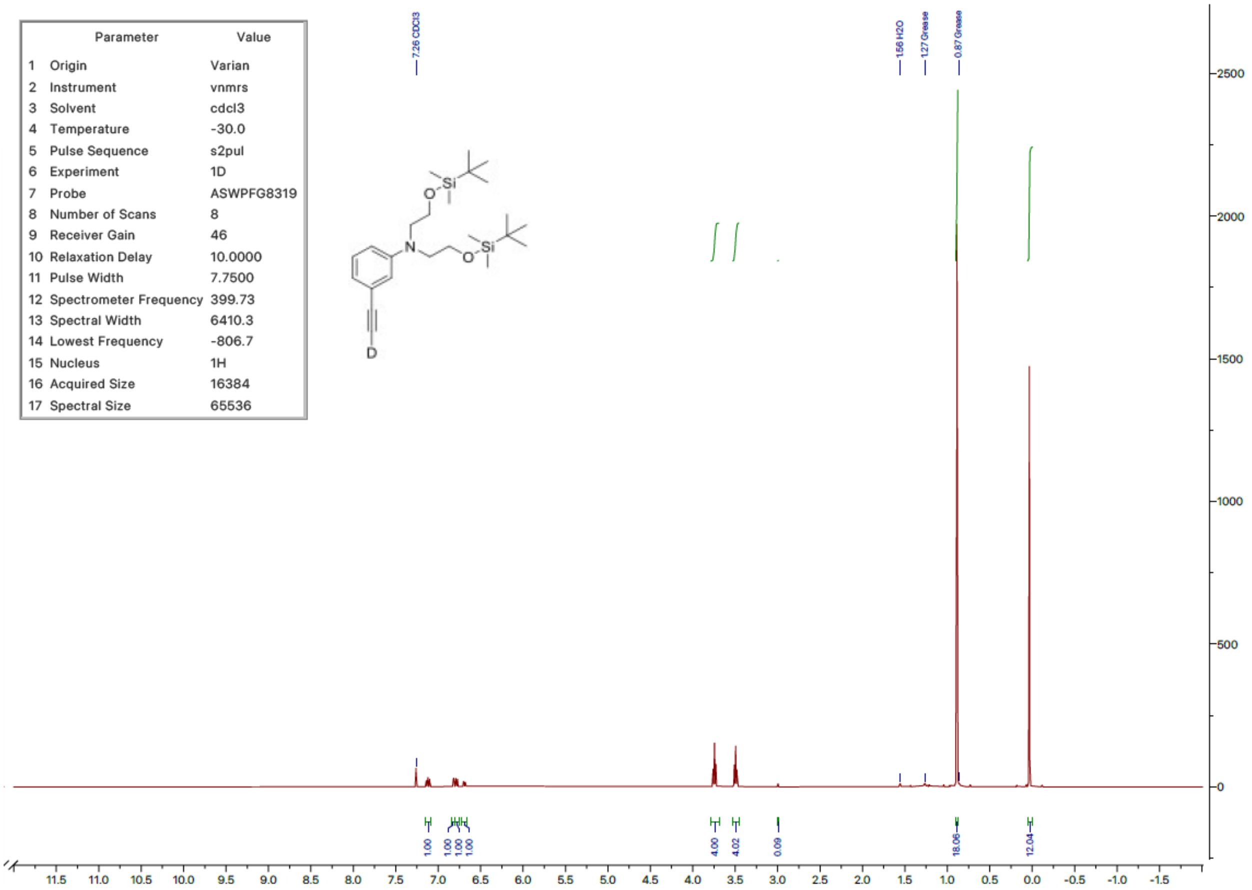

Figure S25. ^{1}H NMR of 5b in $CDCl_3$

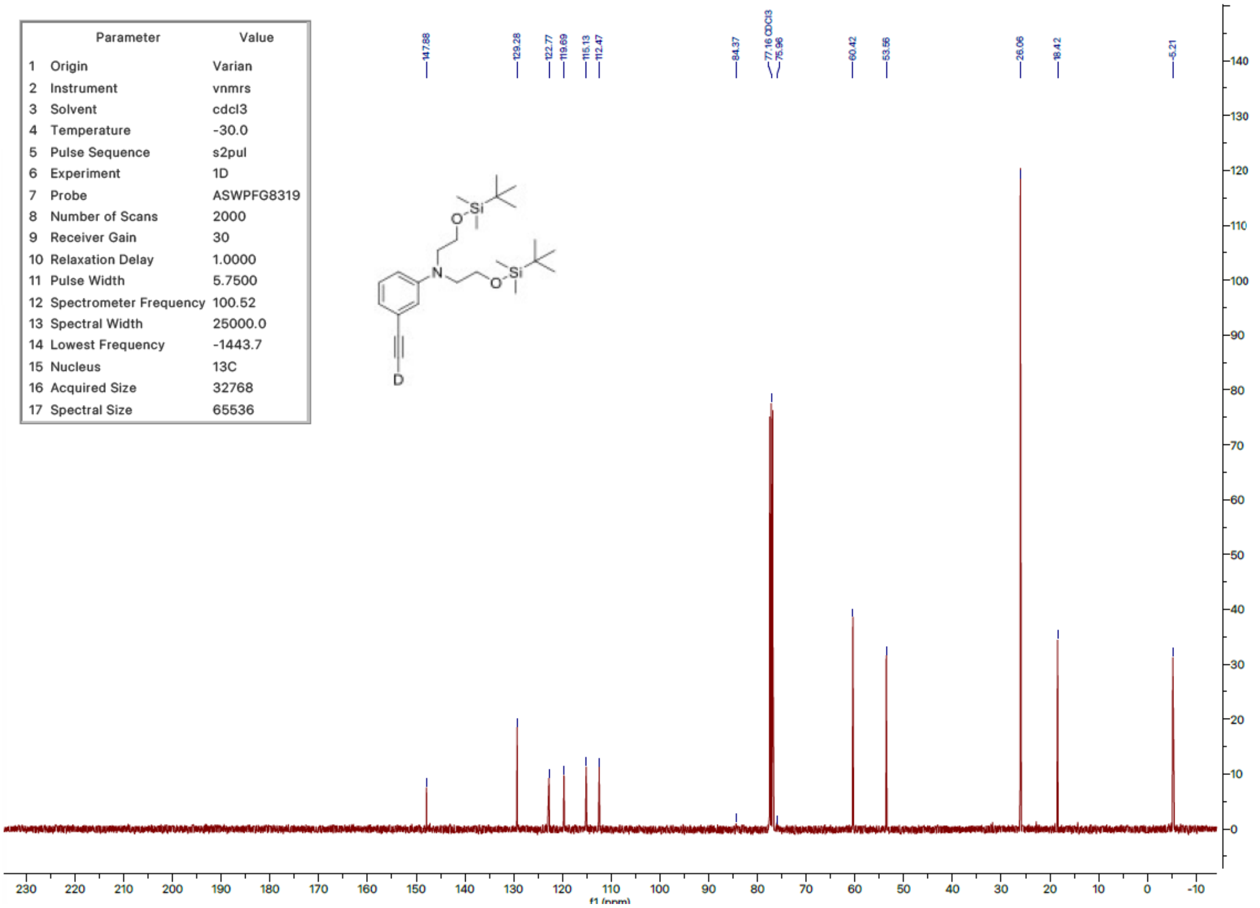

Figure S26. ^{13}C NMR of 5b in $CDCl_3$

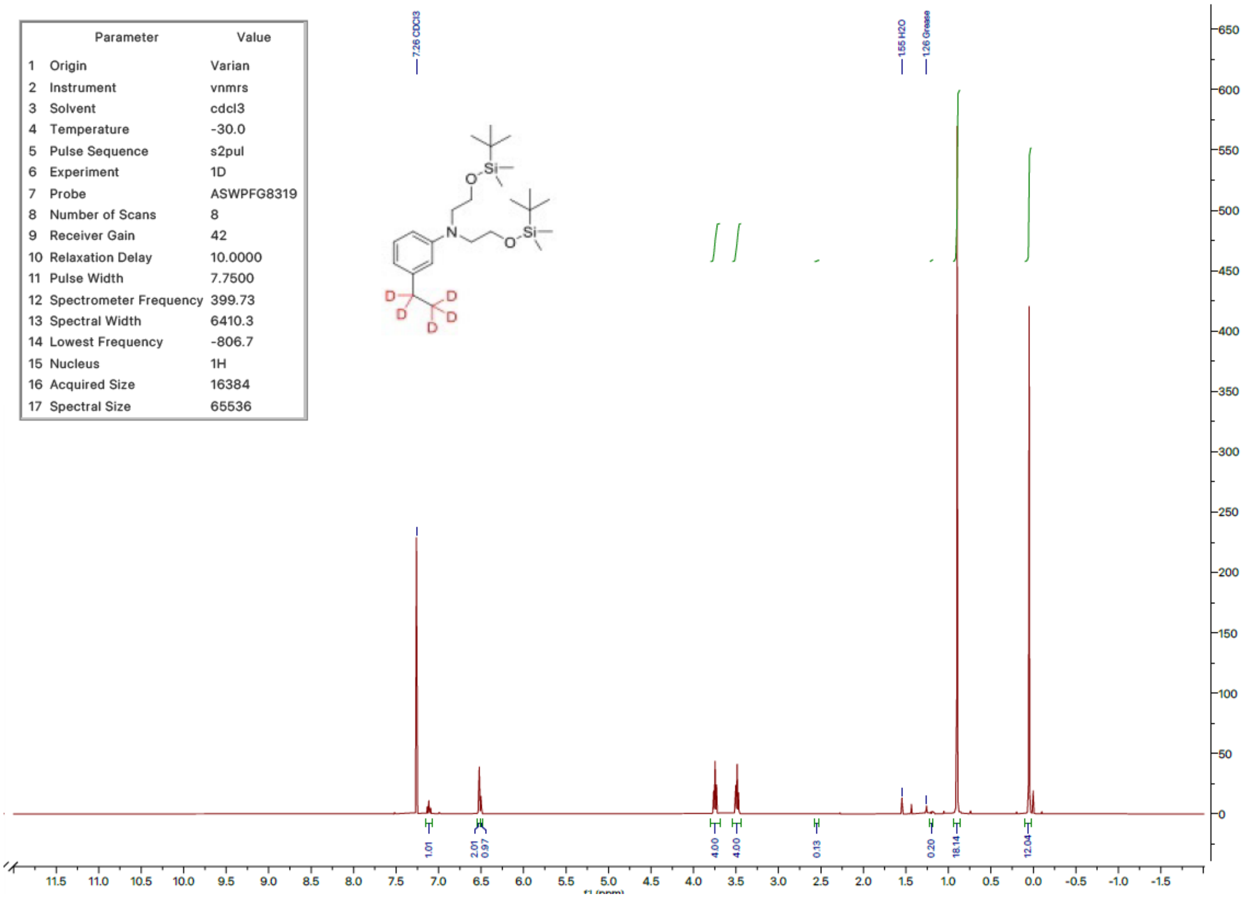

Figure S27. ^{1}H NMR of 6b in $CDCl_3$

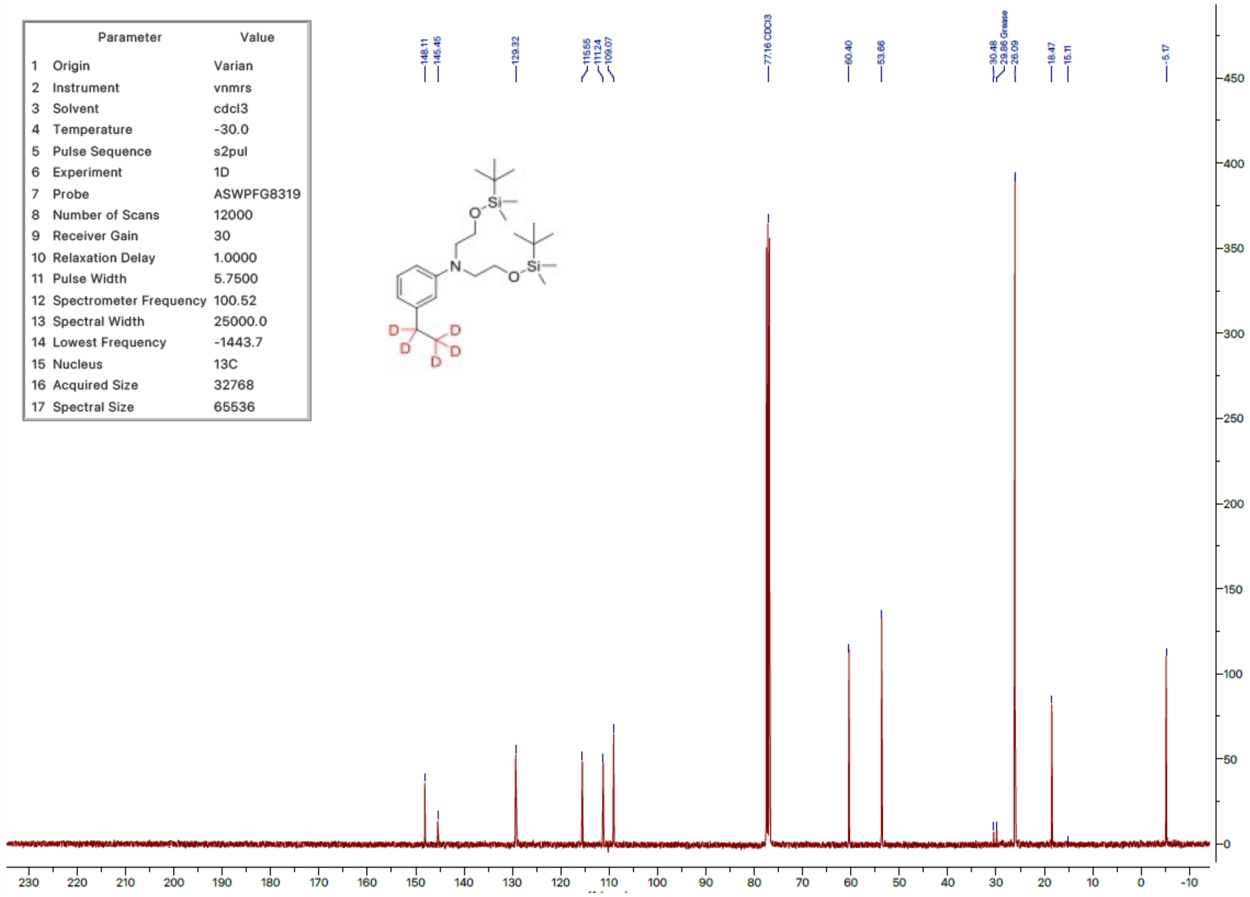

Figure S28. ^{13}C NMR of 6b in $CDCl_3$

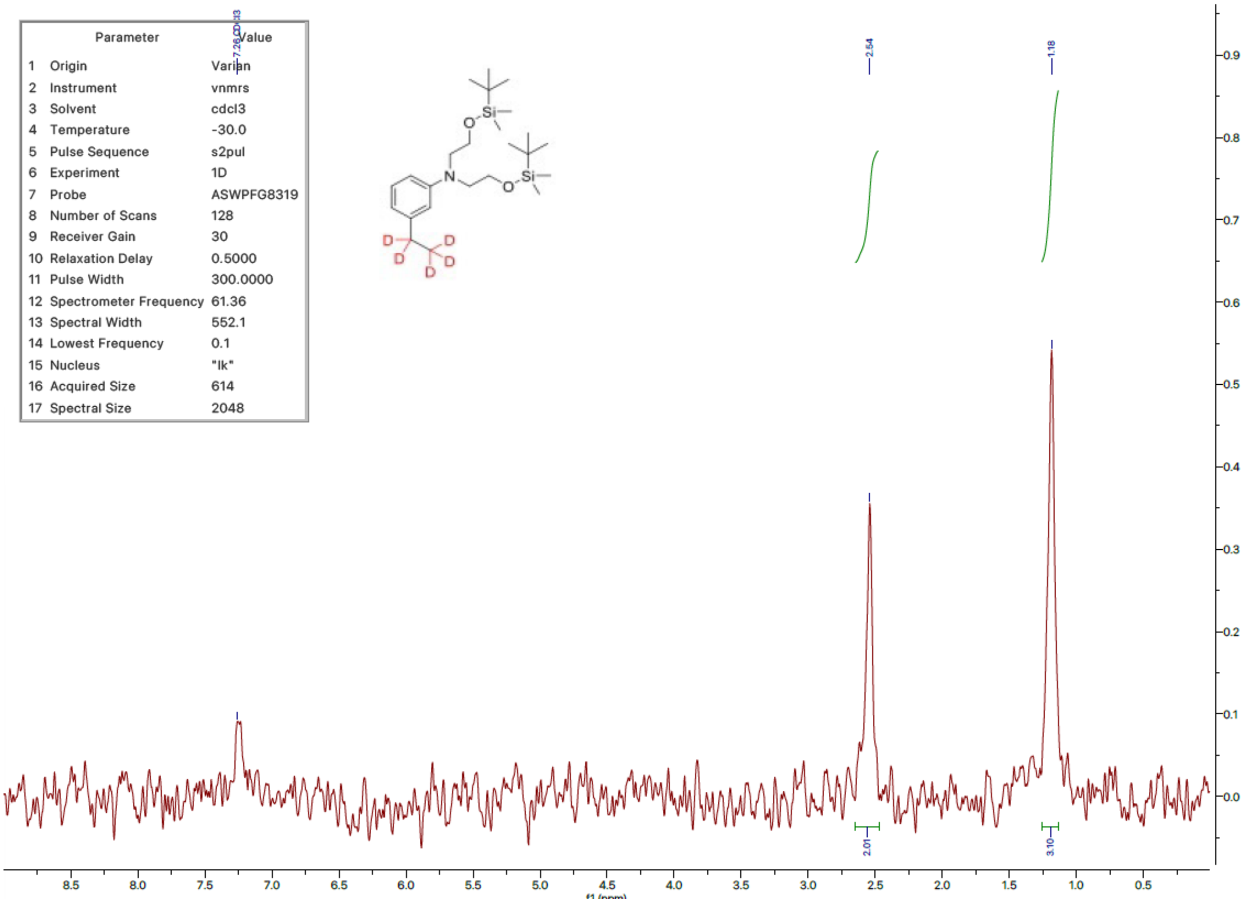

	Parameter	Value
1	Origin	Varian
2	Instrument	vnmrs
3	Solvent	cdcl3
4	Temperature	-30.0
5	Pulse Sequence	s2pul
6	Experiment	1D
7	Probe	ASWPFG8319
8	Number of Scans	128
9	Receiver Gain	30
10	Relaxation Delay	0.5000
11	Pulse Width	300.0000
12	Spectrometer Frequency	61.36
13	Spectral Width	552.1
14	Lowest Frequency	0.1
15	Nucleus	"lk"
16	Acquired Size	614
17	Spectral Size	2048

Figure S29. ^{1}H NMR of 6b in $CDCl_3$

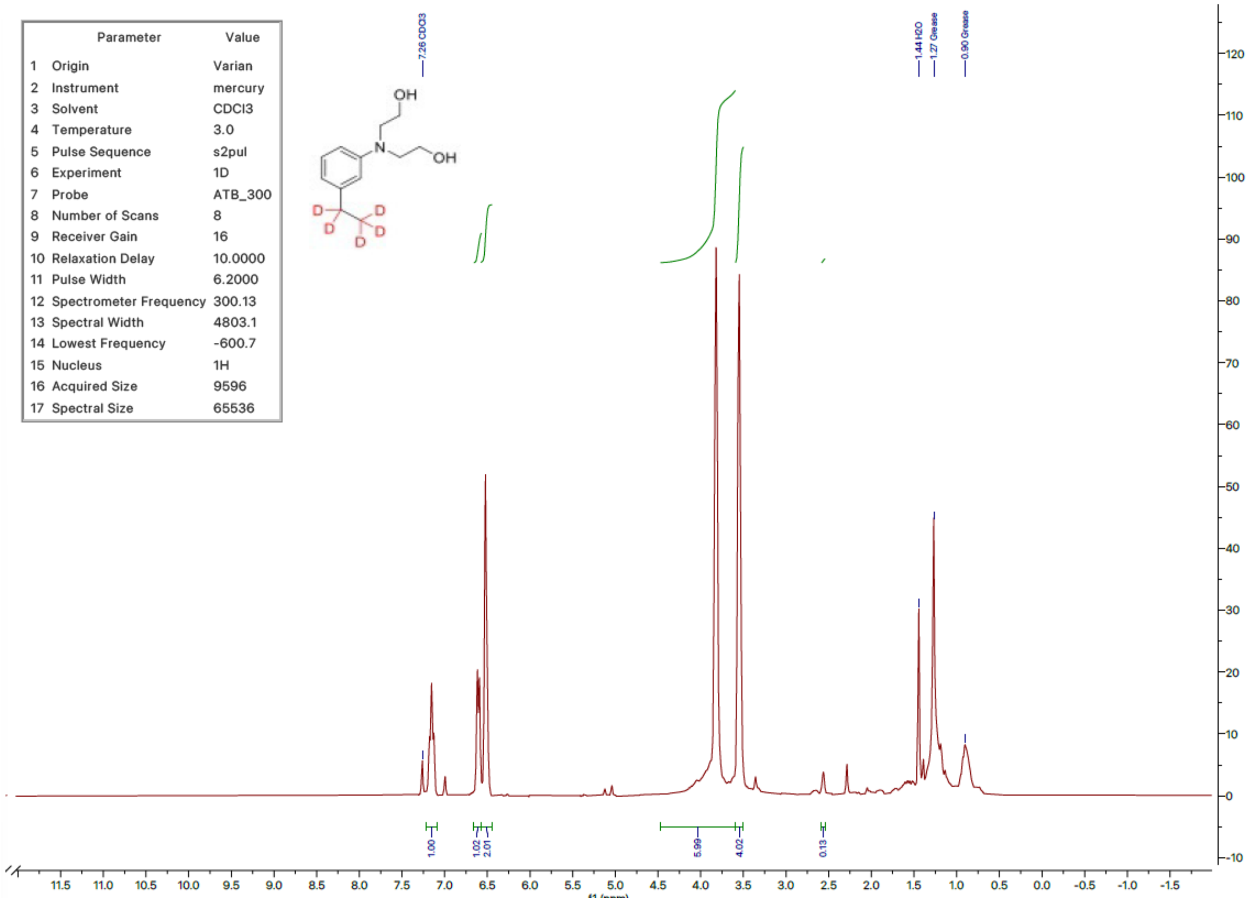

Figure S12. ^{1}H NMR of 6b in $CDCl_3$

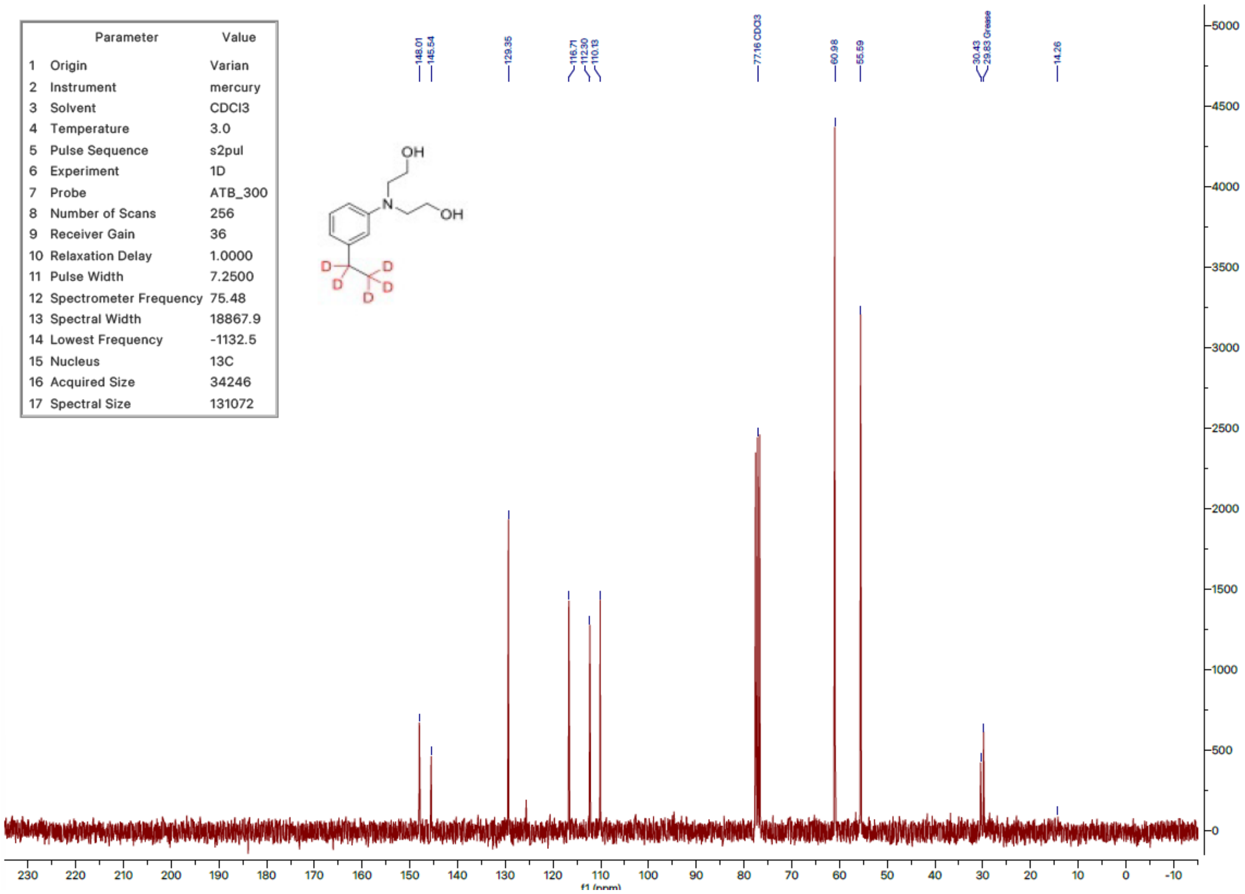

Figure S13. ^{13}C NMR of 7b in $CDCl_3$

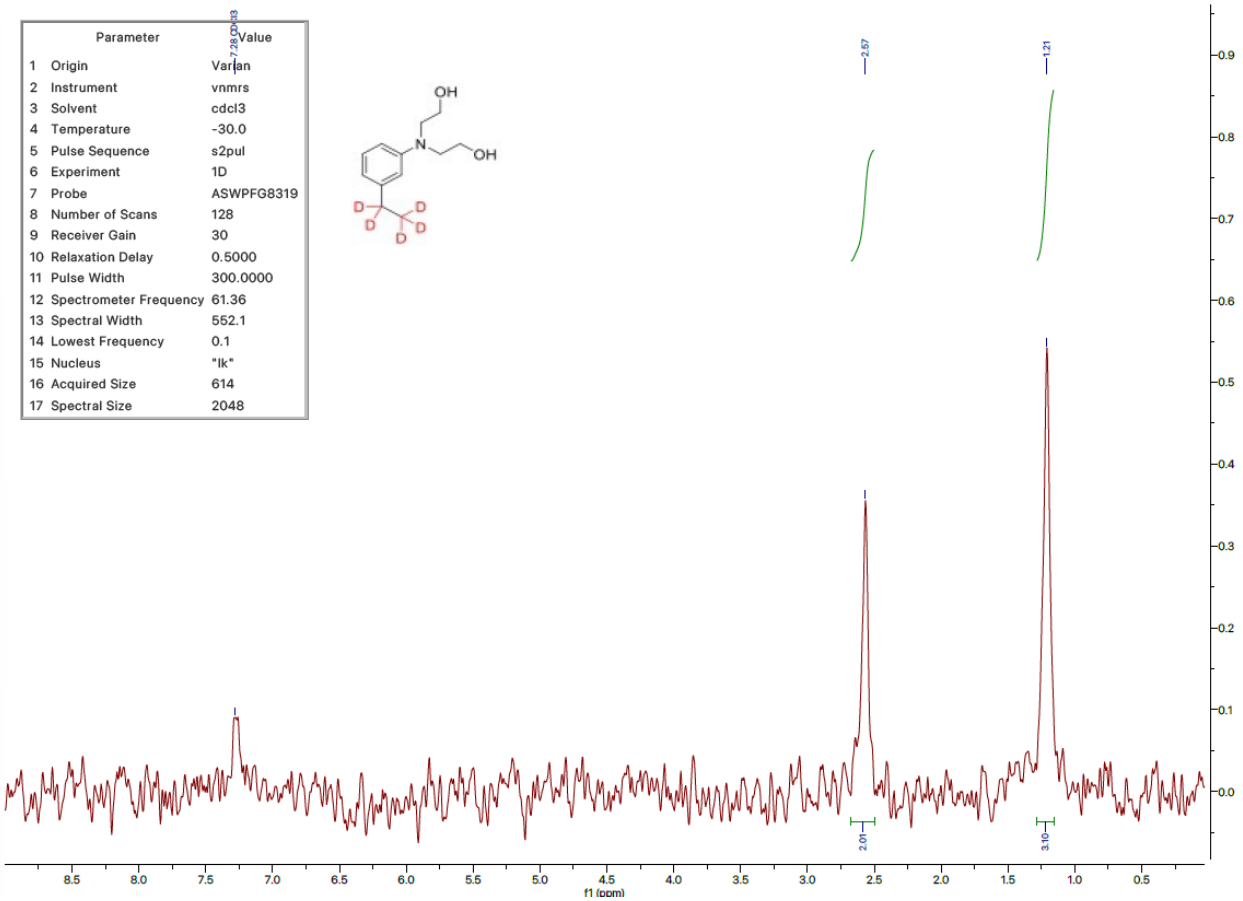

Figure S14. ^{1}H NMR of 7b in CDCl3

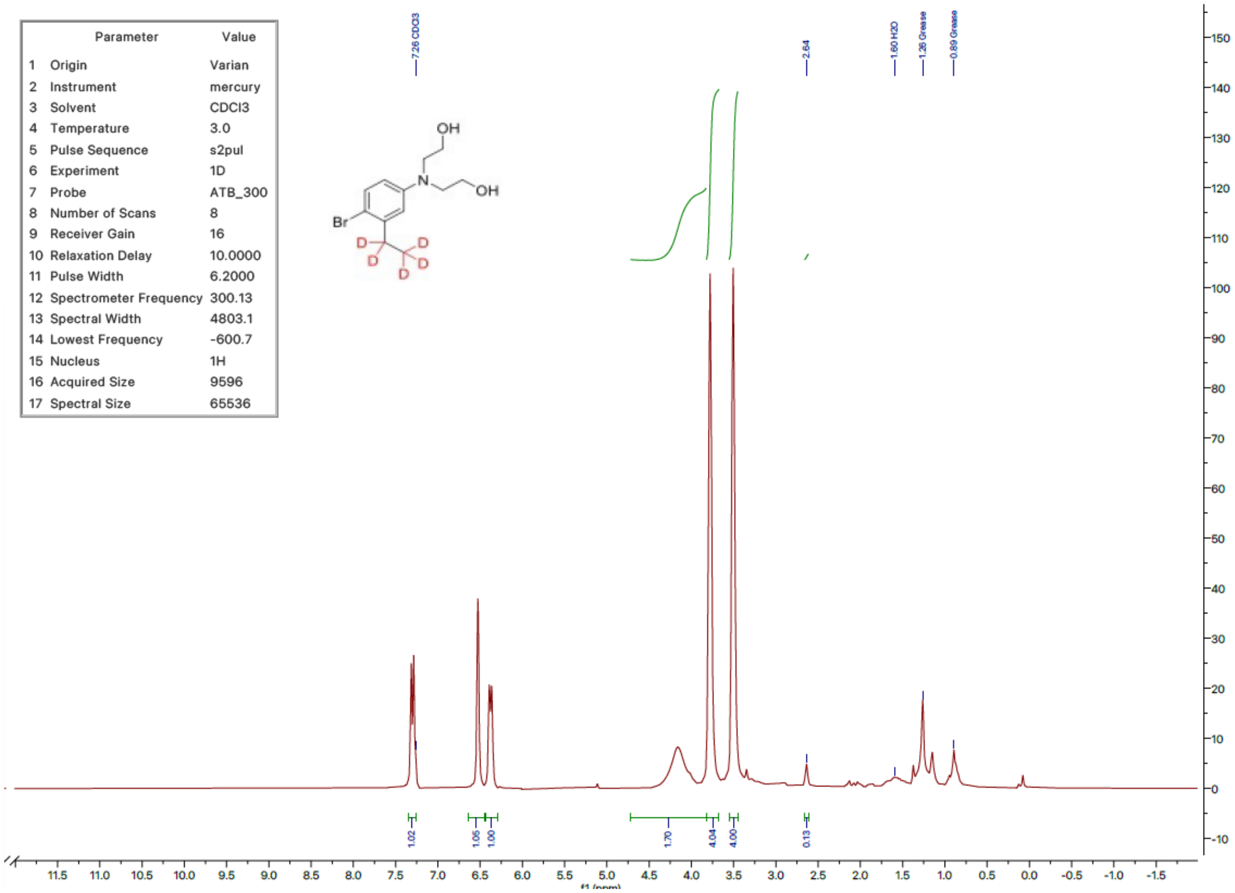

Figure S15. ^{1}H NMR of 8b in $CDCl_3$

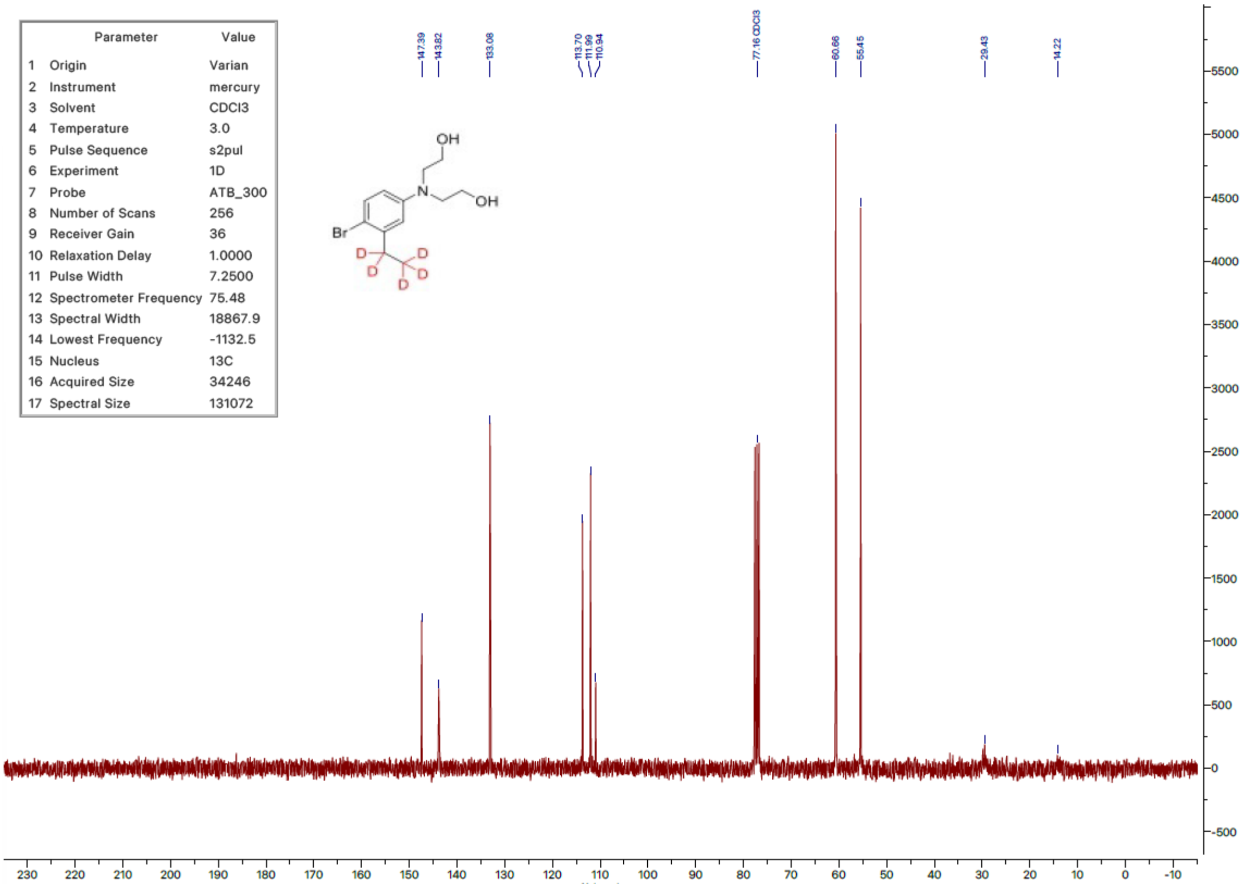

Figure S16. ^{13}C NMR of 8b in $CDCl_3$

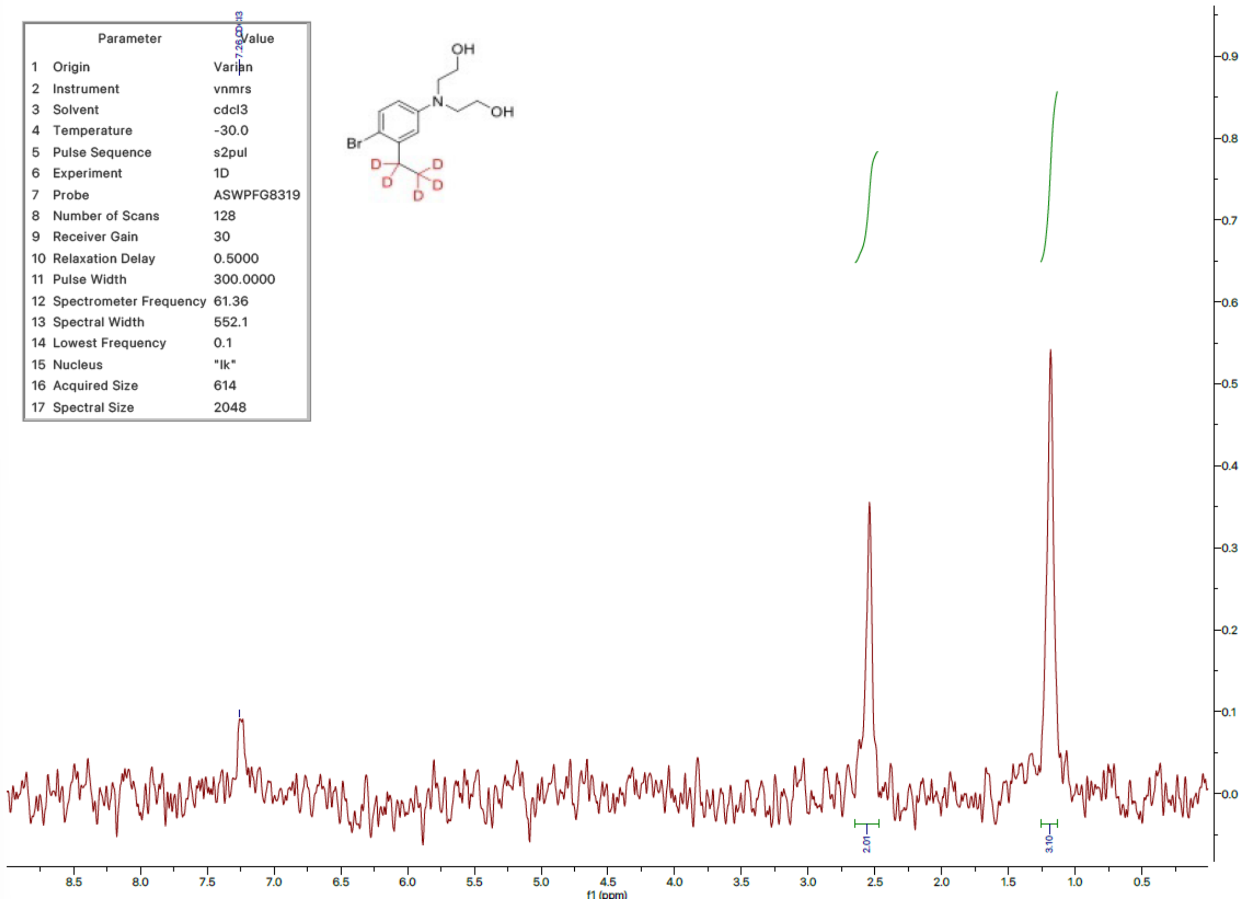

Figure S35. ^{1}H NMR of 8b in $CDCl_3$

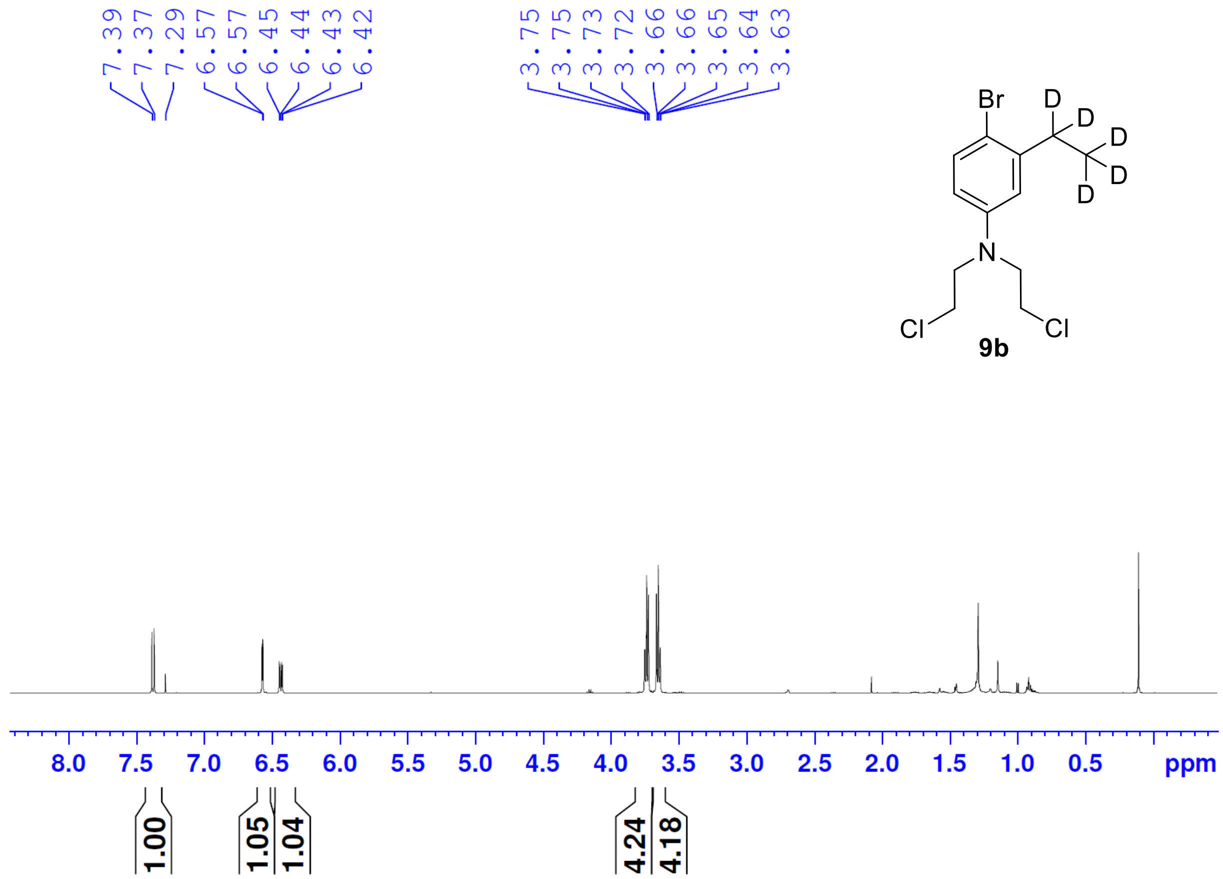

Figure S36. ^{1}H NMR in $CDCl_3$.

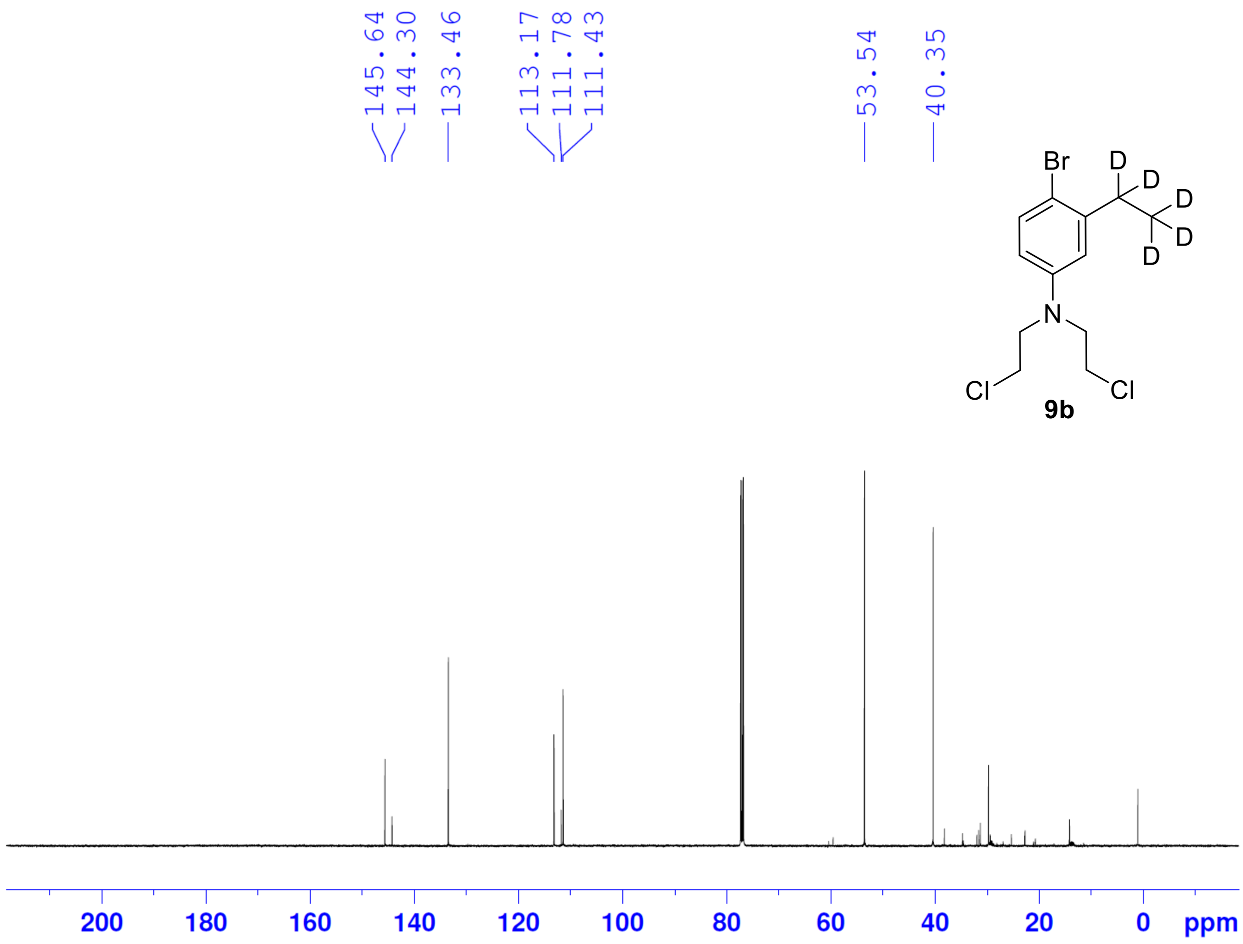

Figure S37. ^{13}C NMR in $CDCl_3$.

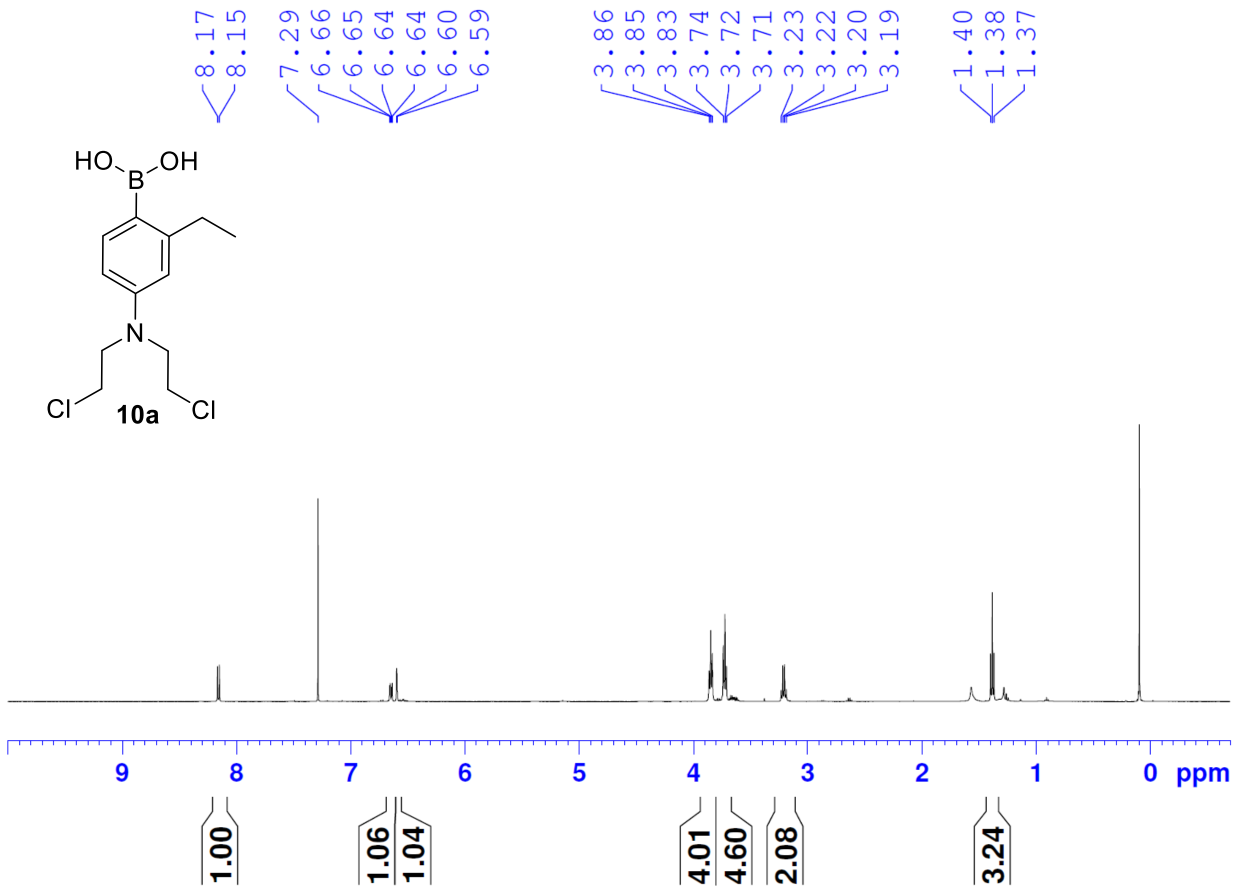

Figure S38. ^{1}H NMR in $CDCl_3$.

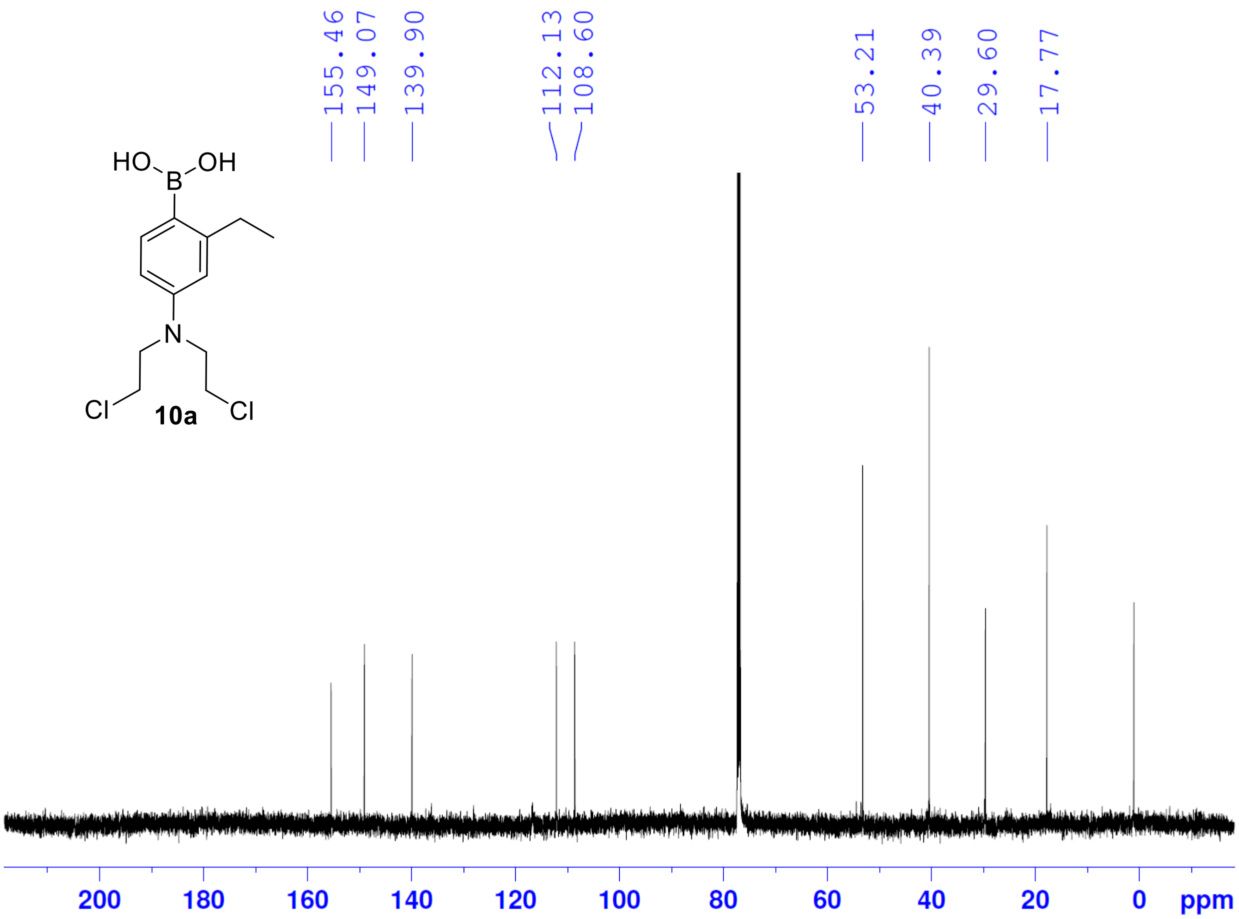

Figure S39. ^{13}C NMR in $CDCl_3$.

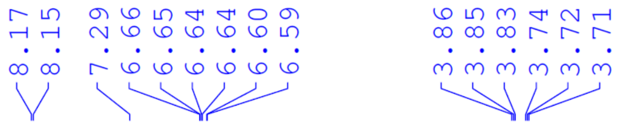

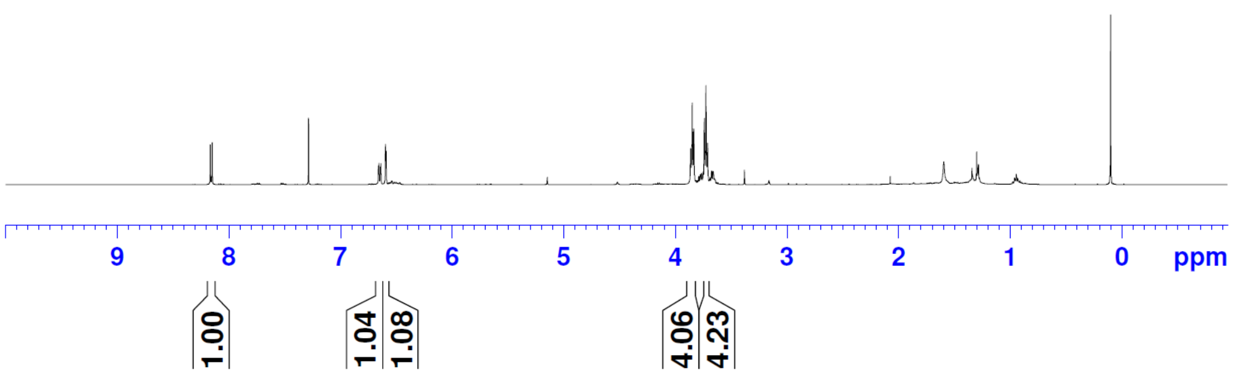

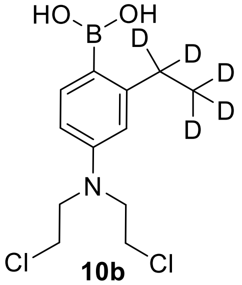

10b

Figure S40. ^{1}H NMR in $CDCl_3$.

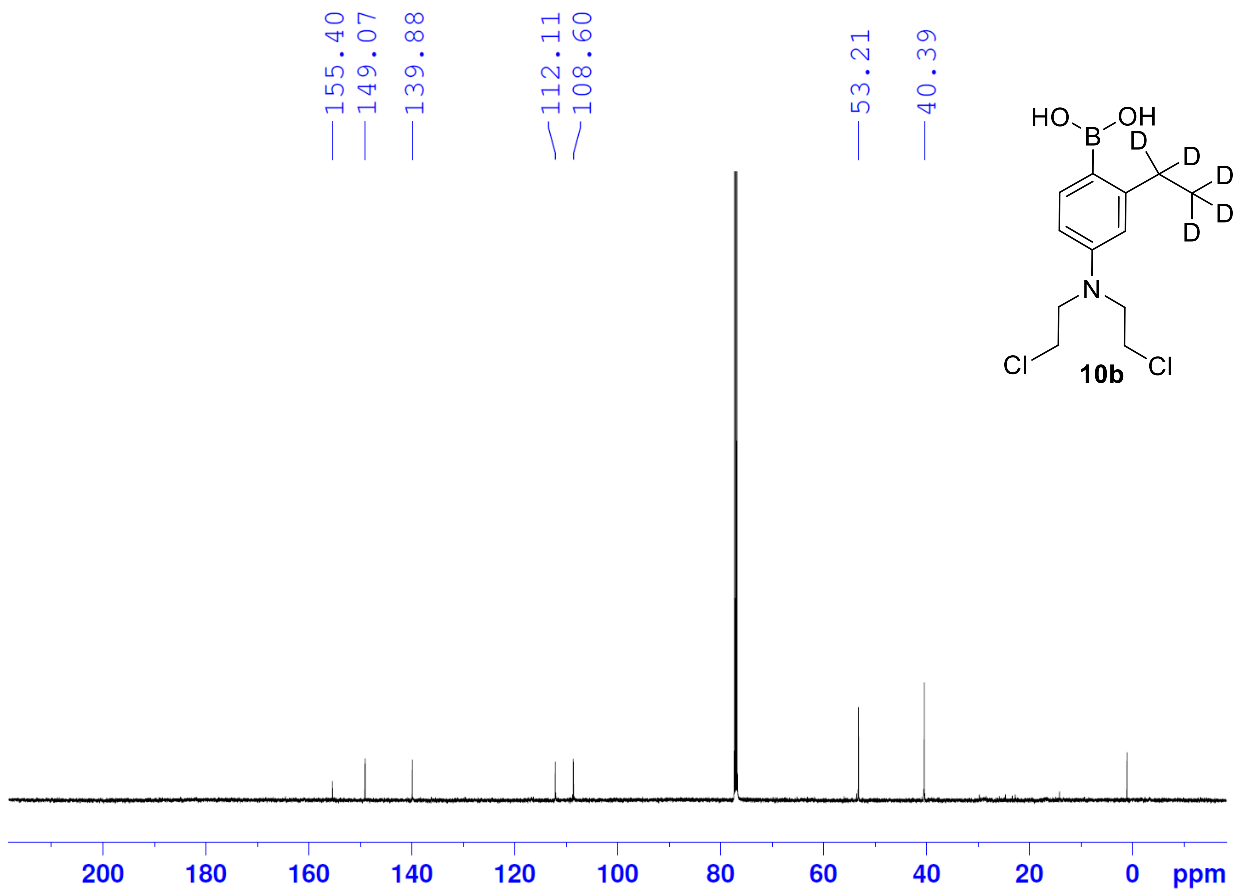

Figure S41. ^{13}C NMR in $CDCl_3$.

7. HRMS Spectra of Synthesized Molecules

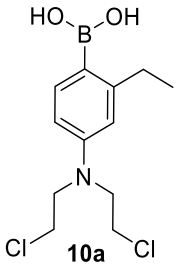

10a

Score	Pred. (M)	Pred. m/z	Meas. m/z	Diff. (mDa)	Formulae (M)	Ion	Diff. (ppm)	Iso Score	DBE
93.57	289.08076	290.08804	290.08897	0.93	C12 H18 N O2 B Cl2	[M+H]+	3.206	95.27	3.5

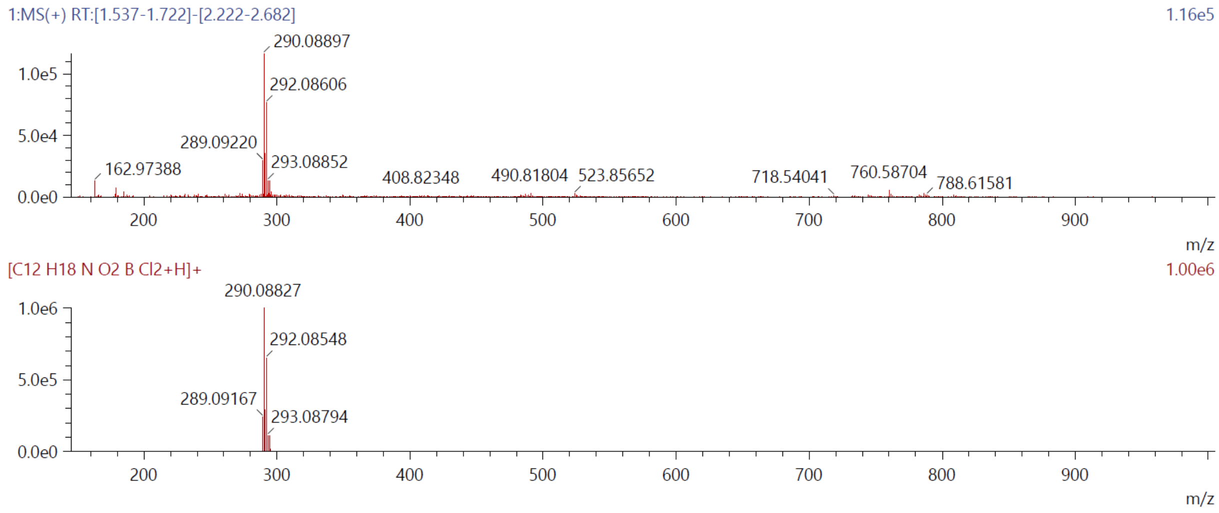

Figure S42. Q-TOF-MS (ESI) of compound 10a.

HRMS-ESI (+) (*m/z*): $[M+H]^+$ calcd. for $C_{12}H_{19}NO_2BCl_2^+$ 290.08804; found 290.08897.

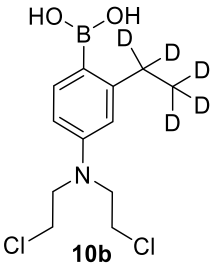

10b

Score	Pred. (M)	Pred. m/z	Meas. m/z	Diff. (mDa)	Formulae (M)	Ion	Diff. (ppm)	Iso Score	DBE
15.88	294.11215	295.11942	295.10384	-15.58	C12 H13 N O2 2H5 B Cl2	[M+H]+	-52.792	17.65	6.0

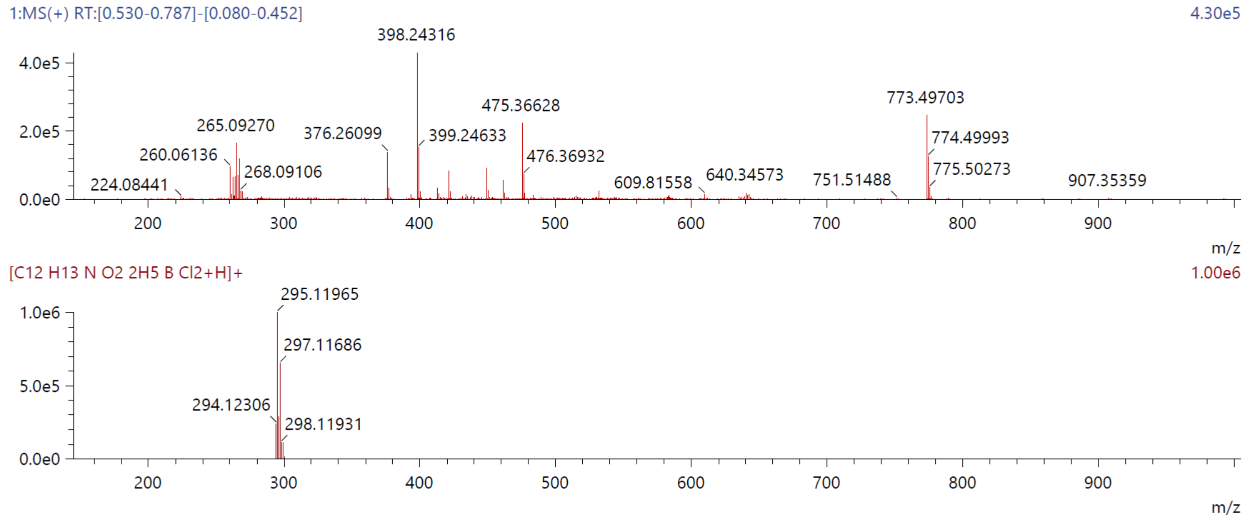

Figure S43. Q-TOF-MS (ESI) of compound 10b.

HRMS-ESI (+) (*m/z*): $[M+H]^+$ calcd. for $C_{12}H_{14}D_5NO_2BCl_2^+$ 295.11942; found 295.11965.

8. Purity of final products: 10a and 10b

Purity of 10a: 96%

Analysis Report

<Sample Information>

Sample Name	: Prodrug-d0		
Sample ID	: PT1		
Data Filename	: 1182022_002.lcd		
Method Filename	: 2022-11-7 Prodrug-H gradient 2 AA BB normal flow.lcm		
Batch Filename	: 2022-11-8 prodrug-H purity.lcb		
Vial #	: 3-3	Sample Type	: Unknown
Injection Volume	: 0.5 uL		
Date Acquired	: 11/8/2022 9:07:01 AM	Acquired by	: Eron Saxon
Date Processed	: 11/8/2022 9:54:49 AM	Processed by	: Eron Saxon

<Chromatogram>

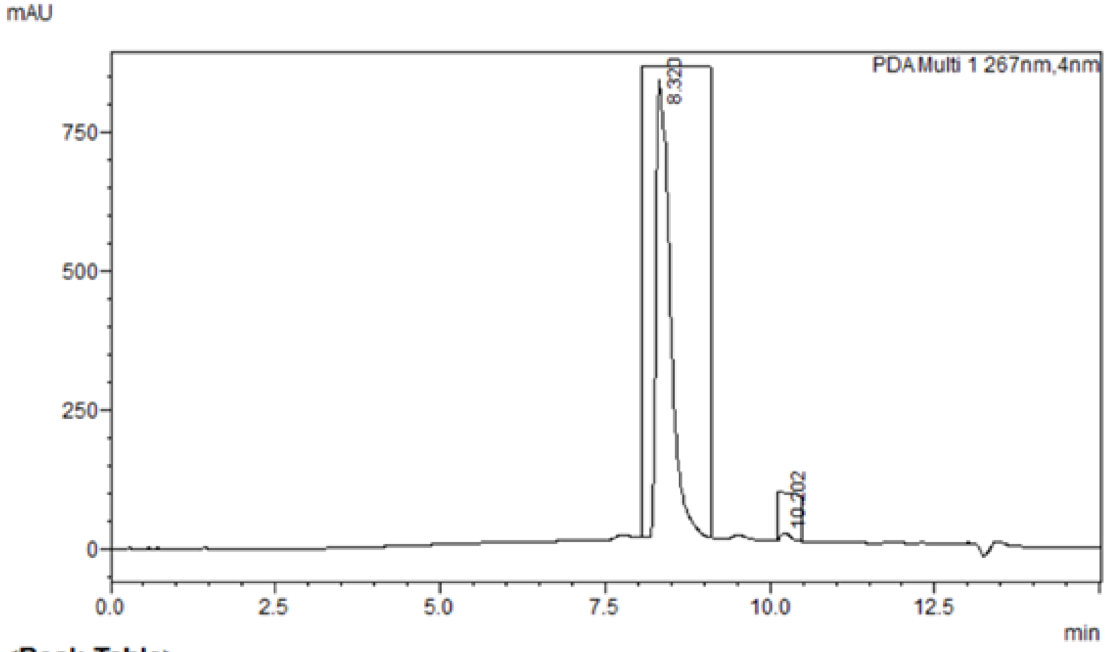

<Peak Table>

1182022_002.lcd

PDA Ch1 267nm

Peak#	Ret. Time	Area	Height	Area%	Height%	Area/Height
1	8.320	-41217247	-25776	95.835	25.885	1599.072
2	10.202	-1791378	-73803	4.165	74.115	24.272
Total		-43008624	-99579	100.000	100.000	

Figure S46. The purity of prodrug 10a (96%) determined by an ACQUITY CSH C18 column (2.1 mm × 50 mm, 1.7 µm particle size) using gradient: 0 – 10 min 5%-100% MeCN in A, 10.0-12.0 min 100% MeCN in A, 12.0-13.0 min 100%-5% MeCN in A, 13.0-15.0 min 5% MeCN in A at a flow rate of 0.5 mL/min (solution A: water).

Purity of 10b: 98%

<Sample Information>

Sample Name	: Prodrug-d0		
Sample ID	: PT1		
Data Filename	: 1192022_002.lcd		
Method Filename	: 2022-11-7 Prodrug-H gradient 2 AA BB normal flow.lcm		
Batch Filename	: 2022-11-9 prodrug-d4 purity.lcb		
Vial #	: 5-3	Sample Type	: Unknown
Injection Volume	: 1 uL		
Date Acquired	: 11/9/2022 11:33:29 AM	Acquired by	: Eron Saxon
Date Processed	: 11/9/2022 2:39:48 PM	Processed by	: Eron Saxon

<Chromatogram>

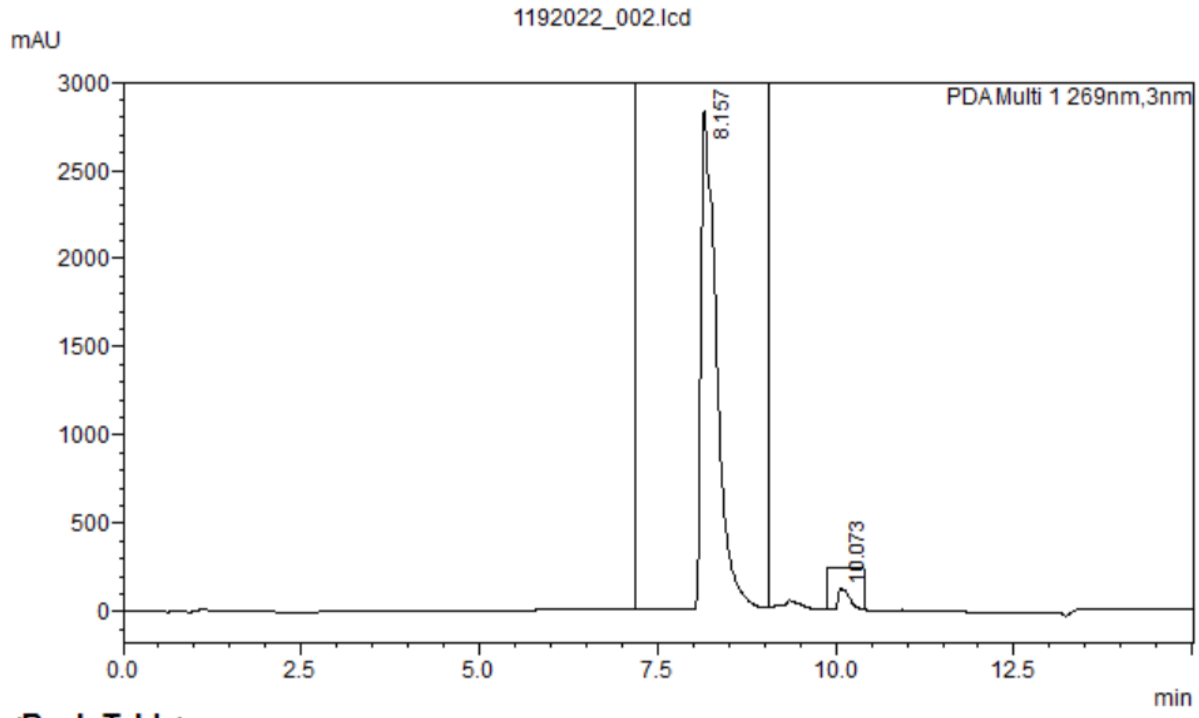

<Peak Table>

1192022_002.lcd

PDA Ch1 269nm

Peak#	Ret. Time	Area	Height	Area%	Area/Height
1	8.157	-302070795	-246629	97.965	1224.800
2	10.073	-6274214	-123940	2.035	50.623
Total		-308345008	-370569	100.000	

Figure S47. The purity of prodrug 10b (98%) determined by an ACQUITY CSH C18 column (2.1 mm × 50 mm, 1.7 µm particle size) using gradient: 0 – 10 min 5%-100% MeCN in A, 10.0-12.0 min 100% MeCN in A, 12.0-13.0 min 100%-5% MeCN in A, 13.0-15.0 min 5% MeCN in A at a flow rate of 0.5 mL/min (solution A: water).

Reference.

1. [illegible] *Med Chem* [illegible]
